# *Brucella melitensis *VjbR and C_12_-HSL regulons: contributions of the *N*-dodecanoyl homoserine lactone signaling molecule and LuxR homologue VjbR to gene expression

**DOI:** 10.1186/1471-2180-10-167

**Published:** 2010-06-08

**Authors:** Jenni N Weeks, Cristi L Galindo, Kenneth L Drake, Garry L Adams, Harold R Garner, Thomas A Ficht

**Affiliations:** 1 Department of Veterinary Pathobiology, College of Veterinary Medicine, Texas A & M University, College Station, Tx, 77843-4467, USA; 2 Department of Infectious Diseases, St. Jude Children's Research Hospital, Memphis, Tn, 38105, USA; 3 Virginia Bioinformatics Institute, Virginia Polytechnic and State University, Blacksburg, Va, 02461-0477, USA; 4 Seralogix, Inc., Austin, Tx, 78730, USA

## Abstract

**Background:**

Quorum sensing is a communication system that regulates gene expression in response to population density and often regulates virulence determinants. Deletion of the *luxR *homologue *vjbR *highly attenuates intracellular survival of *Brucella melitensis *and has been interpreted to be an indication of a role for QS in *Brucella *infection. Confirmation for such a role was suggested, but not confirmed, by the demonstrated *in vitro *synthesis of an auto-inducer (AI) by *Brucella *cultures. In an effort to further delineate the role of VjbR to virulence and survival, gene expression under the control of VjbR and AI was characterized using microarray analysis.

**Results:**

Analyses of wildtype *B. melitensis *and isogenic Δ*vjbR *transciptomes, grown in the presence and absence of exogenous *N*-dodecanoyl homoserine lactone (C_12_-HSL), revealed a temporal pattern of gene regulation with variances detected at exponential and stationary growth phases. Comparison of VjbR and C_12_-HSL transcriptomes indicated the shared regulation of 127 genes with all but 3 genes inversely regulated, suggesting that C_12_-HSL functions via VjbR in this case to reverse gene expression at these loci. Additional analysis using a Δ*vjbR *mutant revealed that AHL also altered gene expression in the absence of VjbR, up-regulating expression of 48 genes and a *luxR *homologue *blxR *93-fold at stationary growth phase. Gene expression alterations include previously un-described adhesins, proteases, antibiotic and toxin resistance genes, stress survival aids, transporters, membrane biogenesis genes, amino acid metabolism and transport, transcriptional regulators, energy production genes, and the previously reported *fliF *and *virB *operons.

**Conclusions:**

VjbR and C_12_-HSL regulate expression of a large and diverse number of genes. Many genes identified as virulence factors in other bacterial pathogens were found to be differently expressed, suggesting an important contribution to intracellular survival of *Brucella*. From these data, we conclude that VjbR and C_12_-HSL contribute to virulence and survival by regulating expression of virulence mechanisms and thus controlling the ability of the bacteria to survive within the host cell. A likely scenario occurs via QS, however, operation of such a mechanism remains to be demonstrated.

## Background

*Brucella *spp. are Gram-negative, non-motile, facultative intracellular bacterial pathogens that are the etiologic agents of brucellosis, causing abortion and sterility in a broad range of domestic and wild animals. Furthermore, brucellosis is a chronic zoonotic disease characterized in humans by undulant fever, arthritic pain and neurological disorders. *Brucella *virulence relies upon the ability to enter phagocytic and non-phagocytic cells, control the host's intracellular trafficking to avoid lysosomal degradation, and replicate in a *Brucella*-containing vacuole (brucellosome) without restricting host cell functions or inducing programmed death [1-3]. Although a few genes are directly attributed to the survival and intracellular trafficking of *Brucella *in the host cell (e.g., cyclic β-(1,2) glucan, lipopolysaccharide and the type IV secretion system (T4SS)), many aspects of the intracellular lifestyle remain unresolved [4-6].

Quorum sensing (QS), a communication system of bacteria, has been shown to coordinate group behavior in a density dependent manner by regulating gene expression; including secretion systems, biofilm formation, AI production, and cell division [7-10]. QS typically follows production of a diffusible signaling molecule or autoinducer (AI) acyl-homoserine lactone (AHL). Among proteobacteria, the larger family to which *Brucella *belong, the AHL signal is synthesized by *luxI*, and shown to interact with the transcriptional regulator LuxR to cooperatively modulate gene expression [9]. In addition to an AHL signal, LuxR regulatory activity can be modulated by phosphorylation (*fixJ*), contain multiple ligand binding sites (*malT*), or LuxR can function as an autonomous effector without a regulatory domain (*gerE*) [11-13].

Two LuxR-like transcriptional regulators, VjbR and BlxR (or also referred to as BabR) have been identified in *Brucella melitensis *[14,15]. VjbR was shown to positively influence expression of the T4SS and flagellar genes, both of which contribute to *B. melitensis *virulence and survival [14]. Although an AHL signal *N-*dodecanoyl homoserine lactone (C_12_-HSL) has been purified from *Brucella *culture supernatants, the gene responsible for the production of this AHL (*luxI*) has not yet been identified [16]. One possible explanation for the apparent absence of *luxI *homologues is that *Brucella *contains a novel AHL synthetase that remains to be identified. The fact that both LuxR homologues respond to C_12_-HSL by altering the expression of virulence determinants is also consistent with a role for the autoinducer in regulating expression of genes necessary for intracellular survival [17,18]. Specifically, expression of the *virB *and *flgE *operons are repressed by the addition of exogenous C_12_-HSL [14,16]. The results reported here extend those observations and suggest that C_12_-HSL acts as a global repressor of gene expression via interaction with VjbR while functioning to activate expression of other loci independent of VjbR.

In the present study, we sought to identify additional regulatory targets of the putative QS components VjbR and C_12_-HSL in an effort to identify novel virulence factors to confirm a role for QS in intracellular survival. Custom *B. melitensis *70-mer oligonucleotide microarrays were utilized to characterize gene expression. Comparison of transcript levels from *B. melitensis *wildtype and a *vjbR *deletion mutant, with and without the addition of exogenous C_12_-HSL revealed a large number of genes not previously shown to be regulated in *B. melitensis*, including those involved in numerous metabolic pathways and putative virulence genes (e.g., adhesins, proteases, lipoproteins, outer membrane proteins, secretion systems and potential effector proteins). Additionally, results confirmed earlier findings of genes regulated by these components, validating the microarray approach for identification of genes that may contribute to intracellular survival and virulence.

## Methods

### Bacteria, macrophage strains and growth conditions

*Escherichia coli *DH5α™-T1^R ^competent cells were used for cloning and routinely grown on Luria-Bertani (LB, Difco Laboratories) overnight at 37°C with supplemental kanamycin (100 mg/l) or carbenicillin (100 mg/l) as needed. *B. melitensis *16M was grown on tryptic soy agar or broth (TSA or TSB) and J774A.1 murine macrophage-like cells were maintained in T-75 flasks in Dulbecco's modified Eagle's medium, HEPES modification (DMEM), supplemented with 1× MEM non-essential amino acids (Sigma, St Louis, MO), 0.37% sodium bicarbonate and 10% fetal bovine serum at 37°C with 5% CO_2_. All work with live *B. melitensis *was performed in a biosafety level 3 laboratory at Texas A&M University College Station, per CDC approved standard operating procedures. All bacterialstrains used are listed in Additional File 1, Table S1.

### Generation of gene replacement and deletion mutants

LuxR-like proteins were identified in *B. melitensis *using NCBI BLAST protein homology searches http://www.ncbi.nlm.nih.gov/. *B. melitensis *16M *luxR *gene replacement and deletion mutations were created as previously described by our laboratory, with plasmids and strains generated described in Additional File 1, Table S1 and primers for PCR applications listed in Additional File 2, Table S2 [19]. For complementation of the Δ*vjbR *mutation, gene locus BMEII1116 was amplified by PCR primers TAF588 and TAF589, cloned into pMR10-Kan *Xba*I sites, and electroporated into *B. melitensis *16MΔ*vjbR *(Additional File 1, Table S1 and Additional File 2, Table S2).

### Gentamycin protection assay

J774A.1 cells were seeded into 24-well plates at a density of 2.5 × 10^5 ^CFU/well and allowed to rest for 24 hours in DMEM. J774A.1 cells were infected with *B. melitensis *16M or mutant strains in individual wells at an MOI of 20. Following infection, monolayers were centrifuged (200 × g) for 5 min and incubated for 20 minutes. Infected monolayers were washed 3 × in Peptone Saline (1% Bacto-Peptone and 0.5% NaCl), and incubated in DMEM supplemented with gentamycin (40 μg/ml) for 1 hour. To collect internalized bacteria at time 0 and 48 hours post-infection, macrophages were lysed in 0.5% Tween-20 and serial dilutions were plated to determine bacterial colony forming units (CFU).

### RNA collection

Cultures were grown in *Brucella *Broth at 37°C with agitation. Cultures for the AHL experiments were grown with the addition of exogenous *N*-dodecanoylhomoserine lactone (C_12_-HSL, Sigma, St. Louis, MO) added at inoculation (50 ng/ml) dissolved in DMSO (at a final concentration of 0.008%) [16]. Total RNA was extracted at mid-exponential (OD_600 _= 0.4) and early stationary (OD_600 _= 1.5) growth phases by hot acidic phenol extraction, as previously described [20]. Contaminating DNA was degraded by incubation with DNAseI (Qiagen, Valencia, CA) following manufacturer's instructions and purified using the HighPure RNA isolation kit (Roche, Indianapolis, IN). RNA integrity, purity and concentration were evaluated using a 2100 bioanalyzer (Agilent, Santa Clara CA), electrophoresis, and the Nanodrop^® ^ND-1000 (Nanodrop, Wilmington, DE).

### DNA and RNA labeling for microarrays

*B. melitensis *16M genomic DNA was processed into cDNA using the BioPrime^® ^Plus Array CGH Indirect Genomic Labeling System (Invitrogen, Carlsbad, CA) and purified using PCR purification columns (Qiagen, Valencia, CA) following the manufacturer's instructions and eluted in 0.1× of the supplied elution buffer.

The cDNA synthesis from total RNA was produced using SuperScript III reverse transcriptase kit following manufacturer instructions (Invitrogen, Carlsbad, CA). Reactions were subsequently purified with PCR Purification columns (Qiagen, Valencia, CA) using a modified wash (5 mM KPO_4 _(pH 8.0) and 80% ethanol) and incremental elution with 4 mM KPO_4_, pH 8.5. Alexa-Fluor 555 (Invitrogen, Carlsbad, CA) was coupled to the RNA-derived cDNA following the procedure outlined in the BioPrime^® ^Plus Array CGH Indirect Genomic Labeling System (Invitrogen, Carlsbad, CA) and purified using PCR purification columns (Qiagen, Valencia, CA). Labeled RNA samples were dried completely and re-suspended in ddH_2_O immediately before hybridization to the microarrays.

### Microarray construction

Unique 70-mer oligonucleotides (Sigma, St. Louis, MO) representing 3,227 ORFs of *B. melitensis *16M and unique sequences from *B. abortus *and *B. suis *were suspended in 3× SSC (Ambion, Austin, TX) at 40 μM. The oligonucleotides were spotted in quadruplicate onto ultraGAP glass slides (Corning, Corning, NY) by a custom-built robotic arrayer (Magna Arrayer) assembled at Dr. Stephen A. Johnston's lab at the University of Texas Southwestern Medical Center (Dallas, TX). The printed slides were steamed, UV cross-linked, and stored in a desiccator until use.

### Microarray pre-hybridization, hybridization and washing

Printed slides were submerged in 0.2% SDS for 2 minutes and washed 3× in ddH_2_O before incubation in prehybridization solution (5× SSC, 0.1% SDS and 1% BSA) at 45°C for 45 minutes. Next, slides were washed 5× in ddH_2_O, rinsed with isopropanol, and immediately dried by centrifugation at 200 × g for 2 minutes at room temperature. The labeled cDNA mix was combined with 1× hybridization buffer (25% formamide, 1× SSC and 0.1%SDS) and applied to the microarray in conjunction with a 22 × 60 mm LifterSlip (Erie Scientific, Portsmouth, NH). The microarray slides were hybridized at 42°C for approximately 21 hours in a sealed hybridization chamber with moisture (Corning, Corning, NY), and subsequently washed at room temperature with agitation in 2× SSC and 0.2% SDS (pre-heated to 42°C) for 10 minutes, 5 minutes in 2× SSC, followed by 0.2× SSC for 5 minutes, and dried by centrifugation for 2 minutes (200 × g) at room temperature.

### Microarray data acquisition and analysis

Array slides were scanned using GenePix 4100A (Molecular Devices, Sunnyvale, CA) and GenePix 6.1 Pro software. Seralogix, Inc. (Austin, TX) performed microarray analysis, normalizing the data and identifying differentially expressed genes by a two-tail z-score level greater than ± 1.96, equating to a confidence level of 95%. Additionally, the NIH/NIAID WRCE bioinformatics core performed microarray analysis as follows: GeneSifter (VizX Labs, Seattle, WA) was used to perform normalization based on the global mean and genes with alterations of least a 1.5-fold, with a *p *value of 0.05 or less based on Student's *t*-test were deemed as statistically significant. Any gene that was considered statistically significant based on Student's *t*-test but not by the z-score criteria were further expected to be at least 50% greater in magnitude (e.g., 1.5-fold greater) than the fold-change observed between any two biological replicate samples. All gene expression data have been deposited in NCBI's Gene Expression Omnibus and are accessible through GEO Series accession number GSE13634.

### Quantitative real time PCR

Taqman^® ^universal probes and primer pairs (Additional File 2, Table S2) were selected using Roche's Universal Probe Library and probefinder software http://www.universalprobelibrary.com. RNA was reverse transcribed to cDNA using the Transcriptor First Strand cDNA synthesis kit (Roche, Indianapolis, IN) andPCR reactions consisted of 1× TaqMan^® ^universal PCR master mix, no AmpErase^® ^UNG (Applied Biosystems, Foster City, CA), 200 nM of each primer and 100 nM of probe. With the exception of BMEI1758, genes were selected at random for quantitative real time PCR (qRT-PCR) verification, and were performed in triplicate for each sample within a plate and repeated 3× using the 7500 Real Time PCR System (Applied Biosystems, Foster City, CA). Gene expression was normalized to that of 16s rRNA and the fold-change calculated using the comparative threshold method [21].

### Screen for a putative AHL synthase

Fifteen *B. melitensis *genetic loci and *P. aeruginosa lasI *and *rhlI *were amplified by PCR, cloned into *BamH*I sites in the pET-11a expression vector and transformed by heat-shock into *E. coli *BL21-Gold(DE3) cells (Additional File 1, Table S1 and Additional File 2, Table S2). The resulting clones were cross streaked on LB agar supplemented with 2 mM IPTG with *E. coli *JLD271 + pAL105 and pAL106 for detection of C_12_-HSL production, and *E. coli *JLD271 + pAL101 and pAL102 for detection of C_4_-HSL production (Additional File 1, Table S1). Cross-streaks were incubated at 37°C for 2-8 hours, and luminescence was detected using the FluorChem Imaging System (Alpha-Innotech, San Leandro, CA) at varied exposure times.

## Results and Discussion

### Identification and screening for attenuation of Δ*luxR *mutants in J774A.1 macrophage-like cells

A *luxR*-like gene, *vjbR*, was identified in a mutagenesis screen conducted by this laboratory and others [22]. More recently, a second *luxR-*like gene, *blxR *(or *babR*), has also been identified and characterized [15,23]. These two homologues, VjbR and BlxR, contain the two domains associated with QS LuxR proteins (i.e., autoinducer binding domain and LuxR DNA binding domain). BLAST protein homology searches with the LuxR-like proteins identified three additional proteins that contain significant similarity to the LuxR helix-turn-helix (HTH) DNA binding domain but do not contain the AHL binding domain. All 5 *B. melitensis *LuxR-like proteins exhibit similar levels of relatedness to *Agrobacterium tumefaciens *TraR homolog (29-34%) and canonical LuxR homolog LasR from *Pseudomonas aeruginosa *(29-43%). Despite the absence of a characterized activation domain, evaluation of these three proteins was pursued due to their similarity with LuxR homologs best characterized in *Vibrio harveyi *that act autonomously or via phosphorylation/dephosphorylation to alter gene expression from selected loci [24,25].

**Figure 1:**
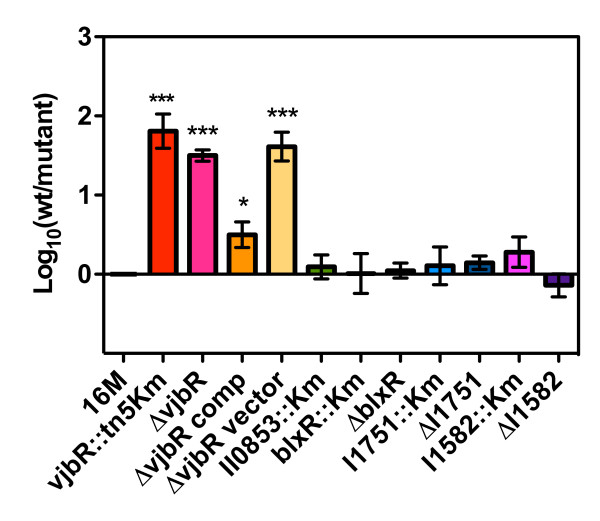
**Intracellular survival of *B. melitensis *16M (wt), *vjbR *mutant (Δ*vjbR *and *vjbR*::m *Tn*5), complemented Δ*vjbR *(Δ*vjbR*comp and Δ*vjbRvector*), Δ*blxR *mutant, and 3 additional *luxR*-like mutants in J774A.1 murine macrophage-like cells**. The attenuation was measured as the log difference between the CFU recoveries of the mutant compared to wildtype from infected macrophages at 48 hours post infection. Data shown is the averaged CFU recovery from at least 3 independent experiments, each performed in triplicate. Error bars represent the SEM and each mutant was compared to the wildtype by a Student's two tailed *t*-test, with the resulting *p *values as follows:*, *P *< .0.05; ***, *P *< 0.001. The *luxR *deletion mutant strains are identified by the BME gene locus ID tags, BME::Km representing the gene replacement mutant and ΔBME representing the gene deletion mutant.

Gene replacement and deletion mutations were created for all five homologues including the three newly discovered HTH LuxR DNA binding domain homologues (BME I1582, I1751 and II0853), *vjbR*, and *blxR *in *B. melitensis *16M and survival in J774A.1 macrophage-like cells was subsequently assessed by gentamycin protection assays. Confirming previous findings, intracellular survival was significantly reduced for both the *vjbR *transposon and deletion mutants and not for the *blxR *mutant, as indicated by CFU recovery after 48 hrs of infection (Fig. 1) [14,23]. Survival of the *vjbR *mutant was restored to nearly wildtype levels after complementation (Fig. 1). No significant difference in CFU was observed for the other three mutants when compared to wildtype infected cells, indicating that these homologues are either not required for intracellular replication in macrophages or there is functional redundancy among some of homologues (Fig. 1). A recent report presented evidence indicating that the Δ*blxR *and Δ*vjbR *mutants exhibited similar levels of attenuated intracellular survival in the RAW264.7 macrophage cells [15]. However, the Δ*blxR *mutant proved to be virulent in IRF1-/- knockout mice, with only a slight delay in mortality when compared to wildtype (10 days vs. 7.4, respectively) [15]. For comparison, all of the mice inoculated with the Δ*vjbR *mutant survived to at least day 14 [15]. Taken together the results suggest that the loss of *blxR *expression has only a modest effect on virulence/survival and the attenuated phenotype of the Δ*vjbR *mutant is more consistently observed.

### Microarray analysis indicates that *Brucella *putative quorum sensing components are global regulators of gene expression

To investigate the transcriptional effects resulting from a *vjbR *deletion and the addition of exogenous C_12_-HSL, RNA was isolated from wildtype *B. melitensis *16M, the isogenic Δ*vjbR*, and both strains with the addition of exogenous C_12_-HSL, at a logarithmic growth phase and an early stationary growth phase. The use of exogenous C_12_-HSL addition to cultures was selected because of the inability to eliminate the gene(s) responsible for C_12_-HSL production. Three independent RNA samples were harvested at each time point (exponential and early stationary growth phases) and hybridized with reference genomic DNA, which yielded a total of 24 microarrays.

**Figure 2:**
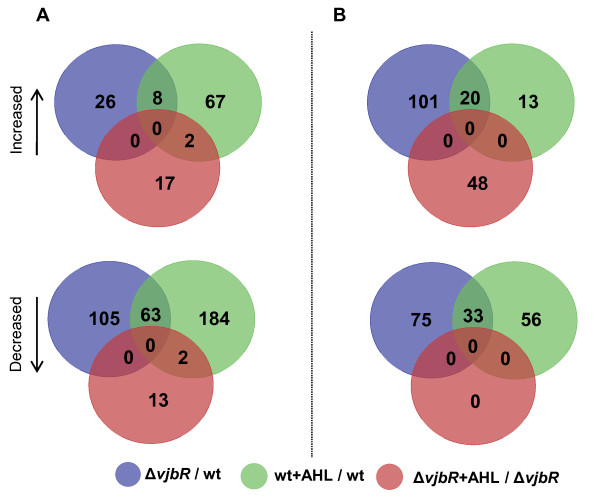
**Numbers and relationships of transcripts altered by the deletion of *vjbR *and/or treatment of C**_12_**-HSL**. Numbers represent the statistically significant transcripts found to be up or down-regulated by microarray analysis at the A) exponential growth phase (OD_600 _= 0.4) and B) stationary growth phase (OD_600 _= 1.5).

Microarray analysis revealed a total of 202 (Fig. 2A, blue circles) and 229 genes (Fig. 2B, blue circles) to be differentially expressed between wildtype and Δ*vjbR *cultures at exponential and stationary growth phases, respectively (details provided in Additional File 3, Table S3). This comprises 14% of the *B. melitensis *genome and is comparable to the value of 10% for LuxR-regulated genes previously predicted for in *P. aeruginosa *[26]. The majority of altered genes at the exponential phase were down-regulated (168 genes) in the absence of *vjbR*, while only 34 genes were up-regulated (Fig. 2A, blue circles). There were also a large number of down-regulated genes (108 genes) at the stationary phase; however, at this later time point there were also 121 genes that were specifically up-regulated (Fig. 2B, blue circles). When comparing wild-type cells with and without the addition of exogenous C_12_-HSL, the majority of genes were found to be down-regulated at both growth phases, 249 genes at exponential phase (Fig. 2A, green circle) and 89 genes at stationary phase (Fig. 2B, green circle). These data suggest that VjbR is primarily a promoter of gene expression at the exponential growth phase and acts as both a transcriptional repressor and activator at the stationary growth phase. Conversely, C_12_-HSL primarily represses gene expression at both growth phases.

Quantitative real time PCR (qRT-PCR) was performed to verify the changes in gene expression for 11 randomly selected genes found to be altered by the microarray analyses (Table 1). For consistency across the different transcriptional profiling assays, cDNA was synthesized from the same RNA extracts harvested for the microarray experiments. For the 11 selected genes, the relative transcript levels were comparable to the expression levels obtained from the microarray data.

**Table 1:** Quantitative real time PCR and corresponding microarray data of selected genes.

BME Loci	Gene Function	Condition (growth phase)	Change (Fold)	
			
			qRT-PCR	Microarray
I 0984	ABC-Type β-(1,2) Glucan Transporter	Δ*vjbR*/wt (ES)	-2.5	-2.1
II 0151	Flagellar M-Ring Protein, FliF	Δ*vjbR*/wt (ES)	-7.9	-2.2
II 1069	Adhesin, AidA	Δ*vjbR*/wt (SP)	-1.9	-1.5
I 0561	Membrane-Bound Lytic Murein Transglycosylase B	Δ*vjbR*/wt (SP)	-1.7	-2.0
II 0025	Attachment Mediating Protein VirB1	Δ*vjbR*/wt (SP)	-4.1	-2.6
I 0831	UDP-3-O-[3-hydroxymyristoyl] Glucosamine N-Acyltransferase	wt + AHL/wt (ES)	2.2	2.3
II 0151	Flagellar M-Ring Protein, FliF	wt + AHL/wt (ES)	-3.8	-2.1
II 0838	Succinoglycan Biosynthesis Transport Protein, ExoT	wt + AHL/wt (ES)	-1.7	-4.3
II 1116	LuxR Family Transcriptional Regulator, VjbR	wt + AHL/wt (SP)	-2.9	-
I 1758	LuxR Family Transcriptional Regulator, BlxR	wt + AHL/wt (SP)	27.5	-
I 0155	Putative Allantoin Permease	wt + AHL/wt (SP)	-1.7	-1.4
II 0025	Attachment Mediating Protein VirB1	wt + AHL/wt (SP)	-2.5	-2.2
II 0753	ABC-Type Sorbitol/Mannitol Transport Inner Membrane Protein	Δ*vjbR*/Δ*vjbR *+ AHL (ES)	1.5	2.5
I 1758	LuxR Family Transcriptional Regulator, BabR	Δ*vjbR*/Δ*vjbR*+AHL (SP)	99.5	-

A (-) indicates genes excluded for technical reasons or had a fold change of less than 1.5. qRT-PCR values were calculated by the ΔΔCt method normalized to 16s rRNA and are relative to the wildtype, averaged from 3 independently isolated samples, performed in triplicate in a minimum of three assays. ES, Exponential growth phase; SP, Stationary growth phase.

Recently, a *virB *promoter sequence was identified and confirmed to promote expression of downstream genes via VjbR [27]. With such a large number of transcriptional regulators found to be altered downstream of VjbR and by the addition of C_12_-HSL (Table 2), it is plausible that many of the gene alterations observed may be downstream events and not directly regulated by VjbR. To identify altered genes that are likely directly regulated by VjbR, microarray data from these studies were compared to the potential operons downstream of the predicted VjbR promoter sequences [27]. A total of 91 potential operons from the 144 previously predicted VjbR promoter sequences were found to be altered by a deletion of VjbR and/or treatment of wildtype cells with C_12_-HSL, comprised of 215 genes (Additional File 4, Table S4) [27]. A total of 11 promoters from the confirmed 15 found to be activated by VjbR in an *E. coli *model were identified in the microarray analyses conducted in this study, confirming the direct regulation of these particular operons (Additional File 4, Table S4) [27].

**Table 2:** Transcripts associated with gene regulation significantly altered between 16M and 16MΔ*vjbR*, with and without the treatment of C_12_-HSL to cells.

BME Loci	Gene Function	Exponential Growth Phase Change (fold)			Stationary Growth Phase Change (fold)			STM
			
		Δ*vjbR*/wt	wt+AHL/wt	Δ*vjbR*/Δ*vjbR*+AHL	Δ*vjbR*/wt	wt+AHL/wt	Δ*vjbR*/Δ*vjbR*+AHL	
I 0019	LacI Family	-2.9	-1.8^†^	-	1.9	1.5^†^	-	
I 0305	DeoR Family	-1.7	-1.7^†^	-	1.9	1.5^†^	-	[31]
I 0447	Leucine-Responsive Regulatory Protein	1.6	-	-	-2.4	-1.8	-	
I 0781	DNA-Directed RNA Polymerase A Subunit	2.4^†^	2.8	-	-	-	-	[34]
I 1383	AraC Family	-2.4	-1.5^†^	-	-	-1.7^†^	-	
I 1607	LuxR Family DNA Binding Domain	1.8^†^	3.0	-	-1.5^†^	-	-	
I 1631	TetR Family	-1.9	-2.1	-	-	-	-	
I 1700	Predicted Transcriptional Regulator	2.0	2.9	-	-	-	-	
II 0051	LuxR Family DNA Binding Domain	-1.9	-2.8	-	-	-	-	
II 0800	AraC Family	1.7	2.2^†^	-	-	-	-	
II 0854	CRP Family Transcriptional Regulator	-	1.6^†^	-	-1.5	-1.7	-	
II 0985	LacI Family	-2.5	-2.7^†^	-	-2.4	-	-	
II 1022	IclR Family	-1.5^†^	-1.8	-	-1.9	-2.1	-	
II 1098	AraC Family	-1.8	-2.8	-	1.9	1.5	-	
I 0446	MarR Family	1.9^†^	2.9	2.9^†^	-	-	-	
I 0518	Cold Shock Protein, CspA	1.6	-	-2.0^†^	1.7	-	-	
I 0720	Sugar Fermentation Stimulation Protein	-	-2.0	1.7^†^	-1.7^†^	-	1.5^†^	
I 0899	Phage-Related DNA Binding Protein	-1.8	-1.5^†^	-1.9^†^	1.6	-	-2.4^†^	
I 1098	AsnC Family	-1.7	-2.0	-1.6^†^	-1.6	-	-	
I 1291	AraC Family	-	-1.9	-1.7^†^	1.7	-	-	
I 1641	TetR Family	-	-	-2.7^†^	-1.7	-1.8	-	
I 1885	LysR Family	-	-1.8^†^	-2.3^†^	-1.6	-	-	
II 0127	IclR Family	-	1.6^†^	-	-1.8	-	1.6^†^	
II 0219	IclR Family	-3.2	-5.8	-3.8^†^	-1.5^†^	-	-	
II 0657	Transcription Elongation Factor	2.4^†^	3.1	-	-	-	2.4^†^	
II 0810	ArsR Family	-	2.0	-	1.8	1.6^†^	-2.3^†^	

A (-) indicates genes excluded for technical reasons or had a fold change of less than 1.5; ^† ^genes that did not pass the statistical significance test but showed an average alteration of at least 1.5-fold. Fold change values are the averaged log_2 _ratio of normalized signal values from two independent statistical analyses. Abbreviations as follows: STM, Signature Tagged Mutagenesis.

**Figure 3:**
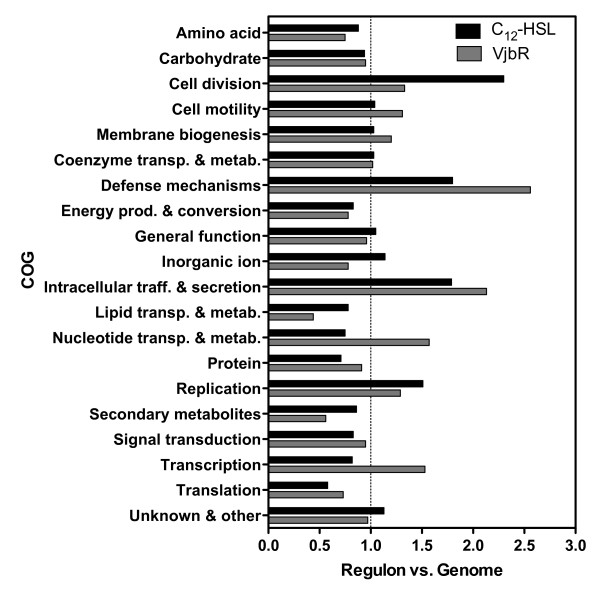
**COG functional categories found to be over and under represented by the deletion of *vjbR *and the addition of C_12_-HSL to wildtype cells, indicated by microarray analyses**. Ratios were calculated by comparing the proportion of genes found to be altered by the putative QS component to the total number of genes classified in each COG category present in the *B. melitensis *genome.

The differentially expressed genes were categorized by clusters of orthologous genes (COGs), obtained from the DOE Joint Genome Institute Integrated Microbial Genomics project http://img.jgi.doe.gov/cgi-bin/pub/main.cgi. This classification revealed categories that were equally altered by both the *vjbR *mutant and addition of C_12_-HSL to wildtype bacteria (Fig. 3). For example; defense mechanisms, intracellular trafficking and secretion were highly over-represented when compared to genomic content. Of particular note, genes involved in cell division were found to be over-represented in wildtype bacteria grown in the presence of C_12_-HSL but not by deletion of *vjbR*, indicating that C_12_-HSL regulates cellular division and may play a key role in the intracellular replication of the bacteria.

Genes found to be altered by deletion of *vjbR *and treatment with C_12_-HSL in both wildtype and Δ*vjbR *backgrounds were compared to data compiled from random mutagenesis screenings, resulting in the identification of 61 genes (Tables 2, 3, 4 and Additional File 3, Table S3) [22,28,39]. This correlation strongly suggests that VjbR and C_12_-HSL are involved not only in regulating the expression of a diverse array of genes but numerous genetic loci that individually make significant contributions to the intracellular survival of *Brucella *spp.

**Table 3:** Transcripts associated with transport significantly altered between 16M and 16MΔ*vjbR*, with and without the treatment of C_12_-HSL to cells.

BME Loci	Gene Function	Exponential Growth Phase Change fold			Stationary Growth Phase Change (fold)			STM
			
		Δ*vjbR*/wt	wt+AHL/wt	Δ*vjbR*/Δ*vjbR*+AHL	Δ*vjbR*/wt	wt+AHL/wt	Δ*vjbR*/Δ*vjbR*+AHL	
**Amino Acid**								
I 0114	ABC-Type AA Transport	1.6	2.1	-	1.8	1.5^⁪^	-	
I 0263	ABC-Type Leucine/Isoleucine/Valine/Threonine/Alanine Transport	-1.8^†^	-	-	2.1	2.1	-	
II 0038	D-Serine, D-Alanine, Glycine Transporter	-	-1.5^†^	-	-1.6^†^	-1.8	-	Ficht, u.p.
II 0517	ABC-Type Branched Chain AA Transport System, AzlC	-1.8	-	-	-2.2	-1.7^†^	-	
II 0873	ABC-Type High Affinity Branched Chain AA Transport System, LivF	-2.0^†^	-2.3	-	-	-1.5^†^	-	
II 0909	Glutamate, γ-Aminobutyrate Antiporter	-	-	-	-2.1	-1.7	-	
I 0260	ABC-Type High-Affinity Branched Chain AA Transport, BraF	-	2.1	-	-1.5^†^	-	3.0^†^	
I 0642	Urea Transporter	-2.3^†^	-1.9	2.0^†^	-	-	-	
I 1022	ABC-Type Arginine, Ornithine Transporter	1.7^†^	2.8	2.2^†^	-	-	-	
I 1869	Homoserine Lactone Efflux Protein	-	-2.3	-3.1^†^	-1.5^†^	-	2.1^†^	
II 0070	ABC-Type Branched Chain AA Transport System	-	1.6^†^	-	-2.5	-1.8^†^	1.9	
II 0484	ABC-Type Spermidine/Putrescine Transport System	-2.3	-2.5	-	-	-2.0	-2.3^†^	
**Carbohydrate**								
I 1385	ABC-Type Lactose Transport System	-2.6^†^	-3.2	-	-	-	-	Ficht, u.p.
II 0115	ABC-Type G3P Transport System	-1.7^†^	-3.2	-	-	-	-	
II 0301	ABC-Type Ribose Transport System, RbsC	1.5^†^	-	-	-1.9	-	-	
II 1096	MFS Family, Putative Tartrate Transporter	1.7^†^	2.6	-	-	-	-	
I 0556	MFS Transporter ?-Ketoglutarate Permease	-2.4^†^	-2.5	-	-	-	-2.2^†^	
II 0300	ABC-Type Ribose Transport System, RbsA	-1.9	-1.8^†^	-	1.7	-	1.6^†^	[22]
II 0362	ABC-Type Xylose Transport System, XylH	-1.6^†^	-2.5	-3.0^†^	-	-	-	
II 0700	Galactoside Transport System, MglC	1.6^†^	-	-1.8^†^	-2.1	-	5.5^†^	
II 0701	ABC-Type Ribose Transport System, RbsC	2.4^†^	2.2	-	-	-	2.6^†^	[33]
II 0702	ABC-Type Simple Sugar Transport System	1.5^†^	-	-3.6^†^	-	-2.8	-5.1^†^	
II 0838	Succinoglycan Biosynthesis Transport Protein, ExoT	-2.0	-4.3	-4.2^†^	-	-1.7	-	
II 0851	Exopolysaccharide Export, ExoF Precursor	-2.1	-	2.1^†^	-	-	-	
**Defense Mechanism**								
I 0361	ABC-Type Antimicrobial Peptide Transporter System, FtsX	-1.9	-	-	-	-1.6^†^	-	
I 0472	ABC-Type Multidrug Transport System	-	2.0	-	-1.6^†^	-1.5^†^	-	
I 0656	ABC-Type Multidrug Transporter	1.7	2.3^†^	-	1.6^†^	-	-	
I 1743	ABC-Type Multidrug Transporter System	-	-	-	-1.8^†^	-1.7	-	
I 1934	ABC-Type Oligopeptide Transport System	-1.6^†^	-1.9	-	-	-	-	
II 0199	ABC-Type Oligopeptide Transport System, OppF	-1.5^†^	-2.8	-	-	-	-	
II 0205	ABC-Type Oligo/Dipeptide Transport System, DppF	-1.9	-2.1^†^	-	1.6	-	-	
II 0285	ABC-Type Oligo/Dipeptide/Nickel Transport System, DppB	-	-	-	1.7	1.6^†^	-	[31]
II 0473	Cation/Multidrug Efflux Pump	-1.8	-1.5^†^	-	1.8	-	-	
II 0801	ABC-Type Multidrug Transport System	-2.3	-	-	-1.7	-	-	
I 0187	DME Family Transporter	-	-	-3.9^†^	-1.8	-2.2	-	Ficht, u.p.
I 0654	ABC-Type Multidrug Transporter	-1.7	-2.1	-2.3^†^	2.0	-	-	
I 0655	ABC-Type Multidrug Transporter	-1.8	-2.3	-	-1.7^†^	-	1.5^†^	
I 0984	ABC-Type β -(1,2) Glucan Transporter	-2.1	-	1.7^†^	-	-1.5^†^	-	
II 0221	ABC-Type Oligo/Dipeptide/Nickel Transport System, DppC	-	-1.9	-2.8^†^	-1.5^†^	-	-	
II 0382	Acriflavin Resistance Protein D	-1.5^†^	-	-	-1.8	-	1.8^†^	
**Inorganic Ions**								
I 1041	ABC-Type Fe-S Cluster Assembly Transporter	1.5^†^	2.0	-	-	-	-	
I 1954	ABC-Type Metal Ion Transport System	-2.0	-1.6	-	2.0	2.1	-	
II 0005	ABC-Type Molybdate-Binding Protein	-2.7	-2.4	-	1.8^†^	-	-	
II 0418	Mg^2+ ^Transporter Protein, MgtE	-3.2	-1.9^†^	-	-1.6^†^	-1.8^†^	-	
II 0798	ABC-Type Nitrate Transport System, NrtC	-	-	-	-2.1	-2.1	-	
II 0923	ABC-Type Spermidine/Putrescine Transport System	-1.9^†^	-2.6	-	-	-	-	[22]
II 1121	ABC-Type Fe^3+ ^Transport System, SfuB	-	-	-	-1.8^†^	-1.9	-	
I 0637	ABC-Type Cobalt Transport Protein, CbiQ	1.5^†^	2.3	1.9^†^	-1.6^†^	-	1.9^†^	
I 0641	ABC-Type Co^2+ ^Transport System	1.8^†^	1.9	-	-1.8	-	1.6^†^	
I 0659	ABC-Type Fe^3+ ^Siderophore Transport System	-1.8	-2.0	-	-	-	1.7^†^	
I 1739	ABC-Type Nitrate/Sulfonate/Bicarbonate Transporter	-1.5^†^	-1.8	-1.8^†^	-1.7	-2.1	-	
II 0176	ABC-Type High-Affinity Zn Transport System, ZnuB	-2.4^†^	-2.3	-1.8^†^	-	-	-	
II 0770	Potassium Efflux System, PhaA, PhaB	-2.0^†^	-2.1	-1.6^†^	-	-	-	
**Other**								
I 1852	ABC-Type Heme Exporter Protein B	-1.8	-1.9	-	-	-	-	
I 1860	ABC-Type Transporter, Lysophospholipase L1	-1.8^†^	-1.9	-	-	-	-	
I 1198	RDD Family, Hypothetical Membrane Spanning Protein	1.5	1.6^†^	-1.7^†^	-	-	-	
I 1554	MFS Family Transporter	-	-	-	-2.3	-2.0	2.0^†^	
I 1851	ABC-Type Heme Exporter Protein C	-	-1.9^†^	-1.6^†^	1.8	-	-	
II 1136	ABC-Type Uncharacterized Transport System	-1.5^†^	-1.9	-2.2	-	-	-	

A (-) indicates genes excluded for technical reasons or had a fold change of less than 1.5; ^† ^genes that did not pass the statistical significance test but showed an average alteration of at least 1.5-fold. Fold change values are the averaged log_2 _ratio of normalized signal values from two independent statistical analyses. Abbreviations are as follows: STM, Signature Tagged Mutagenesis; DME, Drug/Metabolite Exporter; G3P, Glycerol-3-Phosphate; AA, amino acid.

**Table 4:** Genetic loci transcripts significantly altered between 16M and 16MΔ*vjbR*, with or without the treatment of C_12_-HSL that may contribute to virulence.

BME Loci	Gene Function	Exponential Growth Phase Change (fold)			Stationary Growth Phase Change (fold)			STM
			
		Δ*vjbR*/wt	wt + AHL/wt	Δ*vjbR*/Δ*vjbR*+ AHL	Δ*vjbR*/wt	wt + AHL/wt	Δ*vjbR*/Δ*vjbR*+ AHL	
**Cell Membrane**								
I 1873	Autotransporter Adhesin	-2.2	-	-	-	-	-	
II 1069	Adhesin, AidA	-1.5^†^	-	-	-1.5	-	-	
I 0402	31 KDa OMP Precursor	-	1.5^†^	-	-1.7	-1.7^†^	-	
I 0330	OpgC Protein	-	-2.0	-1.9^†^	-	-	-	
I 0671	Integral Membrane Protein, Hemolysin	-	-2.7	-2.2^†^	-	-	-	[28]
II 1070	Adhesin AidA-I	1.7	-	-	-	-	-1.9^†^	
I 1304	Porin, F Precursor	-	-	-3.6^†^	-3.5	-2.0	-2.6^†^	
I 1305	Porin	-	-2.3	-1.8^†^	-1.5^†^	-	-	
**Cell Motility**								
II 0151	Flagellar M-Ring Protein, FliF	-2.2	-2.1	-	2.1^†^	-	-	[22,34]
II 0161	Flagellar Hook-Associated Protein 3	-1.8^†^	-2.7	-	-	-	-	
II 0165	Flagellar Biosynthesis Protein	-1.9^†^	-2.8	-	-	-	-	
I 1692	Flagellar Protein, FlgJ	-	-	-2.3^†^	-1.8	-2.1	-3.4^†^	
II 0160	Flagellar Hook-Associated Protein, FlgK	-1.6^†^	-2.0	-1.7^†^	-	-	-	
II 0162	FlaF Protein	-2.1	-2.0^†^	-	-	-	-1.6^†^	
II 0167	Flagellar Biosynthesis Protein, FlhA	-1.6^†^	-2.3	-1.8^†^	-1.5^†^	-1.9^†^	-5.5^†^	
II 1109	Chemotaxis Protein, MotA	-1.6^†^	2.0^†^	-3.6^†^	-1.7	-1.5^†^	-	
**Protease and Lipoprotein**								
I 0611	HflC Protein, Stomatin, Prohibitin, Flotillin, HflK-C Domains	-1.6	-	-	-	-1.7^†^	-	
I 1079	Lipoprotein NlpD	-	-1.5^†^	-1.6^†^	-1.6^†^	-1.9	-	
I 1799	Lipoprotein Signal Peptidase	2.2	2.1^†^	-	-	-1.6^†^	-	
II 0831	Hypothetical Protein, Aminopeptidase-Like Domain	-1.6^†^	-2.0	-	-2.3	-	3.1^†^	
I 0213	Metalloendopeptidase	-1.7^†^	-2.7^†^	-1.6^†^	2.1	-	-	
I 0282	Zinc Metalloprotease	-1.8	-1.7	-	-	-	3.4^†^	
II 0149	Extracellular Serine Protease	-3.2	-1.8	2.9^†^	-	-1.7	-	
**Secretion System**								
I 0390	VceA	-1.4^†^	-1.3^†^	-	-	-1.2^†^	-	
I 0948	VceC	1.1^†^	1.4^†^	-	1.6^†^	1.3^†^	-	
I 1094	Exopolysaccharide Production Negative Regulator Precursor, Tetratricopeptide Repeat	-	-	-	2.1	1.5^†^	-	
I 1141	Predicted Exported Protein	-1.6	-1.7	-	-	-	-	
I 1531	Tetratricopeptide Repeat Family Protein	-2.1	-2.4	-	-1.7	-	-	[34]
I 1077	Hypothetical Exported Protein, YajC	-1.5	-2.1	-	-1.8^†^	-1.5^†^	1.8^†^	
II 0025	Attachment Mediating Protein VirB1	-2.2	-1.9	-	-2.6	-2.2	-	[29,31,36]
II 0026	Attachment Mediating Protein VirB2	-	-2.1	-	-4.3	-3.6	-1.3^†^	[29,31,36]
II 0027	Channel Protein VirB3	-	-	-	-3.9	-3.2	-	[29-31,36]
II 0029	Attachment Mediating Protein VirB5	-2.0	-	1.6^†^	-5.7	-4.5	-1.2^†^	[29-32]
II 0030	Channel Protein VirB6	-	-	-1.7^†^	-2.8	-2.3	-	[29-31,36]
II 0032	Channel Protein VirB8	-1.6^†^	-	1.1^†^	-3.3	-2.6	-	[29,31,32,36]
II 0033	Channel Protein VirB9	-	-	-	-1.8	-1.9	-	[29,31,36]
II 0034	Channel Protein VirB10	-	-1.5	-	-2.0	-1.9	-	[29,31,36]
II 0036	OMP, OprF, VirB12	-	-	-	-1.7	-1.7	-	[29,36]
II 0466	Tetratricopeptide Repeat Family Protein	-	2.3	2.2^†^	-1.5^†^	-	-	
**Signal Transduction**								
II 0011	Transcriptional Regulatory Protein, HydG	-1.5^†^	-2.0	-	-	-	-	[31]
II 1014	Two Component Response Regulator	-	1.7^†^	-	1.6	-1.5^†^	-	
I 0370	Sensory Transduction Histidine Kinase	-1.7	-2.1	-2.2^†^	-1.6^†^	-	2.1^†^	
I 0372	Two-Component Response Regulator	1.6^†^	-	-1.5^†^	1.5^†^	1.8	-	
I 2034	Sensor Protein, ChvG	-	-1.7	-2.4^†^	-2.0	-1.6	-	
**Stress Response**								
I 0887	Peptidyl-Prolyl Cis-Trans Isomerase	-	-1.7	-	1.7	1.6	-	
I 1619	Hsp33-Like Chaperonin	-	-	-	1.8	1.6^†^	-	
II 0245	Universal Stress Protein Family, UspA	-1.8	-1.7	-2.0^†^	-2.5	-2.5	-	

A (-) indicates genes excluded for technical reasons or had a fold change of less than 1.5; ^† ^genes that did not pass the statistical significance test but showed an average alteration of at least 1.5-fold. Fold change values are the averaged log_2 _ratio of normalized signal values from two independent statistical analyses. Abbreviations are as follows: STM, Signature Tagged Mutagenesis; OMP, Outer Membrane Protein.

### VjbR and C_12_-HSL modulate gene transcription in a temporal manner

Comparison of altered gene transcripts resulting from the Δ*vjbR *mutation revealed that 13% (54 statistically significant genes) were found to be regulated at both growth phases, suggesting that VjbR exerts temporal control over gene regulation (Additional File 3, Table S3). A similar subset of genes were also identified in wildtype bacteria that were treated with C_12_-HSL when compared to those without treatment, with 12% (54 genes, Additional File 3, Table S3) of transcripts altered at both growth stages. The low correlation of genes altered at both growth stages suggests that both VjbR and C_12_-HSL regulate distinct regulons at the two growth stages examined.

A recent study compared microarray and proteomic data from a Δ*vjbR *mutant at a late exponential growth phase (OD_600 _= 0.75), corresponding to a total of 14 genes and the *virB *operon found at the growth phases examined here [23]. Of the 14 genes in common with the study by Uzureau et al.; 2 genes and the *virB *operon identified in our study (BMEI1435 and I1939) correlated in the magnitude of change with both the protein and microarray data, BMEI1267 correlated with the protein data, and 3 genes (BMEI1900, II0358 and II0374) correlated with the microarray data (Additional File 3, Table S3) [23]. Additionally, 5 genes did not correspond with the magnitude of alteration in the microarray analyses conducted in this study (BMEI0747, I1305, I1367, II0098 and II0923; Table 3 and Additional File 3, Table S3) [23]. The low similarity of regulated genes from these two studies that examined a total of 3 different growth phases provides further support of the VjbR temporal gene regulation observed here [23].

**Figure 4:**
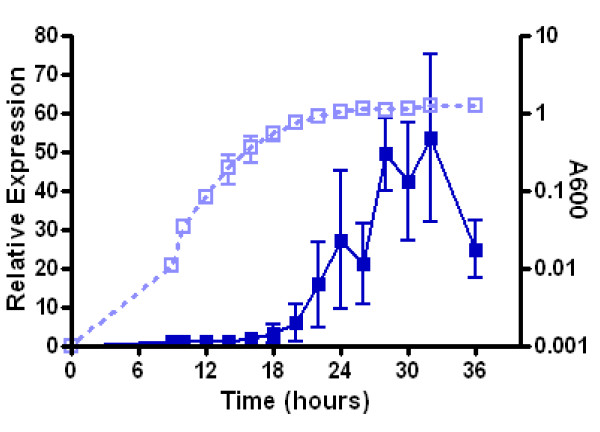
**Relative expression of *vjbR *transcript over time. Taqman real-time RT-PCR of *vjbR *in *B. melitensis *16M expressed as the mean relative concentration (to 16srRNA) from 3 biological replicates ± standard deviation**. Filled blue squares represent the relative expression of *vjbR *and the open light blue squares represent the OD_600 _of corresponding cultures. The exponential growth stage for microarray analysis corresponds to OD_600 _= 0.4 (14 hrs) and the stationary growth phase corresponds to OD_600 _= 1.5 (28 hrs).

A similar pattern of temporal gene regulation by AHL quorum sensing signals has also been observed in *P. aeruginosa *[26,40]. Distinct regulons were identified at an exponential and early stationary growth phase by utilization of a mutated strain that does not produce AHL signals, leading to the conclusion that the temporal regulation is independent of AHL concentration [26,40]. Examination of two *luxR *gene transcript levels in *P. aeruginosa *revealed an increase from the late logarithmic to early stationary phase, coinciding with the induction of most quorum-activated genes and supporting a hypothesis that the receptor levels govern the onset of induction [40]. Likewise, the relative expression of *B. melitensis vjbR *was found to increase 25-fold from exponential to stationary growth phase by qRT-PCR (Fig. 4). The observed increase in the transcript levels of *vjbR *supports a similar hypothesis for the temporal gene regulation observed by VjbR in *B. melitensis*

### VjbR and C_12_-HSL alter expression of a common set of genes

To examine the relationship between VjbR and C_12_-HSL gene regulation, the significantly altered genes from the VjbR regulon were compared to the significantly altered genes from the C_12_-HSL regulon (Tables 2, 3, 4 and Additional File 3, Table S3). In all, 72 genes were found to be co-regulated during the exponential growth phase and 55 genes at the stationary growth phase, representing approximately 20% of the total number of altered genes identified by microarray analysis. The majority of the common, differently expressed transcripts (124 out of 127) were found to be altered in the same direction by both the *vjbR *mutant and in response to C_12_-HSL administration, implying that VjbR and C_12_-HSL exert inverse effects on gene expression.

In addition to the T4SS and flagella operons being inversely co-regulated, T4SS-dependent effector proteins VceA and VceC were also found to be inversely regulated by the *vjbR *deletion mutant and addition of C_12_-HSL to wildtype cells, as well as exopolysaccharide production, proteases, peptidases and a universal stress protein (Table 4). Flagellar and exopolysaccharide synthesis genes have been implicated in the intracellular survival of *Brucella *in mice and macrophages [4,41]. The down-regulation of these factors in *vjbR *mutants and in response to C_12_-HSL suggests that VjbR promotes *Brucella *virulence; while conversely, C_12_-HSL represses such gene expression, either through the same regulatory pathway or independently.

These results expand on earlier findings that C_12_-HSL represses transcription of the T4SS through interactions with the response domain of VjbR [17,42]. The genes identified as co-regulated between VjbR and C_12_-HSL may be the result of C_12_-HSL reducing VjbR transcriptional activity through the AHL binding domain. Additionally, the observation that the expression of *vjbR *itself was down-regulated at the stationary growth phase in response to C_12_-HSL administration further supports a non-cooperative relationship between VjbR and C_12_-HSL, (2.9-fold by qRT-PCR and 1.2-fold by microarray analysis, Table 1).

### Physiological characterization of VjbR and C_12_-HSL transcriptomes

*Virulence*. Microarray results confirmed alteration of the previously identified T4SS and flagellar genes, both virulence-associated operons found to be regulated by VjbR and/or C_12_-HSL, as well as genes with homology to the recently identified T4SS effector proteins in *B. abortus *and *B. suis *[14,27]. Furthermore, many putative virulence factors not previously correlated with VjbR or C_12_-HSL regulation in *Brucella *spp. were identified; including protein secretion factors, adhesins, lipoproteins, proteases, outer membrane proteins, antibiotic and toxin resistance genes, stress survival genes and genes containing tetratricopeptide repeats (Tables 2, 3, 4 and Additional File 3, Table S3). Many of these gene products have been found to be associated with virulence and infection in numerous other bacterial pathogens have not been studied in *Brucella *spp., calling for further investigation and characterization.

A BLAST search of the T4SS effector protein VceA against *B. melitensis *16M revealed two genes with high and low degrees of similarity, BMEI0390 and BMEII1013, with 98.8% and 35% (respectively) amino acid similarity. *VceA *(BMEI0390) was found to be down-regulated at the exponential growth phase by the *vjbR *deletion mutant and the addition of C_12_-HSL (1.4-fold and 1.3 fold) but was not statistically significant nor met the cut-off value of 1.5-fold (Table 4). Additionally, a BLAST search of VceC revealed a gene with 99% amino acid similarity, BMEI0948, which was found to be up-regulated by Δ*vjbR *and treatment of C_12_-HSL in wildtype cells at the stationary growth phase (1.6 and 1.3-fold, respectively, Table 4). The *vceC *homologue, which is located downstream of a confirmed VjbR promoter sequence, was unexpectedly found to be down-regulated by VjbR and not up-regulated along with the T4SS (*virB *operon) [27]. Expression of *vceA *was found to be promoted at the exponential growth phase by VjbR, however, no information was obtained at the stationary growth phase for comparison to *virB *in this global survey.

Deletion of *vjbR *resulted in the down-regulation of a gene locus that encodes for the ATP-binding protein associated with the cyclic β-(1,2) glucan export apparatus (BMEI0984, 2.1-fold) and an exopolysaccharide export gene *exoF *(BMEII0851, 2.1-fold) at the exponential growth phase; while the treatment of C_12_-HSL in the Δ*vjbR *null background up-regulated these same genes 1.7 and 2.1-fold, respectively, (Table 3). Additionally, C_12_-HSL was found to down-regulate expression of *opgC *(BMEI0330), responsible for substitutions to cyclic β-(1,2) glucan, 2.0 and 1.9-fold at the exponential growth phase in the wildtype and Δ*vjbR *backgrounds (respectively, Table 4) [43]. Cyclic β-(1,2) glucan is crucial for the intracellular trafficking of *Brucella *by diverting the endosome vacuole from the endosomal pathway, thus preventing lysosomal fusion and degradation and favoring development of the brucellosome [4]. Mutations in the *vjbR *locus do not appear to have a profound effect on trafficking diversion from the early endosomal pathway; however, it is plausible that cyclic β-(1,2) glucan and derivatives may be important for subsequent vacuole modulation and/or brucellosome maintenance during the course of infection [14].

Deletion of *vjbR *resulted in alteration in the expression of three adhesins: *aidA *(BMEII1069, down-regulated 1.5-fold at both growth stages examined), *aidA*-1 (BMEII1070, up-regulated 1.7-fold) at the exponential growth phase, and a gene coding for a cell surface protein (BMEI1873, down-regulated 2.2-fold) at the exponential growth phase (Table 4). Adhesins can serve as potent biological effectors of inflammation, apoptosis and cell recognition, potentially contributing to the virulence and intracellular survival of *Brucella *spp. [44-46]. For instance, AidA adhesins are important for *Bordetella pertussis *recognition of host cells and in discriminating between macrophages and ciliated epithelial cells in humans [45].

*Transporters*. A large number of genes encoding transporters (90 total) were altered in Δ*vjbR *or in response to the addition of C_12_-HSL to wildtype cultures (Table 3 and Additional File 3, Table S3). For example, an exporter of O-antigen (BMEII0838) was identified to be down-regulated 2.0-fold by the deletion of *vjbR *at an exponential growth phase, and 4.3 and 1.7-fold by the addition of C_12_-HSL to wildtype cells at exponential and stationary growth phases, respectively (Table 3). Among the differently expressed transporters, ABC-type transporters were most highly represented, accounting for 62 out of the 90 transporter genes (including 15 amino acid transporters, 10 carbohydrate transporters and 16 transporters associated with virulence and/or defense mechanisms) (Table 3 and Additional File 3, Table S3). The correlation between ABC transporters and the ability to adapt to different environments is in tune with the ability of *Brucella *spp. to survive in both extracellular and intracellular environments [47].

*Transcription*. Based on microarray analysis results, *vjbR *deletion or the addition of C_12_-HSL to wildtype cells altered the expression of 42 transcriptional regulators, comprised of 12 families and 14 two-component response regulators or signal transducing mechanisms (Table 2 and Additional File 3, Table S3). Among the transcriptional families altered by Δ*vjbR *and/or the addition of C_12_-HSL, 9 families (LysR, TetR, IclR, AraC, DeoR, GntR, ArsR, MarR and Crp) have been implicated in the regulation of virulence genes in a number of other pathogenic organisms [35,48-55]. The regulation of *virB *has been reported to be influenced not only by the deletion of *vjbR *and C_12_-HSL treatment, but by several additional factors including integration host factor (IHF), BlxR, a stringent response mediator Rsh, HutC, and AraC (BMEII1098) [14,15,56-58]. The same AraC transcriptional regulator was found to altered by *vjbR *deletion and C_12_-HSL treatment of wildtype cells: down-regulated 1.8 and 2.8-fold at exponential phase (respectively), and up-regulated 1.9 and 1.5-fold (respectively) at the stationary growth phase (Table 2). Additionally, HutC (BMEII0370) was also found to be down-regulated at the exponential growth phase by the Δ*vjbR *mutant (1.8-fold), suggesting several levels of regulation for the *virB *operon by the putative QS components in *B. melitensis *(Additional File 3, Table S3).

In addition to transcriptional regulators linked to virulence, microarray analyses also revealed two differentially expressed transcriptional regulators that contain the LuxR HTH DNA binding pfam domain (gerE, pfam00196). Gene transcript BMEII0051 was found to be down-regulated 1.9 and 2.8-fold in response to a *vjbR *deletion and addition of C_12_-HSL to wildtype cells (respectively) at an exponential growth phase (Table 2). This *luxR*-like gene is located downstream of a VjbR consensus promoter sequence and thus most likely directly promoted by VjbR [27]. The second *luxR*-like gene, BMEI1607, was up-regulated 1.8-fold and 3.0-fold in the *vjbR *mutant and in response to exogenous C_12_-HSL at the exponential growth phase (respectively), and down-regulated 1.5-fold by the deletion of *vjbR *at the stationary growth phase (Table 2). This gene locus was not found to be located downstream of a predicted VjbR promoter sequence and may or may not be directly regulated by VjbR. Additionally, *blxR *was found to be induced 27.5-fold in wildtype cells treated with C_12_-HSL at the stationary growth phase by qRT-PCR (Table 1). Likewise, qRT-PCR verified a 2.9-fold down-regulation of *vjbR *in wildtype cells supplied with exogenous C_12_-HSL at the stationary growth phase. The identification and alteration of genes containing the HTH LuxR DNA binding domain by Δ*vjbR *and C_12_-HSL administration, particularly one located downstream of the VjbR consensus promoter sequence, is of great interest. These observations potentially suggest a hierarchical arrangement of multiple transcriptional circuits which may or may not function in a QS manner, as observed in organisms such as *P. aeruginosa *[26].

*AHL synthesis*. The deletion of *vjbR *or addition of C_12_-HSL resulted in alteration in the expression of 15 candidate AHL synthesis genes, based on the gene product's potential to interact with the known metabolic precursors of AHLs, S-adenosyl-L-methionine (SAM) and acylated acyl carrier protein (acyl-ACP) (Additional File 2, Table S2) [59]. An *E. coli *expression system was utilized because *B. melitensis *has been shown to produce an AiiD-like lactonase capable of inactivating C_12_-HSL [60]. Cross streaks with *E. coli *AHL sensor strains and clones expressing candidate AHL synthesis genes failed to induce the sensor stains, while positive control *E. coli *clones expressing *rhlI *and *lasI *from *P. aeruginosa *and exogenous 3-oxo-C_12_-HSL did in fact induce the sensor strains (data not shown) [61].

### C_12_-HSL regulates gene expression independent of VjbR

In addition to the investigation on the influences of a *vjbR *deletion or addition of C_12_-HSL to wildtype bacteria on gene expression, treatment of Δ*vjbR *with exogenous C_12_-HSL was also assessed by microarray analyses. Compared to untreated wildtype cells, 87% fewer genes were identified as differentially altered in response to C_12_-HSL in the *vjbR *null background as opposed to wildtype cells administered C_12_-HSL. In the absence of VjbR, exogenous C_12_-HSL altered the expression of 82 genes; 34 at the exponential growth phase and 48 genes at the stationary growth phase (Fig. 2, red circles and Additional File 5, Table S5). Of these 82 statistically significant altered transcripts, only 4 were commonly altered with the same magnitude by a deletion of *vjbR *or wildtype cells treated with C_12_-HSL (Fig. 2). At the exponential growth phase, administration of C_12_-HSL exerted an equal effect on gene expression, up and down-regulating 19 and 23 genes (respectively, Fig. 2). On the contrary, at the stationary phase all 48 genes were up-regulated, a dramatically different profile than the down-regulation observed for the majority of differently expressed genes in C_12_-HSL treated wildtype cells (Fig. 2). Collectively, this data supports that C_12_-HSL is capable of influencing gene expression independent of VjbR.

There is evidence that C_12_-HSL may interact with a second LuxR homologue, BlxR [18]. Induction of *blxR *expression in response to C_12_-HSL was highly variable by microarray analysis; however, qRT-PCR revealed that *blxR *was up-regulated 99.5-fold in bacteria lacking *vjbR *treated with C_12_-HSL, compared to 27.5-fold in wildtype cells that were administered C_12_-HSL at the stationary growth phase. One possible explanation for this observation is that VjbR inhibits the induction of *blxR *by binding the AHL substrate and therefore lowering the cellular concentration of available C_12_-HSL for *blxR *induction, but has not been demonstrated.

Interestingly, 58% of the gene transcripts found to be altered in an recent study of the function of Δ*blxR *were also found to be altered by the addition of C_12_-HSL in the Δ*vjbR *background, and increased to 88% if we lowered the threshold from our 1.5-fold cutoff (Additional File 5, Table S5) [15]. A second study that similarly examined the transcript and proteomic alterations due to a deletion in *babR *corresponded with 6 genes identified in our study: with 2 genes found to be unique to the addition of C_12_-HSL in the Δ*vjbR *background (BMEI0231 and I1638, Additional File 5, Table S5), and 4 genes additionally altered by the deletion of *vjbR *or addition of C_12_-HSL in the wildtype background (BMEI0451, I0712, I1196 and II0358, Additional File 3, Table S3) [23]. Although many of these genes were not statistically significant in our analyses, this is a strikingly high correlation since the same conditions were not examined (Δ*blxR *vs. wt compared to Δ*vjbR *vs. Δ*vjbR *+ C_12_-HSL), as well as the use of differing microarray platforms and analyses procedures. This connection may suggest that the genes altered by the presence of C_12_-HSL in the absence of VjbR may be due to C_12_-HSL activation of BlxR.

## Conclusions

The goal of this work was to provide an elementary understanding in the role of the putative QS components in the virulence and survival of *B. melitensis*. VjbR is a homologue of LuxR, a transcriptional regulator previously shown to interact with an AHL signal C_12_-HSL and modulate expression of transcripts required for intracellular survival [14,17]. Custom *B. melitensis *microarrays were utilized to examine the regulons controlled by VjbR and C_12_-HSL, revealing a large number of genes potentially involved in the virulence and intracellular survival of the organism. Such genes include adhesins, proteases, lipoproteins, a hemolysin, secretion system components and effector proteins, as well as metabolic genes involved in energy production, amino acid, carbohydrate, and lipid metabolism. Furthermore, deletion of *vjbR *and C_12_-HSL treatment altered the expression of genes coding for components involved in the transport of numerous substrates across the cell membrane.

The microarray analyses conducted in this study also confirmed previous findings that *fliF *and the *virB *operon are regulated by Δ*vjbR *and exogenous C_12_-HSL treatment at an exponential growth phase and stationary growth phase (respectively), as well as the potential effector proteins VceA and VceC, validating the microarray approach to identify additional genes regulated by these putative QS components [14,27]. The contribution of VjbR gene regulation at different growth phases in not fully understood, but microarray analyses suggests that there are distinct sets of genes regulated at both growth phases in addition to the flagellar and T4SS operons. Previous studies examining the effect of timing on QS related genes in *P. aeruginosa *hypothesized that the transcriptional regulator and not the inducing or repressing signal is responsible for the continuum of responses observed [40]. Such a hypothesis is supported by the observed increase of *vjbR *expression over time in *B. melitensis*.

Deletion of *vjbR *and treatment of C_12_-HSL both resulted in a global modulation of gene expression. Examination of the relationship in respect to the genes commonly altered between Δ*vjbR *and wildtype bacteria administered C_12_-HSL suggests that C_12_-HSL reduces VjbR activity, based upon the following observations: 1) An inverse correlation in gene expression for all but three genes found to be altered by VjbR and C_12_-HSL, 2) Addition of exogenous C_12_-HSL to growth media mimics the deletion of VjbR in respect to gene alteration, 3) In the absence of *vjbR*, C_12_-HSL treatment has a markedly different effect on gene expression at the stationary growth phase, found to only promote gene expression, and 4) *virB *repression in response to the addition of C_12_-HSL is alleviated by deletion of the response receiver domain of VjbR [17]. The observed promotion of gene expression with the treatment of C_12_-HSL in a Δ*vjbR *background could potentially be occurring through a second LuxR-like protein BlxR, supported by the high correlation of commonly altered genes by Δ*blxR *and Δ*vjbR *with the addition of C_12_-HSL in independent studies [15,23]. Often, the LuxR transcriptional regulator and AHL signal form a positive feedback loop, increasing the expression of *luxR *and the AHL synthesis gene [62]. The observed up-regulation of *blxR *by C_12_-HSL may be an example of such a feedback loop, further supporting an activating role C_12_-HSL and BlxR activity.

Although evidence is indirect, these observations suggest that there may be two dueling transcriptional circuits with the LuxR transcriptional regulators (VjbR and BlxR). C_12_-HSL may provide a level of regulation between the two systems, deactivating VjbR and potentially activating BlxR activity during the transition to stationary phase. It appears that C_12_-HSL reduces VjbR activity, alters expression of 2 additional transcriptional regulators that contain the LuxR DNA binding domain, induces expression of BlxR and potentially activates gene expression through interactions with BlxR. It would be interesting to determine if the decrease in *virB *expression observed in wildtype cells at stationary phase is a result of C_12_-HSL accumulation and subsequent "switching" of transcriptional circuits *in vitro *[63]. Further experiments are needed to fully understand the temporal regulation of VjbR and associations with C_12_HSL, as well as indentification of AHL synthesis gene(s) in *Brucella *spp.

The role of the LuxR transcriptional regulators VjbR and BlxR and the AHL signal in relation to quorum sensing has not been fully deduced. Continuing investigation of these putative QS components *in vitro *and *in vivo *will help determine if these components work in a QS-dependent manner in the host cell or if they function more in a diffusion or spatial sensing context to allow differentiation between intracellular and extracellular environments [64]. Future experiments that elucidate how these processes contribute to the "stealthiness" of *Brucellae *and will provide additional clues to the intracellular lifestyle of this particular bacterium.

## Authors' contributions

JNW conceived, designed and performed the experiments, and drafted the manuscript. CLG performed computational analyses and assisted in drafting the manuscript. KLD performed computational analyses, contributed to manuscript development and critically revised the manuscript. HRG helped to analyze the data and critically revised the manuscript. LGA contributed to the data acquisition and critically revised the manuscript. TAF conceived and coordinated the study and helped to draft the manuscript. All authors read and approved the final manuscript.

## Supplementary Material

Additional file 1 **Table S1: Bacterial strains and plasmids**. Details, genotypes and references for the strains and plasmids used in this study. Click here for file

Additional file 2 **Table S2: PCR and Quantitative Real-Time PCR primers and probes**. Provides the sequences and linkers (if applicable) of all primers used for cloning, and the qRT-PCR probes and primers used in this study. Click here for file

Additional file 3 **Table S3: Additional genetic loci identified with significant alterations in transcript levels between *B. melitensis *16M and 16MΔ*vjbR *with and without the addition of C_12_-HSL**. Gene transcripts found to be altered by comparison of wild type and Δ*vjbR*, both with and without the treatment of C_12_-HSL at an exponential and stationary growth phase. Click here for file

Additional file 4 **Table S4: Promoter(s) sequences and potential operons of downstream genes found to be altered by the deletion of *vjbR *and/or treatment of C_12_-HSL**. Operons that are both found to be downstream of the predicted VjbR promoter sequence and altered by comparison of wild type and Δ*vjbR*, both with and without the addition of C_12_-HSL at exponential or stationary growth phases. Click here for file

Additional file 5 **Table S5: Genetic loci identified with significant alterations in transcript levels between *B. melitensis *16MΔ*vjbR *and 16MΔ*vjbR *with the addition of C_12_-HSL**. Altered gene transcripts uniquely identified by the treatment of C_12_-HSL to the *B. melitensis *16MΔ*vjbR *background. Click here for file
