# Supplementary material for: Brucella melitensis VjbR and C12-HSL regulons: contributions of the N-dodecanoyl homoserine lactone signaling molecule and LuxR homologue VjbR to gene expression
Source: BMC Microbiol. 2010 Jun 8;10:167. doi: 10.1186/1471-2180-10-167 (PMC2898763; doi:10.1186/1471-2180-10-167)
Supplement: Additional file 1 — Table S1: Bacterial strains and plasmids. Details, genotypes and references for the strains and plasmids used in this study. [file 1471-2180-10-167-S1.DOCX]

TABLE S1. Bacterial strains and plasmids used in this study.

| Strain | Genotype or description | Reference |
| --- | --- | --- |
| 16M | *Brucella melitensis* bv.1 strain obtained from ATCC and re-isolated from an aborted goat fetus by this lab | - |
| 16MΔ*vjbR* | 16M with BMEII1116 deleted | This study |
| *vjbR*::Tn*5*Km | 16M with Tn*5* insertion in BMEII1116 | This study |
| 16MΔ*blxR* | 16M with BMEI1758 deleted | This study |
| *blxR*::Km | 16M with KanR cassette inserted in BMEI1751 | This study |
| 16MΔI1751::Km | 16M with BMEII0853 deleted | This study |
| 16MΔI1751 | 16M with BMEI1751 deleted | This study |
| 16MΔII0853::Km | 16M with BMEII0853 deleted | This study |
| 16MΔI1582::Km | 16M with KanR cassette inserted in BMEI1582 | This study |
| 16MΔI1582 | 16M with BMEI1582 deleted | This study |
| β2155 | *E. coli* *thrB1004 pro thi strA hsdS lacZ∆M15* (F’ *lacZ∆M15 lacI*^q^ *traD36 proA*^+^ *proB*^+^) *∆dapA::erm* (Erm^r^) *pir*::RP4 [::*kan* (Km^r^) from SM10 | [1] |
| JLD271 | *E. coli* K12 ∆*lac*X74 *sdi*A271::Cam | [2] |
| DH5α^TM^-T1^R^ | F-φ80*lacZ*∆M15∆(*lac*ZYA-*arg*F) U169 *recA*1 *end*A1 *hsd*R17(r_k-_,m_k+_) *pho*A *sup*E44 *thi*-1 *gyr*A96 *rel*A1 *ton*A | Invitrogen |
| BL21Gold(DE3) | *E. coli B F- dcm+ Hte ompT hsdS(r_B_- m_B_-) gal λ (DE3) endA Tet^r^* | Stratagene |
| Plasmid | Description | Reference |
| pBa1143 | pFuse containing *virB2, virB3* and part of *virB4* | [3] |
| pBluescript KSII^+^ | f1 + origin, Ap^R,^ lacZ', P lac promoter, pUC origin | Stratagene |
| pKD4 | FLP/FRT, Km^R^ | [4] |
| pEX18Ap | *sacB*, Ap^R^ | [5] |
| pMR10Kan | Km^R^, NCBI Accession number AJ606312 | Bourniquel, A.A., u.p |
| pJW038 | pMR10Kan expressing BMEII 1116 | This study |
| pET11a | Ap^R^, *T7/lacO* promoter, RBS binding site, T7 terminator | Stratagene |
| pAL101 | *rhlR*^+^ *rhlI*::*luxCDABE*; Tet^r^ p15A origin | [2] |
| pAL102 | *rhlI*::*luxCDABE*; Ter^r^ p15A origin | [2] |
| pAL105 | *lasR^+^lasI::luxCDABE*; Tet^r^ p15A origin | [2] |
| pAL106 | *lasI*::*luxCDABE*; Tet^r^ p15A origin | [2] |

**References**

1. Dehio C, Meyer M: **Maintenance of broad-host-range incompatibility group P and group Q plasmids and transposition of Tn*5* in *Bartonella henselae* following conjugal plasmid transfer from *Escherichia coli***. *J Bacteriol* 1997, **179**(2):538-540.

2. Lindsay A, Ahmer BM: **Effect of *sdiA* on biosensors of *N*-Acylhomoserine lactones**. *J Bacteriol* 2005, **187**(14):5054-5058.

3. Baumler AJ, Heffron F: **Identification and sequence analysis of *lpfABCDE*, a putative fimbrial operon of *Salmonella* *typhimurium***. *J Bacteriol* 1995, **177**(8):2087-2097.

4. Hoang TT, Karkhoff-Schweizer RR, Kutchma AJ, Schweizer HP: **A broad-host-range Flp-FRT recombination system for site-specific excision of chromosomally-located DNA sequences: application for isolation of unmarked *Pseudomonas* *aeruginosa* mutants**. *Gene* 1998, **212**(1):77-86.

5. Schweizer HP, Hoang TT: **An improved system for gene replacement and *xylE* fusion analysis in *Pseudomonas* *aeruginosa***. *Gene* 1995, **158**(1):15-22.
