# Supplementary material for: Brucella melitensis VjbR and C12-HSL regulons: contributions of the N-dodecanoyl homoserine lactone signaling molecule and LuxR homologue VjbR to gene expression
Source: BMC Microbiol. 2010 Jun 8;10:167. doi: 10.1186/1471-2180-10-167 (PMC2898763; doi:10.1186/1471-2180-10-167)
Supplement: Additional file 2 — Table S2: PCR and Quantitative Real-Time PCR primers and probes. Provides the sequences and linkers (if applicable) of all primers used for cloning, and the qRT-PCR probes and primers used in this study. [file 1471-2180-10-167-S2.DOCX]

TABLE S2. PCR and Quantitative Real-Time PCR primers used in this study.

| Primer ID | Sequence 5’ to 3’ (5’ Addition) | | Product |
| --- | --- | --- | --- |
|  | Forward Primer | Reverse Primer |  |
| TAF204/05 | ggcgcgccacgtcttgagcgattgtgtagg (AscI) | ggcgcgccggacaacaagccagggatgtaac (AscI) | nptII |
| TAF561/62 | cagacggcgcgccactcattggaaatatccttggtga (*AscI*) | gctctagagcgattacctcctccatcgccatt (*Xba*I) | BMEII1116-5’ |
| TAF563/64 | gctctagagcgtcttcgaggatgtacaattggc (*Xba*I) | tgagtggcgcgccgtctgatcaacatggtcg (*AscI*) | BMEII1116-3’ |
| TAF565/66 | cagagggcgcgcccccagtagcgtttc (*AscI*) | gctctagagcgatgtggaggctgtggagcg (*Xba*I) | BMEI1758-5’ |
| TAF567/68 | gctctagagcgcttgacgacggtatcattcgc (*Xba*I) | ctgggggcgcgccctctgaccttttccagtgtcc (*AscI*) | BMEI1758-5’ |
| TAF569/70 | tattgggcgcgccattcattgtatcgccctctg (*AscI*) | gctctagagcattggtccagtccccctgtctc (*Xba*I) | BMEII0853-5’ |
| TAF571/72 | gctctagagccaaaagcattggcgaccctg (*Xba*I) | tgaatggcgcgcccaatagcttgatcaggccc (*AscI*) | BMEII0853-3’ |
| TAF577/78 | agtgaggcgcgccattcgtcatgcgacttccgg (*AscI*) | gctctagacatcaaggaagtgcggcgtc (*Xba*I) | BMEI1582-5’ |
| TAF579/78 | gctctagagcaggaagaagatggtgccg (*Xba*I) | cgaatggcgcgcctcactttagggcggttccg (*AscI*) | BMEI1582-3’ |
| TAF581/82 | gctctagagcacagtttgcggggtgac (*Xba*I) | gcaagggcgcgccgaaaattgaagaaatatttcag (*AscI*) | BMEI1751-3’ |
| TAF583/84 | ttttcggcgcgcccttgccccaccttctccg (*AscI*) | gctctagaggtccgcaaacccacaaaatc (*Xba*I) | BMEI1751-5’ |
| TAF588/89 | aatctagagcggaaggtttccgcgcgac (*Xba*I) | aatctagaggtttcatcactgaggatatttcc (*Xba*I) | BMEII1116 |
| TAF644/45 | ggatcctcacaccgccatcgacagcg (*Bam*HI) | ggatccatcgaattgctctctgaatcgc (*Bam*HI) | *P. aeruginosa rhlI* |
| TAF646/47 | ggatccatcgtacaaattggtcggcgcgaagagttcg (*Bam*HI) | ggatcctcatgaaaccgcccttcgctgttccacc (*Bam*HI) | *P. aeruginosa lasI* |
| TAF648/49 | ggatccttgcatccggcttgcgcgcgtcttatcc (*Bam*HI) | ggatcctcagattgcagcaaaacctctttcg (*Bam*HI) | BMEI1213 |
| TAF650/51 | ggatccgtgccatggcgtggcggaggc (*Bam*HI) | ggatccttaaacttttgagcgcgccgcgtggc (*Bam*HI) | BMEI0701 |
| TAF652/53 | ggatccgtgtctttatcgcaaacgcgacttctggc (*Bam*HI) | ggatcctcaggctgcgacgggaacaggtttcaggg (*Bam*HI) | BMEI1768 |
| TAF654/55 | ggatccgtggacgccgcttttgaaaccgatgc (*Bam*HI) | ggatccttattctaaaactggcaaggcttcg (*Bam*HI) | BMEI1289 |
| TAF656/57 | ggtaccttagacgccgcgtctgttgcccc (*Bam*HI) | ggatccatgaaacccggccccgaaatccg (*Bam*HI) | BMEI0093 |
| TAF658/59 | ggatccatggctgcgacttatagaaaagacg (*Bam*HI) | ggatccctactttgcctgtgccctgatgattttcagcg (*Bam*HI) | BMEI1869 |
| TAF660/61 | ggatccatggaacggcgcatgatccc (*Bam*HI) | ggatccttatttatccggcttttgaccataccagcg (*Bam*HI) | BMEI0712 |
| TAF662/63 | ggatccgtgagtcgcatggttgatcaaacg (*Bam*HI) | ggatcctcaccgccgtgcagtcactccg (*Bam*HI) | BMEI0852 |
| TAF664/65 | ggatccatgctccgccgccgaaatacgagc (*Bam*HI) | ggatccctaaaccgccaacgtgactgttaccg (*Bam*HI) | BMEI1093 |
| TAF666/67 | ggatccttgcttgaagggaacgcacgg (*Bam*HI) | ggatcctcacacagtagcgctcaccc (*Bam*HI) | BMEI2003 |
| TAF668/69 | atgacggatccatgggtgaattcgcggtcgtg (*Bam*HI) | atgacggatcctcacccgtcatttggccagttc (*Bam*HI) | BMEI0032 |
| TAF670/71 | atgacggatccatggcagaacagaaatcgagc (*Bam*HI) | atgacggatccttattgcgcgctgcagtccttggacaggc (*Bam*HI) | BMEI1956 |
| TAF672/73 | ggatccatgatcgatgaaaatcatccc (*Bam*HI) | ggatcctcagcgccggataaaagtcagataagcg (*Bam*HI) | BMEI1969 |
| TAF674/75 | ggatccttgcccactggcaaattgcgc (*Bam*HI) | ggatccttatttcgtctgtcgggg (*Bam*HI) | BMEII0678 |
| TAF676/77 | ggatccatggcgcagcatcatcatctgaaggtgatcg (*Bam*HI) | ggatccttagcgctcctccctaacgagtgccgg (*Bam*HI) | BMEII0828 |
| Quantitative Reverse-Transcriptase PCR Primers and Taqman® Probes | | | |
| BME Loci | Forward Primer | Reverse Primer | Roche Universal Library Probe |
| I 0155 | aacctatggctggttcttcg | tttgcggagcacaaaataga | 70 |
| I 0561 | gaagatgcttatggcgttcc | cgccatagtcggtttcaag | 137 |
| I 0831 | ggaaggcaagaaaaacgtca | agagattcggtgcaaagca | 70 |
| I 0984 | gacgcatgaggaggtcca | cttttggcgaggatgaagtc | 70 |
| I 1758 | cgaaatcctgcgttggac | ttgaggattgtcccgatgat | 155 |
| II 0025 | catccatcatcgcagtcg | ttgcgggttgtacggttt | 83 |
| II 0151 | ccggttatgagctgttcgac | cacgggtaatctcctgcataa | 45 |
| II 0753 | agatcaagctgccgctca | aattggcaacgaaggtcatc | 121 |
| II 0838 | gcatgtgcagttttcctatcg | cagggtcagaaatgtcacca | 45 |
| II 1069 | gaccggtgagaacggtgat | ttgaatgcgagcccagat | 138 |
| II 1116 | ttgcgggttgtacggttt | caaggaattgcgtacggtct | 39 |
