# Supplementary material for: Brucella melitensis VjbR and C12-HSL regulons: contributions of the N-dodecanoyl homoserine lactone signaling molecule and LuxR homologue VjbR to gene expression
Source: BMC Microbiol. 2010 Jun 8;10:167. doi: 10.1186/1471-2180-10-167 (PMC2898763; doi:10.1186/1471-2180-10-167)
Supplement: Additional file 3 — Table S3: Additional genetic loci identified with significant alterations in transcript levels between B. melitensis 16M and 16MΔvjbR with and without the addition of C12-HSL. Gene transcripts found to be altered by comparison of wild type and ΔvjbR, both with and without the treatment of C12-HSL at an exponential and stationary growth phase. [file 1471-2180-10-167-S3.DOCX]

TABLE S3. Additional genetic loci identified with significant alterations in transcript levels between *B. melitensis* 16M and 16MΔ*vjbR* with and without the addition of C_12_-HSL.

| BME Loci | Gene Function | Exponential Growth Phase  Change (fold) | | | Stationary Growth Phase  Change (fold) | | | STM |
| --- | --- | --- | --- | --- | --- | --- | --- | --- |
|  |  | ∆*vjbR*  / wt | wt+AHL / wt | ∆*vjbR*  / ∆*vjbR*+AHL | ∆*vjbR*  / wt | wt+AHL / wt | ∆*vjbR*  / ∆*vjbR*+AHL |  |
| **Amino Acid Transport and Metabolism** | | | | | | | | |
| I 0079 | Prephenate Dehydrogenase | -1.7 | - | - | - | - | - |  |
| I 0143 | Putative Threonine Efflux Protein | - | -2.0 | -2.1^⁪^ | - | - | - |  |
| I 0207 | γ-Glutamyl Kinase | - | - | - | 1.7 | - | - |  |
| I 0208 | γ-Glutamyl Phosphate Reductase | -1.8 | 1.8^⁪^ | 1.8^⁪^ | -1.9 | -1.9 | - |  |
| I 0256 | D-AA Dehydrogenase | - | 1.7 | 1.6^⁪^ | - | 1.8^⁪^ | - |  |
| I 0451 | 2-Isopropylmalate Synthase | - | - | - | 1.9 | 2.4 | -2.1^⁪^ | [1, 2] |
| I 0615 | SerB Protein | -1.8 | - | - | 1.7 | - | - | [1] |
| I 0617 | Acetolactate Synthase III Large Subunit | -1.7 | - | - | - | - | - | [1, 3] |
| I 0843 | Indole-3-Glycerol Phosphate Synthase | 1.8 | 1.8 | - | - | - | - |  |
| I 0844 | Anthranilate Phosphoribosyltransferase | - | 1.6 | - | - | - | - |  |
| I 0978 | Nitrogen Regulatory Protein P-II | 1.5^⁪^ | 2.2 | 2.7 | - | - | - |  |
| I 1171 | N-Acetyl-γ-Glutamyl-Phosphate Reductase | 1.8^⁪^ | 2.4 | - | - | - | 1.7^⁪^ |  |
| I 1209 | ABC-Type L-AA Transport System, AapM | - | -2.0 | -2.5^⁪^ | - | - | - |  |
| I 1213 | Cystathionine β-Lyase | 1.5^⁪^ | 1.7 | 1.6^⁪^ | - | - | 1.6^⁪^ |  |
| I 1217 | ApeA Protein | - | 1.9 | 3.0 | - | - | 1.9^⁪^ |  |
| I 1378 | L-Asparaginase II | - | - | - | 1.7 | - | - |  |
| I 1381 | Choline Dehydrogenase | - | -1.6 | - | - | -1.9 | - |  |
| I 1621 | Bifunctional N-Succinyldiaminopimelate-Aminotransferase, Aacetylornithine Transaminase Protein | - | - | - | 1.5^⁪^ | 1.8 | - |  |
| I 1683 | Zinc-Dependent Metallopeptidase | - | -1.8 | -1.5^⁪^ | - | - | - |  |
| I 1719 | Sarcosine Oxidase γ Subunit | -1.5^⁪^ | -1.8 | - | - | - | - |  |
| I 1722 | Sarcosine Oxidase β Subunit | -1.7^⁪^ | -2.0 | -1.7^⁪^ | - | - | - |  |
| I 1755 | Sulfate Adenylyltransferase Subunit 2 | -1.7^⁪^ | -1.9 | - | - | - | 1.7^⁪^ |  |
| I 1939 | D-3-Phosphoglycerate Dehydrogenase | - | 1.8^⁪^ | - | -1.7 | - | - |  |
| I 2043 | Imidazole Glycerol Phosphate Synthase Subunit, HisH Protein | - | -1.6^⁪^ | - | -1.6 | - | 1.8^⁪^ |  |
| I 2058 | Shikimate 5-Dehydrogenase | -2.1 | - | - | - | - | - |  |
| II 0098 | ABC-Type High-Affinity Branched Chain AA Transport System, LivF | - | - | - | 1.6 | - | - |  |
| II 0099 | ABC-Type High-Affinity Branched Chain AA Transport System, LivG | -1.6 | - | - | - | - | - |  |
| II 0101 | ABC-Type High Affinity Branched-Chain AA Transport System, LivM | - | -2.1 | -1.5^⁪^ | - | - | - |  |
| II 0193 | ABC-Type Spermidine/Putrescine Transport System, PotA | -1.9 | - | - | - | - | - |  |
| II 0201 | ABC-Type Oligopeptide Transport System, OppC | - | -1.9 | - | - | - | - |  |
| II 0209 | ABC-Type Oligo/Dipeptide/Nickel Transport System, DppB | - | -1.9 | -2.8^⁪^ | - | - | - |  |
| II 0273 | Acetylglutamate Kinase | - | 2.3 | - | - | - | - |  |
| II 0339 | Alcohol Dehydrogenase (Acceptor) | 1.5^⁪^ | 1.9^⁪^ | -1.5^⁪^ | 1.7 | 1.5^⁪^ | - |  |
| II 0348 | 4-Aminobutyrate Aminotransferase | - | - | - | 1.6 | - | - |  |
| II 0366 | N-Formylglutamate Deformylase | - | -1.9 | -3.1^⁪^ | - | - | - |  |
| II 0368 | Imidazolonepropionase | -2.0 | - | 2.1^⁪^ | - | - | 1.7^⁪^ |  |
| II 0396 | Arginase | - | -1.8 | -1.5^⁪^ | -1.6^⁪^ | - | 1.5^⁪^ |  |
| II 0407 | Aspartate-Semialdehyde Dehydrogenase | - | 1.5^⁪^ | - | -1.6 | - | - |  |
| II 0441 | Acetylornithine Aminotransferase | -1.5 | -1.5^⁪^ | -1.5^⁪^ | - | - | - |  |
| II 0548 | ABC-Type Glycine/Betaine/L-Proline Transport System, ProV | - | -1.9 | - | - | - | - |  |
| II 0567 | ABC-Type Fe^3+^ Transport System, SfuC | - | - | - | 1.9 | - | - |  |
| II 0571 | IolD Protein | -1.5^⁪^ | -1.8 | - | - | - | - |  |
| II 0583 | ABC-Type Fe^3+^ Transport System, SfuC | - | - | - | -1.8 | - | 3.1^⁪^ |  |
| II 0602 | N-Methylhydantoinase, 5-Oxoprolinase | - | -1.7 | - | -1.7^⁪^ | -2.0 | - |  |
| II 0632 | ABC-Type High-Affinity Branched Chain AA Transport System, LivH | -1.8 | - | - | - | - | 1.6^⁪^ |  |
| II 0908 | Glutaminase | - | -2.0 | 2.2^⁪^ | - | - | - |  |
| II 1133 | Ornithine Decarboxylase | - | - | - | -2.4 | -1.8 | - |  |
| **Carbohydrate Transport and Metabolism** | | | | | | | | |
| I 0070 | Aquaporin Z | -1.9 | -2.2 | - | 1.8 | 1.7 | - |  |
| I 0267 | Membrane Protein, MosC | 1.7 | 2.0 | - | - | - | - | [3] |
| I 0309 | Phosphoglycerate Kinase | - | - | - | 1.6 | - | - |  |
| I 0396 | Diohydroxyacetone Kinase | 2.0^⁪^ | 2.5 | 1.8^⁪^ | - | - | - |  |
| I 0398 | Deoxyribonucleoside Regulator, Dihydroxyacetone Kinase | - | 3.7 | 2.6^⁪^ | -1.5^⁪^ | - | - |  |
| I 0663 | ABC-Type D-Ribose-Binding Transport System | - | - | - | - | -2.2 | - |  |
| I 0665 | ABC-Type Sugar Transport System | -1.6 | - | - | - | - | 1.5^⁪^ |  |
| I 0667 | FucU Protein | - | -1.8 | - | - | - | - |  |
| I 1087 | β-Hexosaminidase A | -1.6 | -2.2 | - | - | -1.8^⁪^ | - |  |
| I 1387 | Xylose Isomerase | -1.9 | - | - | - | -1.7^⁪^ | - |  |
| I 1389 | IolE Protein | -1.6 | - | - | - | - | 1.6^⁪^ |  |
| I 1435 | Polysaccharide Deacetylase | -1.5^⁪^ | - | - | 1.7 | - | - |  |
| I 1773 | Putative Lactoylglutathione Lyase | 1.6 | - | -2.2^⁪^ | - | - | 1.5^⁪^ |  |
| II 0048 | Extragenic Suppressor Protein, SuhB | -3.2 | -2.7 | - | - | - | - |  |
| II 0106 | Xylose Transcriptional Repressor | - | - | - | 1.6 | - | - |  |
| II 0113 | ABC-Type SN-G3P Transport System, UgpA | - | -1.9 | - | - | -1.6^⁪^ | -2.2^⁪^ |  |
| II 0139 | Phosphotyrosyl Phosphatase Activator, PtpA | - | 2.8 | - | - | - | - |  |
| II 0181 | N-Acetylglucosamine Kinase | - | - | - | -2.0 | - | - |  |
| II 0189 | L-Fuculose Phosphate Aldolase | -1.7 | - | - | - | -1.7^⁪^ | - |  |
| II 0357 | 2-Dehydro-3-Deoxygalactonokinase | - | - | - | -1.5 | - | - |  |
| II 0358 | 2-Dehydro-3-Deoxyphosphogalactonate Aldolase, Keto-Hydroxyglutarate-Aldolase/ Keto-Deoxy-Phosphogluconate Aldolase | - | - | - | -1.7 | - | 2.1 |  |
| II 0422 | Fructose-1,6-Bisphosphatase | -2.4 | - | 1.8^⁪^ | - | - | - |  |
| II 0476 | Uronate Isomerase | -1.9 | - | - | -1.5^⁪^ | - | - |  |
| II 0478 | D-Mannonate Oxidoreductase | - | -1.9 | - | - | - | - |  |
| II 0502 | ABC-Type Transport System Sugar-Binding Protein | -2.5 | - | - | - | - | - |  |
| II 0568 | Myo-Inositol-1(Or 4)-Monophosphatase | - | - | 1.8^⁪^ | -1.5^⁪^ | -1.8 | - |  |
| II 0596 | MFS Family, Arabinose Efflux | - | -2.5 | -3.2^⁪^ | - | -1.6^⁪^ | -2.5^⁪^ |  |
| II 0724 | Endoglucanase H | -1.5^⁪^ | -1.8 | - | - | 1.7 | - |  |
| II 0821 | Multi-drug Resistance Protein | 2.0 | - | - | -1.6^⁪^ | -1.7 | 2.2^⁪^ |  |
| II 0850 | GDP-Fucose Synthetase | -2.8 | - | 2.4^⁪^ | - | -1.5^⁪^ | - |  |
| II 0939 | ThuA Protein | - | -2.0 | -2.8^⁪^ | - | - | - |  |
| II 0941 | ABC-Type Maltose/Maltodextrin Transport System, MalK | - | - | -4.2^⁪^ | -2.0 | - | 2.0^⁪^ |  |
| II 1092 | Hydroxypyruvate Isomerase | - | - | - | - | 1.8 | - |  |
| **Cell Cycle Control, Cell Division and Chromosome Partitioning** | | | | | | | | |
| I 0007 | Glucose Inhibited Division Protein A, GidA | - | - | - | -1.6^⁪^ | -1.8 | - |  |
| I 0313 | Hypothetical Cytosolic Protein, ZapA | -1.6 | - | - | - | -1.7 | - |  |
| I 0342 | Cell Cycle Protein, MesJ | 2.9 | 1.6^⁪^ | -4.3^⁪^ | - | - | - |  |
| I 0633 | Camphor Resistance Protein, CrcB | - | -2.8 | -3.2^⁪^ | - | - | -2.1^⁪^ |  |
| I 1174 | Chromosome Partitioning, ParA Family | - | 1.8 | - | 1.9 | 1.8 | -3.9^⁪^ |  |
| II 0093 | Replication Protein A | - | - | - | - | -1.8 | - |  |
| II 0469 | Integral Membrane Protein | 1.5^⁪^ | - | - | -1.5^⁪^ | -1.6 | - |  |
| II 0470 | Integral Membrane Protein, Chromosome Condensation | 1.6^⁪^ | - | - | -2.3 | -1.8^⁪^ | - |  |
| **Cell Motility** | | | | | | | | |
| II 0155 | Chemotaxis MotC Protein Precursor | -1.7 | - | - | - | - | - |  |
| II 0188 | Type III Effector Hrp-Dependent Outer Proteins Domain | - | - | - | 1.7 | - | - |  |
| **Cell Wall, Membrane and Envelope Biogenesis** | | | | | | | | |
| I 0204 | Acetyltransferase | -2.2 | - | - | - | - | -1.7^⁪^ |  |
| I 0240 | Predicted Membrane Protein, FusB and FusC | - | - | - | -1.5 | - | - |  |
| I 0359 | HlyD Family Secretion Protein | -1.6 | - | - | - | - | - | [4] |
| I 0363 | TonB Protein | -1.7 | - | - | - | 2.0^⁪^ | 2.4^⁪^ |  |
| I 0418 | Lic2B Protein | - | -1.7 | - | - | - | - |  |
| I 0499 | Soluble Lytic Murein Transglycosylase | - | 2.4^⁪^ | - | 2.0 | 1.9 | -3.0^⁪^ | [5] |
| I 0561 | Membrane-Bound Lytic Murein Transglycosylase B | 1.5^⁪^ | - | -3.7^⁪^ | -2.0 | -1.7 | - | Ficht, u.p. |
| I 0566 | Soluble Lytic Murein Transglycosylase | - | -1.8 | -1.8^⁪^ | - | - | 1.6^⁪^ |  |
| I 0581 | UDP-N-Acetylenolpyruvoyl-Glucosamine Reductase | - | - | -2.5^⁪^ | - | -2.0 | -4.4^⁪^ |  |
| I 0682 | Potassium Efflux System, KefA | -2.1 | - | - | - | - | - |  |
| I 0795 | Glutamate Racemase | 1.8 | 1.5 | - | - | - | - |  |
| I 0831 | UDP-3-O-[3-hydroxymyristoyl] Glucosamine N-Acyltransferase | 1.6^⁪^ | 2.3 | 1.5^⁪^ | - | - | - |  |
| I 0835 | Lipid-A-Disaccharide Synthase | - | -1.8 | - | - | - | 1.9^⁪^ |  |
| I 0913 | Penicillin-Binding Protein 6 | 1.5 | - | - | - | - | - |  |
| I 0991 | Rare Lipoprotein A | - | - | - | 2.6 | - | - |  |
| I 1037 | Glycosyltransferase | 1.6 | 1.9 | - | 2.7 | 2.0 | - |  |
| I 1056 | N-Acetylmuramoyl-L-Alanine Amidase | - | - | - | -2.0 | - | - | [3] |
| I 1177 | Putative Colanic Biosynthesis UDP-Glucose Lipid Carrier Transferase | - | - | - | 1.5 | - | -2.2^⁪^ |  |
| I 1356 | Peptidoglycan Binding Domain Protein | - | 1.5 | - | -1.6 | - | - |  |
| I 1393 | Mannosyltransferase C | - | -2.0 | - | - | - | - | [5, 6] |
| I 1417 | Perosamine Synthetase, WbkB | - | - | - | -1.6^⁪^ | -2.3 | - |  |
| I 1602 | Glycosyltransferase | 1.7^⁪^ | 2.9 | 4.0^⁪^ | - | - | - |  |
| I 1858 | Phosphinothricin N-Acetyltransferase | - | - | 1.6^⁪^ | - | -1.6 | - |  |
| I 1904 | 3-Deoxy-Manno-Octulosonate Cytidylyltransferase | -2.0 | - | 1.7^⁪^ | - | -1.7^⁪^ | - |  |
| I 1972 | Apolipoprotein N-Acyltransferase | - | -2.0 | -2.1^⁪^ | - | - | - | Ficht,u.p. |
| I 2052 | Membrane-Bound Lytic Murein Transglycosylase A | -1.5 | - | -2.3^⁪^ | - | - | - |  |
| II 0157 | Soluble Lytic Murein Transglycosylase | -1.9 | -2.1 | - | -1.5^⁪^ | -2.1 | - |  |
| II 0260 | GTP-Binding Protein LepA | - | -1.9 | -1.6^⁪^ | - | - | - | [5] |
| II 0374 | Alanine Racemase, Catabolic | -2.2 | - | 3.8^⁪^ | - | - | - |  |
| II 0376 | Heat Resistant Agglutinin 1 Precursor | - | 2.1 | - | - | - | - |  |
| II 0440 | dTDP-Glucose 4,6-Dehydratase | -1.6 | - | - | 2.0 | - | -4.7^⁪^ |  |
| II 0730 | UDP-Glucose 4-Epimerase | -2.1 | -2.4 | - | 1.8 | 1.6^⁪^ | - |  |
| II 0830 | dTDP-4-Dehydrorhamnose 3,5-Epimerase, dTDP-4-Dehydrorhamnose Reductase | -2.8 | -2.1 | - | - | - | - |  |
| II 0839 | Putative Undecaprenyl-Phosphate α-N-Acetylglucosaminyl-Transferase | - | -2.1 | -2.9^⁪^ | - | - | - |  |
| **Coenzyme Transport and Metabolism** | | | | | | | | |
| I 0050 | Cobaltochelatase Protein, CobT | - | 1.7^⁪^ | - | -1.6^⁪^ | -1.6^⁪^ | - |  |
| I 0177 | Proporphyrinogen-III Synthetase | -1.5^⁪^ | -2.2 | - | - | - | - |  |
| I 0221 | Pyridoxine Kinase | - | 1.7^⁪^ | - | 1.6 | 1.6 | - |  |
| I 0286 | Putative Nucleotide-Binding Protein | -1.8 | -2.5 | - | - | - | 2.3 |  |
| I 0315 | 5-Formyltetrahydrofolate Cyclo-Ligase | -2.0 | -2.0 | - | - | -1.6 | - |  |
| I 0660 | ABC-Type Cobalamin/Fe^3+^ Siderophore Transport System | - | - | -2.1^⁪^ | 1.6 | - | - |  |
| I 0700 | Precorrin-3B C17-Methyltransferase Protein, CbiG | - | -1.8^⁪^ | -1.9^⁪^ | 2.1 | - | - |  |
| I 0701 | Precorrin-4 C11-Methyltransferase | -1.6 | -1.9 | - | - | - | 1.8^⁪^ |  |
| I 0702 | Precorrin-6x Reductase | - | - | - | 2.5 | - | 1.9^⁪^ |  |
| I 0703 | Cobalt-Precorrin-6A Synthase Protein, CbiD | -1.7 | - | - | - | -2.1 | - |  |
| I 0707 | CbiB Protein | -1.5^⁪^ | -2.1 | -1.8^⁪^ | - | - | 1.5^⁪^ |  |
| I 0712 | Precorrin-3B C17-Methyltransferase Protein, CbiG | - | -2.0 | -2.3^⁪^ | - | - | 1.6^⁪^ |  |
| I 0713 | Precorrin-2 C20-Methyltransferase | - | -1.7 | - | - | - | 2.2 |  |
| I 0842 | Molybdenum Cofactor Biosynthesis Protein C | - | 1.5^⁪^ | - | 1.7 | 1.6 | - |  |
| I 0954 | 2-Amino-4-Hydroxy-6- Hydroxy-Methyldihydropteridine Pyrophosphokinase | -2.1 | -2.0 | - | - | - | - |  |
| I 1021 | Molybdopterin-Guanine Dinucleotide Biosynthesis Protein B | 1.7^⁪^ | 2.5^⁪^ | - | -1.5 | - | - |  |
| I 1293 | Coproporphyrinogen III Oxidase | -1.6^⁪^ | -2.1 | -1.9^⁪^ | - | - | - |  |
| I 1517 | Pyridoxamine 5’-Phosphate Oxidase | -2.1 | -1.8 | 2.2^⁪^ | - | -2.0 | - |  |
| I 1768 | Uroporphyrin-III C-Methyltransferase / Precorrin-2 Oxidase / Ferrochelatase | -2.1 | -3.0 | -1.6^⁪^ | 1.7 | -2.2 | -6.6^⁪^ |  |
| I 1771 | Coproporphyrinogen III Oxidase | - | - | - | 1.6 | - | - |  |
| I 1834 | Ubiquinone/Menaquinone Biosynthesis Methyltransferase, UbiE | 1.7^⁪^ | - | -2.0^⁪^ | -1.9 | -1.5^⁪^ | 2.3^⁪^ |  |
| I 2039 | Pantothenate Kinase | -2.1 | -1.9 | - | - | - | 1.9^⁪^ |  |
| II 0235 | Phosphopantothenoylcysteine Synthase/Decarboxylase | -1.6 | - | -1.9^⁪^ | 1.5 | - | - |  |
| II 0528 | Glutamate-Cysteine Ligase | -1.5^⁪^ | - | 1.6^⁪^ | - | -2.3 | - |  |
| II 0957 | VdcC Protein | -1.7 | -2.0 | - | - | - | - |  |
| **Defense Mechanisms** | | | | | | | | |
| I 0356 | Type 1 Capsular Polysaccharide Biosynthesis Protein J | - | - | -2.2^⁪^ | 1.6 | - | - |  |
| I 0506 | DME Family Transporter | - | - | - | 1.5 | - | 2.2^⁪^ |  |
| I 0893 | Multidrug Efflux Pump, Acriflavin Resistance Protein B | - | - | - | - | 1.6 | - |  |
| I 0926 | Multi-drug Resistance Protein A | -2.9 | -1.8^⁪^ | - | - | - | - | [5] |
| I 0945 | 6-Aminohexanoate-Dimer Hydrolase | -2.4 | -2.2 | - | - | - | - |  |
| I 1883 | Pantothenate Kinase | -1.9^⁪^ | -1.8 | -2.3^⁪^ | - | - | - |  |
| II 0258 | Bacitracin Resistance Protein, BacA | 1.5 | -2.3 | -2.4^⁪^ | - | - | - |  |
| II 0319 | 6-Aminohexanoate-Dimer Hydrolase | - | 2.0 | 2.1^⁪^ | - | - | -2.9^⁪^ |  |
| II 0452 | Type I Restriction-Modification Enzyme | -1.7^⁪^ | -1.8 | - | - | - | - |  |
| II 0795 | MDR Protein B | - | - | -2.0 | - | 1.8 | - |  |
| **Energy Production and Conversion** | | | | | | | | |
| I 0017 | Alkanal Monooxygenase α-Chain | - | -1.6 | -1.8^⁪^ | 2.0 | 2.1 | - |  |
| I 0911 | NifU Protein | 2.0 | -1.6^⁪^ | -1.6^⁪^ | - | - | - |  |
| I 0928 | Acetate CoA-Transferase α-Subunit | - | - | - | - | - | - |  |
| I 0967 | NAD-Dependant Malic Enzyme, Phosphate Acetyltransferase | - | - | -1.5^⁪^ | -1.5 | - | - |  |
| I 1016 | Fumarate Hydratase Class I | - | -1.9 | - | - | - | - |  |
| I 1145 | NADH Dehydrogenase Subunit N | - | -2.0 | -1.8^⁪^ | - | - | - |  |
| I 1149 | NADH Dehydrogenase Subunit J | - | -2.2 | -2.1 | - | - | -1.5^⁪^ |  |
| I 1527 | Glycolate Oxidase Subunit GlcD | -1.5 | - | - | - | - | 1.7^⁪^ |  |
| I 1559 | Salicylaldehyde Dehydrogenase | 1.7^⁪^ | 2.3 | - | -1.6^⁪^ | - | - |  |
| I 1591 | Ferredoxin-NADP Reductase | - | -1.7 | -2.6^⁪^ | - | - | - |  |
| I 1802 | NADP-Dependent Malic Enzyme | - | 2.4 | 2.3^⁪^ | -1.5^⁪^ | - | - |  |
| I 1900 | Cytochrome O Ubiquinol Oxidase Subunit I | - | -1.6 | - | -1.5^⁪^ | -1.9 | - |  |
| I 1903 | Cytochrome C-552 | - | - | - | 1.8 | 1.5^⁪^ | - |  |
| I 2037 | Phosphoenolpyruvate Carboxykinase | -1.8 | -1.6 | - | - | 1.6 | - |  |
| II 0061 | 2-Oxoisovalerate Dehydrogenase β Subunit | - | -2.1 | - | - | - | - |  |
| II 0074 | Thiosulfate Reductase Cytochrome B Subunit | 2.8 | - | -2.9 | -1.8 | - | - |  |
| II 0135 | 5-Carboxymethyl-2-Hydroxymuconate | - | 2.3 | - | - | - | - |  |
| II 0218 | Dihydrolipoamide Acetyltransferase | - | -2.0 | -1.5^⁪^ | -1.5^⁪^ | - | - |  |
| II 0224 | Formyl-Coenzyme A Transferase | - | 1.8^⁪^ | - | -1.9 | - | - |  |
| II 0225 | 6-Oxohexanoate Dehydrogenase | -3.2 | -2.1^⁪^ | - | - | -1.6^⁪^ | - |  |
| II 0242 | Aldehyde Dehydrogenase | -1.8^⁪^ | -2.3 | - | - | - | - |  |
| II 0394 | Glycerol Trinitrate Reductase | -1.9 | -2.4 | - | 2.1 | - | - |  |
| II 0429 | Erythritol-4-Phosphate Dehydrogenase | - | - | - | 1.5 | - | 1.5^⁪^ | [1, 5] |
| II 0553 | Alcohol Dehydrogenase | 1.8 | - | -1.9^⁪^ | - | - | 4.9 |  |
| II 0786 | NADH Dehydrogenase | - | -1.5^⁪^ | -2.1^⁪^ | -1.7 | -1.7 | - |  |
| II 0880 | Acetate Kinase | 1.5 | - | - | - | - | - |  |
| II 0952 | Nitrate Reductase ∆ Chain | - | - | -1.5^⁪^ | 1.7 | - | - |  |
| II 0965 | Pseudoazurin | -1.7^⁪^ | - | 1.5^⁪^ | - | -1.8 | - |  |
| II 0974 | Nitrous Oxide Reductase | -1.6^⁪^ | -3.2 | - | - | -1.7 | -2.1^⁪^ |  |
| II 1064 | (S)-2-Hydroxy-Acid Oxidase Chain D | -2.8 | -2.0^⁪^ | - | - | - | - |  |
| II 1073 | Cytochrome B561 | - | -2.0 | - | -1.8 | - | - |  |
| **General or Unknown Function** | | | | | | | | |
| I 0125 | Acetyltransferase | - | -1.8 | - | - | - | 3.7^⁪^ |  |
| I 0129 | Hydroxyacylglutathione Hydrolase | - | - | - | 1.5 | - | - |  |
| I 0206 | GTP Binding Protein | - | 2.3 | - | - | - | - |  |
| I 0273 | GlcG Protein | -2.7 | -2.3 | - | - | - | - |  |
| I 0394 | 2-Deoxy-D-Gluconate 3-Dehydrogenase | - | 1.9 | 1.6^⁪^ | -1.6^⁪^ | -1.7 | - |  |
| I 0709 | 4-Hydroxyphenylacetate 3-Monooxygenase | -1.7 | - | - | -1.7^⁪^ | - | 2.4^⁪^ |  |
| I 0739 | Integral Membrane Protein (Rhomboid Family) | - | -1.6 | - | -1.9 | -1.8 | 2.3^⁪^ |  |
| I 0830 | Outer Membrane Protein, Omp89 | - | - | - | -1.6 | - | - |  |
| I 0852 | Methyltransferase | - | -2.1 | - | - | - | - |  |
| I 0920 | MazG Protein | - | -1.9 | - | - | - | - |  |
| I 0922 | Sodium, Bile Acid Cotransporter Homolog, SBF Family | - | -2.2 | -3.1^⁪^ | - | - | -2.3^⁪^ |  |
| I 0925 | Alcohol Dehydrogenase | 1.8^⁪^ | 2.8 | - | - | - | - |  |
| I 0982 | DME Family Transporter | - | - | - | -1.8 | - | - |  |
| I 0995 | Secretion Activator Protein | - | - | - | 1.8 | - | -1.6^⁪^ |  |
| I 1034 | HesB Protein | 1.5^⁪^ | 1.6 | - | - | - | - |  |
| I 1038 | Phenylacetic Acid Degradation Protein, PaaD | -1.6 | - | - | 1.8 | - | - |  |
| I 1102 | Predicted Aspartyl Protease | - | -1.8 | -2.1^⁪^ | - | - | 2.0^⁪^ |  |
| I 1119 | Predicted Esterase of the α/β Hydrolase Fold | -1.7 | - | - | - | - | - |  |
| I 1143 | Metal Dependent Hydrolase | -1.8 | -2.1 | - | - | - | - | [5] |
| I 1239 | Predicted Permease | -1.5^⁪^ | -1.7^⁪^ | - | 2.0 | - | - |  |
| I 1446 | Phosphoglycolate Phosphatase | - | -1.9 | -1.6^⁪^ | - | - | - |  |
| I 1470 | YicC Protein | -1.8 | - | - | - | - | 1.6^⁪^ |  |
| I 1499 | Pirin | - | - | -2.2^⁪^ | -1.7^⁪^ | -2.0^⁪^ | - | [5] |
| I 1501 | Transglycosylase Associated Protein | - | -1.8 | - | - | - | - |  |
| I 1502 | Alkaline Phosphatase Like Protein | - | -1.8 | - | -1.5^⁪^ | - | - |  |
| I 1597 | Murein Hydrolase Exporter | - | -2.6 | -2.7^⁪^ | - | - | - |  |
| I 1764 | Oxidoreductase | -1.6 | - | - | - | -1.5^⁪^ | - |  |
| I 1820 | Acetyltransferase | -2.3^⁪^ | -2.9 | - | - | - | - |  |
| I 1822 | S-Formylglutathione Hydrolase | -1.5 | - | - | - | - | - |  |
| I 2003 | Trans-1,2-Dihydrobenzene-1,2-Diol Dehydrogenase, D-Xylose 1-Dehydrogenase | -2.2 | -2.3 | - | -2.3 | -2.0 | - |  |
| II 0211 | Penicillin Acylase | -1.9 | -2.7 | -1.9^⁪^ | -1.7 | - | -2.5^⁪^ |  |
| II 0234 | Ubiquinone Biosynthesis Protein, AarF | - | 1.9 | - | - | - | - |  |
| II 0307 | Vegetatible Incompatibility Protein HET-E-1 | - | -1.8 | -2.1^⁪^ | - | - | 1.6^⁪^ | Ficht, u.p. |
| II 0347 | Membrane Protein Related to Metalloendopeptidase | - | 1.8 | - | - | - | 1.7^⁪^ |  |
| II 0400 | Putative DNA-Binding Protein | -1.6^⁪^ | -1.7 | - | - | - | -2.1^⁪^ |  |
| II 0410 | Acetylglutamate Kinase | - | -1.9 | - | - | - | - |  |
| II 0448 | Zinc Metallopeptidase | - | -1.8 | -1.7^⁪^ | - | - | - |  |
| II 0574 | Myo-Inositol 2-Dehydrogenase | -1.8 | - | - | - | -1.5^⁪^ | - |  |
| II 0578 | Alkyl Hydroperoxide Reductase Subunit D | -1.6 | - | - | - | - | - |  |
| II 0611 | Integral Membrane Protein, Predicted Permease | -1.9 | -1.5^⁪^ | - | 1.8 | - | 1.5^⁪^ |  |
| II 0655 | Alkaline Phosphatase | - | 2.1 | - | -3.2 | - | - |  |
| II 0677 | DME Family Transporter | - | - | - | 1.6 | - | - |  |
| II 0865 | 1-Carboxy-3-Chloro-3,4-Dihydroxycyclo Hexa-1,5-Diene Dehydrogenase | -1.9^⁪^ | -1.9 | - | - | -2.4 | - |  |
| II 0866 | Oxidoreductase | -1.7 | - | - | -2.2 | - | - |  |
| II 0980 | Ribitol 2-dehydrogenase | - | -2.1 | - | - | - | 1.6^⁪^ |  |
| II 1036 | Zinc Protease | - | -2.0 | - | - | - | - |  |
| II 1037 | Zinc Protease | - | - | - | 1.9 | - | - | [3] |
| II 1060 | 2,5-Diketo-D-Gluconic Acid Reductase | 1.6 | 1.5 | - | - | - | - |  |
| II 1100 | Cellobiose Phosphotransferase System Protein, CelC | - | - | - | -1.9 | - | - |  |
| **Inorganic Ion Transport and Metabolism** | | | | | | | | |
| I 0044 | Putative Mg^2+^ and Co^2+^ Transporter, CorB | - | - | - | 1.7 | - | -1.5^⁪^ |  |
| I 0053 | Cation Transporting ATPase, PacS | - | 2.2 | 2.3^⁪^ | - | - | - |  |
| I 0284 | ABC-Type Thiamine Transport System, ThiP | - | -2.0 | - | - | - | 1.5^⁪^ |  |
| I 0317 | Integral Membrane Protein, TerC | -1.9 | -1.7 | - | - | -1.7^⁪^ | - |  |
| I 0622 | K^+^ System Potassium Uptake Protein | - | - | - | -1.7 | - | 3.7^⁪^ |  |
| I 0698 | Sulfite Exporter TauE/SafE | - | - | - | - | 1.6 | 2.6^⁪^ |  |
| I 0992 | Arsenate Reductase | -2.0 | -1.9 | - | - | -1.5^⁪^ | - |  |
| I 1292 | MFS Family, Nitrate/Nitrite Transporter | - | - | -2.7^⁪^ | -2.3 | - | 3.4^⁪^ |  |
| I 1367 | Superoxide Dismutase | -1.9 | - | - | - | - | - |  |
| I 1753 | CysQ Protein | - | 2.1 | 1.6^⁪^ | - | - | - |  |
| I 1754 | Binfunctional Sulfate Adenylyltransferase Subunit 1, Adenylylsulfate Kinase Protein | 1.6^⁪^ | 2.1 | 2.6^⁪^ | -1.5^⁪^ | - | 1.5^⁪^ |  |
| II 0056 | Mg^2+^ Transport, P-Type | - | -1.8 | - | - | - | - | [7] |
| II 0097 | Cation Transport, P-Type | - | -1.7 | - | - | -1.7 | - |  |
| II 0109 | Aliphatic Sulfonates-Binding Lipoprotein | - | -1.8 | - | - | -1.6^⁪^ | - |  |
| II 0606 | ABC-Type Enterochelin Transport System | - | -2.4 | - | - | - | 1.7^⁪^ |  |
| II 0704 | Bacterioferritin | -1.8^⁪^ | - | - | -1.6 | - | - |  |
| II 0897 | Chloride Channel Protein, EriC | - | 2.2 | 1.6^⁪^ | - | - | - |  |
| II 0964 | Asparagine Synthetase B (Glutamine-Hydrolyzing) | -2.1^⁪^ | -2.0 | -1.7^⁪^ | - | - | - |  |
| **Intracellular Trafficking, Secretion and Vesicular Transport** | | | | | | | | |
| I 0365 | Biopolymer Transport Protein, ExbB | -1.7 | - | - | - | -1.5^⁪^ | - |  |
| I 0883 | Multiple Antibiotic Resistance Protein, MarC | - | - | - | 1.8 | 1.6^⁪^ | - |  |
| II 1013 | Hypothetical Protein, VceA | - | - | - | 1.7 | - | - |  |
| **Lipid Transport and Metabolism** | | | | | | | | |
| I 0022 | 3-Hydroxybuturyl CoA Dehydratase | 1.7^⁪^ | 1.9 | - | - | - | - |  |
| I 0552 | Lysophospholipase L2 | -2.7^⁪^ | -2.3 | - | - | - | -1.5^⁪^ |  |
| I 0799 | Methylmalonyl-CoA Mutase | - | 1.6 | - | - | - | - |  |
| I 0827 | Undecaprenyl Pyrophosphate Synthetase | -1.6^⁪^ | 2.7^⁪^ | - | -1.5 | - | 1.6^⁪^ | [1] |
| I 1196 | Enoyl-CoA Hydratase | - | -2.0 | -2.8^⁪^ | - | - | - | Ficht, u.p. |
| I 1252 | CDP-Diacylglycerol--G3P3-Phosphatidyltransferase | - | -2.2 | -2.5^⁪^ | - | - | -1.6^⁪^ |  |
| I 1289 | 4’-Phosphopantetheinyl Transferase | -1.5 | -1.8 | - | - | - | - |  |
| I 1922 | Acetoacetyl-CoA Synthase | -1.5 | -1.8 | - | - | - | - |  |
| I 1928 | Enoyl-CoA Hydratase | - | -1.8 | - | - | - | - |  |
| I 1956 | 3-Hydroxydecanoyl-ACP Dehydratase | - | - | - | 1.6 | - | - |  |
| I 1977 | 1-Acyl-SN-G3P Acyltransferase | -1.5^⁪^ | -2.5 | - | - | - | - |  |
| II 0643 | 3-Oxoadipate CoA-Transferase Subunit A | - | -2.1 | -3.1^⁪^ | - | - | -1.6^⁪^ |  |
| II 0646 | Acetyl-CoA Acetyltransferase | -1.7 | -1.5^⁪^ | - | 1.6 | - | -1.6^⁪^ |  |
| II 0815 | Acetyl-CoA Synthetase | - | -1.8 | -1.8^⁪^ | - | - | - |  |
| II 1103 | Phosphatidylglycero-Phosphatase B | - | -1.7 | -1.5^⁪^ | -2.3 | -2.2 | 2.0^⁪^ |  |
| **Nucleotide Transport and Metabolism** | | | | | | | | |
| I 0082 | Hypoxanthine-Guanine Phosphoribosyltransferase H | -1.5 | -1.5^⁪^ | -1.6^⁪^ | 1.8 | - | -2.2^⁪^ | [2] |
| I 0476 | Adenine Phosphoribosyltransferase | - | - | - | 1.6 | - | - |  |
| I 0608 | Thymidylate Synthase | - | - | - | 1.6 | - | - |  |
| I 0989 | Thymidylate Kinase | 1.5 | 1.8^⁪^ | - | - | - | - |  |
| I 1090 | Deoxyguanosinetriphosphate Triphosphohydrolase-Like Protein | - | -1.6 | -1.6^⁪^ | 2.1 | 1.7 | - |  |
| I 1117 | Adenylosuccinate Lyase | - | 1.7^⁪^ | - | -1.8 | -1.6^⁪^ | 1.5^⁪^ |  |
| I 1318 | AMP Nucleosidase | - | 1.7 | - | 1.7 | 1.7^⁪^ | - |  |
| I 1430 | Ureidoglycolate Hydrolase | -1.7 | -1.7 | - | -1.8 | -2.5 | - |  |
| I 1611 | Dihydroorotate Dehydrogenase | - | 1.8^⁪^ | - | -1.6 | - | 3.1^⁪^ | [1] |
| I 1772 | Putative Deoxyribonucleotide Triphosphate Pyrophosphatase | -1.5 | - | -1.7^⁪^ | - | - | - |  |
| II 0088 | Nucleoside Hydrolase | -2.9 | -2.5 | - | - | - | - |  |
| II 0369 | Atrazine Chlorohydrolase | - | 1.9 | - | - | - | - |  |
| II 0420 | Thymidylate Synthase | -1.8 | - | - | -1.5^⁪^ | - | - |  |
| II 0627 | Probable Adenine Deaminase | -1.9 | - | - | 1.6 | - | - |  |
| **Posttranslational Modification, Protein Turnover and Chaperones** | | | | | | | | |
| I 0004 | Thioredoxin Domain Protein | 1.7^⁪^ | 2.3 | 1.6^⁪^ | -1.8^⁪^ | -1.7^⁪^ | - |  |
| I 0093 | ExsD Protein | - | -1.9 | -2.7^⁪^ | - | - | - |  |
| I 0783 | Protease DO | - | 2.1 | 2.0^⁪^ | - | -1.5^⁪^ | - |  |
| I 0958 | Thioredoxin Reductase | -1.5^⁪^ | -2.2 | -2.0^⁪^ | - | - | - |  |
| I 1049 | Bacterioferritin Comigratory Protein | - | - | -1.7^⁪^ | 1.6 | - | - | [8] |
| I 1080 | Protein-L-Isoaspartate O-Methyltransferase | - | - | -1.6^⁪^ | 1.5 | - | - |  |
| I 1172 | Cytochrome C Oxidase Assembly Protein Cox15 | 1.5^⁪^ | 2.0 | - | - | - | - |  |
| I 1463 | Cytochrome C Oxidase Assembly Protein | -1.8 | -2.0^⁪^ | - | 1.8 | - | - |  |
| I 1464 | Protoheme IX Farnesyltransferase | -1.6^⁪^ | -2.4 | - | - | -2.1 | - |  |
| I 1650 | Urease Accessory Protein, UreF | -2.3 | -1.5^⁪^ | - | - | - | - |  |
| I 1784 | Small Heat Shock Protein, HspA | -1.7 | - | - | - | - | - |  |
| I 1804 | PII Uridylyl-Transferase | 3.2 | - | - | 2.3 | -2.4 | - | [1, 2, 5] |
| I 1808 | NifU-Like Protein | -2.2 | - | 2.0^⁪^ | - | - | - |  |
| I 1994 | Mg^2+^ Chelatase Family Protein | 2.3^⁪^ | 3.6 | - | - | - | - |  |
| I 2048 | ATP-Dependent Protease ATP Binding Subunit | - | - | - | 1.6 | - | - |  |
| II 0042 | Heat Shock Protein A | -1.6 | - | - | - | - | - |  |
| **Replication, Recombination and Repair** | | | | | | | | |
| I 0040 | Tyrosine Recombinase | -1.7 | -1.8 | - | 1.6 | - | - | [5, 9] |
| I 0333 | Holiday Junction DNA Helicase, RuvA | - | -1.7 | -1.7^⁪^ | - | - | 1.8^⁪^ |  |
| I 0334 | Holliday Junction DNA Helicase, RuvB | - | -1.7 | - | - | - | 2.3^⁪^ | [5] |
| I 0728 | Single-Stranded-DNA-DNA-Specific Exonuclease, RecJ | - | - | -2.1^⁪^ | -1.7 | - | - |  |
| I 0784 | ATPase Protein | - | 2.2 | - | -1.7 | -1.5^⁪^ | - |  |
| I 0902 | Recombinase | -2.1 | -2.4 | - | - | - | -1.7^⁪^ |  |
| I 1002 | Transposase | -1.6 | -1.7 | - | - | - | - |  |
| I 1053 | Transposase | -2.3 | -4.2 | - | - | - | 1.5^⁪^ |  |
| I 1093 | Exodeoxyribonuuclease III | - | -2.1 | - | - | - | 2.3^⁪^ |  |
| I 1097 | Uracil-DNA Glycosylase | -2.1^⁪^ | -2.3 | -2.9^⁪^ | - | -1.8 | - |  |
| I 1163 | Transposase | - | -2.1 | - | - | - | - |  |
| I 1223 | Transposase | -2.1 | -1.7^⁪^ | - | - | - | - |  |
| I 1362 | ATPase | -2.4 | - | - | -1.6^⁪^ | -1.7^⁪^ | - |  |
| I 1397 | Transposase | - | -2.0 | - | - | - | - |  |
| I 1420 | Transposase | -1.8 | - | - | - | - | - |  |
| I 1424 | Transposase | - | - | - | -2.1 | - | 2.8^⁪^ |  |
| I 1442 | A/G-Specific Adenine Glycosylase | - | -1.8 | - | - | - | - |  |
| I 1664 | Recombinase XerD | -2.2 | -1.9 | - | 2.5 | 1.9 | 4.2^⁪^ |  |
| I 1818 | ATP Dependent Helicase, HrpB | - | -2.0 | - | - | -1.9 | 2.0^⁪^ |  |
| I 1876 | DNA Polymerase III, α Subunit | - | -1.8 | - | -1.5^⁪^ | - | 3.5^⁪^ |  |
| I 1908 | DNA Polymerase III, δ and τ Subunits | -1.5 | - | - | - | - | 1.7^⁪^ |  |
| I 1941 | RecF Protein | 1.5^⁪^ | 2.5 | - | - | - | - |  |
| I 1942 | DNA Polymerase III β Subunit | - | - | - | -1.6 | - | 2.0^⁪^ | Ficht, u.p. |
| I 1946 | Formamidopyrimidine-DNA Glycosylase | 1.6^⁪^ | 2.3 | - | -1.5^⁪^ | - | - | [9] |
| I 2015 | Dinucleoside Polyphosphate Hydrolase | - | - | - | 1.5 | - | 1.5^⁪^ |  |
| II 0183 | Transposase | -3.5 | -3.2 | 2.9^⁪^ | - | -1.7 | -3.3^⁪^ |  |
| II 0184 | Transposase | -1.9 | -1.7^⁪^ | - | - | - | - |  |
| II 0227 | Transposase | -2.0 | -1.9 | - | - | - | - |  |
| II 0445 | Transposase | -2.6 | -2.5 | - | 1.5 | - | - |  |
| II 0453 | Transposase | -1.5^⁪^ | -1.9 | -1.6^⁪^ | - | - | - |  |
| II 0714 | Transposase | -2.2^⁪^ | -1.9 | - | - | - | - |  |
| II 0718 | Transposase | -1.5^⁪^ | -2.0 | - | - | - | - |  |
| II 1038 | Methyltransferase | -2.0 | - | - | 1.7 | - | -2.6^⁪^ |  |
| **Secondary Metabolites Biosynthesis, Transport and Catabolism** | | | | | | | | |
| I 0032 | 3-OxoAcyl-(ACP) Reductase | -1.9^⁪^ | -1.5^⁪^ | - | 1.8 | - | 2.3^⁪^ |  |
| I 1167 | Putative Aromatic Compound Catabolism Protein | 1.8^⁪^ | 2.6 | 1.6^⁪^ | - | - | - |  |
| I 1504 | Acetylspermidine Deacetylase | -2.6 | -1.7^⁪^ | - | -2.7^⁪^ | - | - | Ficht, u.p. |
| II 0062 | Probable Carbonyl Reductase [NADPH] | - | -1.5 | - | -1.9 | -1.6^⁪^ | 1.7^⁪^ |  |
| II 0079 | Isochorismatase | - | 2.0 | 2.2^⁪^ | - | - | 1.8^⁪^ |  |
| II 0580 | Probable Blue-Copper Protein, YacK | -2.7 | -1.8 | - | -1.9 | -2.0^⁪^ | -1.9^⁪^ |  |
| II 0879 | Putative Cytochrome P450, YjiB | -1.8 | -2.4 | - | - | - | - |  |
| II 0889 | Phenylacetic Acid Degradation Protein, PaaI | - | -2.0 | 2.4^⁪^ | - | - | -1.5^⁪^ |  |
| **Signal Transduction Mechanisms** | | | | | | | | |
| I 0929 | Diguanylate Cyclase/Phosphodiesterase Domain 1, GGDEF Domain | 1.9 | 2.6^⁪^ | - | - | - | - |  |
| I 0950 | Phosphohistidine Phosphatase Protein, SixA | -2.7 | -3.0 | - | - | - | - |  |
| I 1678 | Hypothetical Protein, Sensory Transduction Protein Kinase | - | - | -2.2^⁪^ | -2.1 | - | - |  |
| I 1811 | Acid Tolerance Regulatory Protein, ActR | - | - | - | 1.7 | - | - |  |
| II 0654 | Diguanylate Cyclase/Phosphodiesterase Domain 1, GGDEF Domain | 1.5^⁪^ | -2.5 | -3.2^⁪^ | - | - | -1.8^⁪^ |  |
| II 0659 | Two Component Response Regulator | - | 1.9^⁪^ | - | -1.8 | - | - | [3] |
| **Transcription** | | | | | | | | |
| I 0387 | IclR Family | - | - | -5.9^⁪^ | - | -1.9 | -4.2^⁪^ |  |
| I 0604 | TetR Family | - | - | -1.8^⁪^ | 1.5 | - | - |  |
| I 0623 | TetR Family | - | -2.6 | -1.5^⁪^ | - | -2.0 | - |  |
| I 0891 | TetR Family | - | - | -2.2^⁪^ | 1.6 | - | - |  |
| I 0896 | LysR Family | - | -2.1 | -1.9^⁪^ | - | - | 1.6^⁪^ |  |
| I 1598 | LysR Family | - | -1.9 | -2.8^⁪^ | - | - | - |  |
| I 1776 | Heat-Inducible Transcription Repressor, HrcA | - | -1.7 | - | - | - | - | [5] |
| II 0345 | LysR Family | -1.9^⁪^ | - | - | - | 1.8 | - |  |
| II 0370 | GntR Family, HutC | -1.8 | - | - | - | - | - |  |
| II 0520 | MarR Family | -1.9 | - | - | - | - | - |  |
| II 0576 | LysR Family | -1.8 | - | - | - | - | - |  |
| II 0641 | AraC-Type DNA-Binding Domain | - | - | - | -1.6 | - | - |  |
| II 0807 | GntR Family | -1.8 | - | - | - | - | - |  |
| II 0894 | LysR Family Hydrogen Peroxide-Inducible Gene Activator | - | - | -2.2^⁪^ | -1.7 | - | - |  |
| II 0966 | CRP Family | - | - | - | 1.6 | - | - |  |
| II 1007 | GntR Family | - | - | - | 1.6 | - | - |  |
| II 1116 | LuxR Family | -2.3 | - | - | -1.9 | - | - | [3, 5] |
| **Translation, Ribosomal Structure and Biogenesis** | | | | | | | | |
| I 0277 | Heat Shock Protein 15 | - | 1.6 | - | - | - | - |  |
| I 0322 | 50S Ribosomal Protein L31 | - | 1.5^⁪^ | - | 1.8 | - | - |  |
| I 0327 | Protein Translation Elongation Factor P (EF-P) | -2.0^⁪^ | -2.6 | - | - | - | 1.6^⁪^ |  |
| I 0428 | tRNA (5-Methylaminomethyl-2-Thiouridylate)-Methyltransferase | -1.7^⁪^ | -2.2 | - | - | - | - |  |
| I 0429 | 23S Ribosomal RNA Methyltransferase | - | - | -1.7^⁪^ | -1.8 | - | 1.6^⁪^ |  |
| I 0444 | Methyltransferase | 1.5^⁪^ | 2.7 | 2.4^⁪^ | - | - | - |  |
| I 0480 | Peptidyl-tRNA Hydrolase | 2.1 | 2.4 | - | 1.5^⁪^ | 2.7 | - | [10] |
| I 0747 | 50S Ribosomal Protein L10P | -1.6 | - | - | - | -1.6^⁪^ | - |  |
| I 0752 | 30S Ribosomal Protein S12 | - | - | -2.0^⁪^ | 1.5 | - | - |  |
| I 0779 | 30S Ribosomal Protein S13 | 1.6^⁪^ | 2.1 | 1.5^⁪^ | - | - | - |  |
| I 0890 | Queuine tRNA-Ribosyltransferase | -1.7^⁪^ | -1.9 | - | - | - | - |  |
| I 0987 | Methionine—tRNA Ligase | - | - | - | 1.6 | - | - |  |
| I 1089 | Arginyl-tRNA Synthetase | -1.5 | -1.5^⁪^ | - | - | -1.5^⁪^ | - |  |
| I 1103 | NifR3-Like Protein | - | -1.8 | - | - | - | - |  |
| I 1184 | Small Protein A | - | 1.5^⁪^ | - | 1.5 | - | - |  |
| I 1203 | Ribonuclease D | - | 1.5^⁪^ | - | -2.2 | - | 3.8^⁪^ |  |
| I 1267 | Dimethyladensine Transferase, KsgA | - | - | - | -1.5 | - | 1.6^⁪^ |  |
| I 1360 | Glutamyl-tRNA(GLN) Amidotransferase Subunit A, Amidase | - | 2.6^⁪^ | - | -2.3 | -1.8^⁪^ | 1.6^⁪^ |  |
| I 1862 | 2-5 RNA Ligase | - | -1.8 | - | - | - | - | Ficht, u.p. |
| I 1961 | Polyribonucleotide Nucleotidyltransferase | - | 1.5 | - | - | - | - |  |
| II 0002 | Ribosomal Protein Serine Acetyltransferase | - | - | -2.1^⁪^ | -1.9 | - | 1.5^⁪^ |  |
| II 0289 | Glutamyl-tRNA(GLN) Amidotransferase Subunit A | - | 2.0 | - | - | - | - |  |
| II 0500 | Lysyl-tRNA Synthetase | - | -2.2 | -1.5^⁪^ | - | - | - |  |
| II 0675 | Glutamyl-tRNA Amidotransferase Subunit A | - | 1.8^⁪^ | - | 1.5 | - | - |  |
| II 0812 | Peptide Deformylase | -1.6^⁪^ | - | - | 1.8 | 1.5^⁪^ | - | Ficht, u.p. |
| II 1039 | tRNA Pseudouridine Synthase A | 1.6 | 1.6^⁪^ | - | 1.8 | -1.6^⁪^ | - |  |
| II 1056 | Histidyl-tRNA Synthetase | - | - | - | -1.8 | -1.7 | 1.8^⁪^ |  |
| II 1072 | Novel RNA Polymerase II Holoenzyme | -2.2 | -2.6^⁪^ | - | -1.7 | - | - |  |
| **Unknown and Other** | | | | | | | | |
| I 0011 | Hypothetical Protein | - | 2.2 | - | - | - | - |  |
| I 0038 | Hypothetical Protein | -2.0^⁪^ | -2.2 | - | 1.6^⁪^ | - | - |  |
| I 0041 | Hypothetical Protein | - | -1.7 | -2.2^⁪^ | - | - | - |  |
| I 0051 | Hypothetical Protein | - | 1.7^⁪^ | - | -1.9 | - | - |  |
| I 0052 | Hypothetical Protein | 1.9 | 1.9^⁪^ | - | -1.8 | - | - |  |
| I 0055 | Hypothetical Protein | 1.8^⁪^ | 2.6 | 3.8^⁪^ | - | -1.5^⁪^ | - |  |
| I 0057 | Hypothetical Membrane Spanning Protein | 1.5^⁪^ | 2.3 | 2.4^⁪^ | - | - | - |  |
| I 0065 | Hypothetical Protein | -1.6 | - | - | -1.7 | -1.5^⁪^ | 1.8^⁪^ |  |
| I 0154 | Hypothetical Membrane Spanning Protein | - | - | - | 1.9 | 2.0 | - |  |
| I 0172 | Hypothetical Protein | -1.8 | -1.5^⁪^ | - | - | - | - |  |
| I 0194 | Hypothetical Cytosolic Protein | - | -1.8 | -2.5^⁪^ | - | - | - |  |
| I 0212 | Hypothetical Protein | - | - | - | - | -1.9 | - |  |
| I 0220 | Hypothetical Protein | -2.3 | -1.7^⁪^ | - | - | -1.7^⁪^ | - |  |
| I 0262 | Hypothetical Protein | - | -1.8 | - | - | -2.5 | - |  |
| I 0290 | Hypothetical Cytosolic Protein | - | -1.8 | - | - | - | - |  |
| I 0304 | Hypothetical Cytosolic Protein | -1.5^⁪^ | - | - | -1.7^⁪^ | -1.9 | - | [5] |
| I 0308 | Hypothetical Protein | -1.9 | - | - | - | - | - |  |
| I 0354 | Hypothetical Membrane Spanning Protein | - | 1.7^⁪^ | - | 1.5 | - | - |  |
| I 0362 | Predicted Periplasmic protein | - | - | - | 2.1 | 1.7^⁪^ | - |  |
| I 0366 | Hypothetical Protein | - | 1.5^⁪^ | - | 1.8 | 1.7 | 1.7^⁪^ |  |
| I 0368 | Hypothetical Protein | 1.6 | 1.8^⁪^ | -1.7^⁪^ | 1.8 | 1.8 | - |  |
| I 0373 | Hypothetical Protein | 2.3 | 1.7^⁪^ | - | 1.9 | 1.6^⁪^ | - |  |
| I 0389 | Hypothetical Protein | -1.5^⁪^ | -1.9 | - | - | - | - |  |
| I 0400 | Hypothetical Protein | 1.7 | 2.3 | 1.8^⁪^ | -1.8 | -1.5^⁪^ | - |  |
| I 0419 | Hypothetical Protein | - | -1.6 | -2.4^⁪^ | - | - | - |  |
| I 0431 | Hypothetical Protein | - | -1.6 | -2.9^⁪^ | -1.6 | 1.6 | - |  |
| I 0443 | Hypothetical Protein | 2.0^⁪^ | 2.4 | - | - | - | - |  |
| I 0448 | Hypothetical Protein | 1.6^⁪^ | 1.9 | - | - | - | - |  |
| I 0515 | Hypothetical Protein | - | - | -2.2^⁪^ | 1.8 | 2.0 | - |  |
| I 0534 | Hypothetical Protein | - | -1.6 | -2.1^⁪^ | - | -2.2 | -9.8^⁪^ |  |
| I 0535 | Hypothetical Protein | 1.9 | - | -4.3^⁪^ | -1.5^⁪^ | - | - |  |
| I 0602 | Hypothetical Protein | - | - | -1.8^⁪^ | 2.1 | - | - |  |
| I 0620 | Hypothetical Protein | - | - | - | 1.8 | 1.9 | - |  |
| I 0627 | Hypothetical Protein | - | -2.1 | -2.0^⁪^ | -1.5^⁪^ | -1.5^⁪^ | - |  |
| I 0651 | Hypothetical Cytosolic Protein | -1.8^⁪^ | -1.9 | - | - | -1.9 | -2.0^⁪^ |  |
| I 0652 | Hypothetical Protein | 1.9 | - | -3.0^⁪^ | - | - | - |  |
| I 0691 | Hypothetical Protein | - | -1.7 | - | - | -2.0 | - |  |
| I 0699 | Hypothetical Protein | -1.7 | - | - | - | - | - |  |
| I 0723 | Hypothetical Protein | - | -2.6 | -2.7^⁪^ | - | - | - |  |
| I 0738 | Hypothetical Protein | - | - | - | 1.8 | - | - |  |
| I 0798 | Hypothetical Protein | - | 1.7^⁪^ | - | 2.0 | 2.0 | - |  |
| I 0806 | Hypothetical Protein | - | 1.5^⁪^ | -2.0^⁪^ | 1.8 | - | - |  |
| I 0822 | Hypothetical Protein | - | -1.9 | -1.7^⁪^ | - | -1.5^⁪^ | -1.6^⁪^ |  |
| I 0952 | Predicted Membrane Protein | - | - | - | 1.7 | - | - |  |
| I 0993 | Hypothetical Protein | - | - | - | 1.6 | - | -1.6^⁪^ |  |
| I 1000 | Hypothetical Protein | -3.3 | -2.8 | - | -1.5^⁪^ | - | - |  |
| I 1006 | Hypothetical Cytosolic Protein | - | -2.0 | - | - | -1.6^⁪^ | -2.8^⁪^ |  |
| I 1008 | Hypothetical Protein | -1.6^⁪^ | -2.2 | -1.6^⁪^ | - | - | - |  |
| I 1011 | Hypothetical Protein | -1.8^⁪^ | -2.8 | - | - | - | -3.1^⁪^ |  |
| I 1013 | Hypothetical Membrane Spanning Protein | - | -1.9 | -3.2^⁪^ | - | - | -2.2^⁪^ |  |
| I 1048 | Hypothetical Membrane Associated Protein | -2.2 | -1.6^⁪^ | - | - | - | - |  |
| I 1072 | Hypothetical Protein | -2.6 | - | - | - | - | - |  |
| I 1095 | Hypothetical Protein | -1.5^⁪^ | -2.1 | - | - | - | - |  |
| I 1107 | Hypothetical Cytosolic Protein | - | -2.0 | -1.9^⁪^ | - | - | - |  |
| I 1135 | Hypothetical Protein | -1.5 | - | - | - | - | - |  |
| I 1162 | Hypothetical Protein | -2.2^⁪^ | -2.8 | -2.6^⁪^ | -1.6^⁪^ | - | - |  |
| I 1165 | Hypothetical Membrane Spanning Protein | - | 2.2 | 1.9^⁪^ | - | - | - |  |
| I 1214 | Hypothetical Protein | 2.3 | 2.1^⁪^ | - | - | - | 1.8^⁪^ |  |
| I 1222 | Hypothetical Protein | 1.6^⁪^ | 2.1 | - | - | 2.1 | - |  |
| I 1242 | Hypothetical Membrane Spanning Protein | 1.7^⁪^ | - | - | 1.5^⁪^ | 1.7 | - |  |
| I 1290 | Hypothetical Membrane Spanning Protein | - | - | - | - | -1.8 | - |  |
| I 1314 | Hypothetical Protein | 1.6^⁪^ | 2.0 | - | - | - | - |  |
| I 1358 | Hypothetical Cytosolic Protein | - | 2.0^⁪^ | - | -1.5 | - | - |  |
| I 1361 | Hypothetical Cytosolic Protein | -1.8 | - | 1.6^⁪^ | -1.7^⁪^ | -1.7^⁪^ | - | [4] |
| I 1434 | Hypothetical Protein | -1.5^⁪^ | -2.2 | -1.5^⁪^ | - | - | - |  |
| I 1472 | Hypothetical Protein | - | -1.6 | - | -1.8^⁪^ | -1.9 | - |  |
| I 1524 | Hypothetical Protein | - | - | - | -1.5 | -1.8 | - |  |
| I 1572 | Predicted Membrane Protein | - | - | - | -1.5 | - | - |  |
| I 1595 | Hypothetical Protein | -1.5^⁪^ | -2.1 | -4.1^⁪^ | - | - | -2.3^⁪^ |  |
| I 1647 | Hypothetical Protein | - | - | - | -1.9 | - | 2.1^⁪^ | [5] |
| I 1658 | Hypothetical Protein | - | - | - | 1.5 | - | - | [1] |
| I 1660 | Hypothetical Protein | -1.8 | -1.7^⁪^ | - | 1.7 | - | - |  |
| I 1674 | Hypothetical Protein | - | -2.1 | -2.9^⁪^ | - | - | - |  |
| I 1680 | Hypothetical Protein | -2.2 | -2.2 | -1.9^⁪^ | - | - | - |  |
| I 1684 | Hypothetical Protein | - | - | -3.0^⁪^ | -1.8 | - | - |  |
| I 1685 | Hypothetical Protein | -1.7 | -2.3 | -2.9^⁪^ | - | - | -1.6^⁪^ |  |
| I 1690 | Hypothetical Protein | - | -1.7 | -5.9^⁪^ | -1.7^⁪^ | -2.0 | -3.3^⁪^ |  |
| I 1694 | Hypothetical Protein | - | -1.7 | -2.9^⁪^ | -2.1 | -2.5 | -2.2^⁪^ |  |
| I 1696 | Hypothetical Membrane Spanning Protein | - | - | -3.3^⁪^ | -1.7^⁪^ | -1.9 | -1.8^⁪^ |  |
| I 1699 | Hypothetical Protein | -1.9 | - | - | -1.5^⁪^ | - | - |  |
| I 1703 | Hypothetical Protein | -2.3 | - | - | - | - | - |  |
| I 1724 | Hypothetical Protein | - | -1.9 | - | - | - | - |  |
| I 1756 | Hypothetical Protein | -1.7 | - | - | - | - | - |  |
| I 1788 | Hypothetical Protein | - | -2.0 | - | - | - | - |  |
| I 1826 | Hypothetical Protein | -1.8 | -1.5^⁪^ | 2.6^⁪^ | -1.5^⁪^ | - | - |  |
| I 1842 | Hypothetical Protein | - | -2.0 | - | -1.8 | - | -2.5^⁪^ |  |
| I 1865 | Hypothetical Protein | -1.5^⁪^ | - | - | 1.6 | - | - |  |
| I 1891 | Hypothetical Protein | -1.7^⁪^ | -1.9 | - | - | - | -1.6^⁪^ |  |
| I 1920 | Hypothetical Protein | -1.7 | - | - | - | - | - |  |
| I 1929 | Hypothetical Protein | - | -2.5 | - | - | - | -3.0^⁪^ |  |
| I 1933 | Hypothetical Protein | - | - | -1.7^⁪^ | -1.8 | -1.5^⁪^ | -3.2^⁪^ |  |
| I 2006 | Hypothetical Cytosolic Protein | -1.9^⁪^ | -2.3 | - | - | - | - |  |
| I 2044 | Hypothetical Membrane Spanning Protein | - | 1.9^⁪^ | - | 1.7 | -2.0 | - |  |
| II 0022 | Hypothetical Protein | 1.8^⁪^ | - | -2.8^⁪^ | -1.5^⁪^ | -2.0 | -2.3^⁪^ |  |
| II 0057 | Hypothetical Protein | -2.0 | -1.9 | - | - | - | -1.5^⁪^ |  |
| II 0118 | Hypothetical Protein | - | -2.5 | - | - | - | -2.5^⁪^ |  |
| II 0153 | Hypothetical Protein | -1.6^⁪^ | -1.7 | - | -1.6 | - | - |  |
| II 0187 | Hypothetical Cytosolic Protein | -2.0 | - | - | -1.8^⁪^ | -1.9^⁪^ | - |  |
| II 0191 | Hypothetical Protein | -1.6^⁪^ | -1.9 | - | - | - | - |  |
| II 0231 | Hypothetical Protein | - | -1.6 | - | 1.9 | 1.6 | - |  |
| II 0244 | Hypothetical Protein | - | 2.0^⁪^ | - | - | -1.8 | - |  |
| II 0330 | Hypothetical Protein | -2.2^⁪^ | -2.4 | - | - | - | - |  |
| II 0331 | Hypothetical Cytosolic Protein | - | 1.9 | - | - | - | - |  |
| II 0379 | Hypothetical Protein | - | 1.8^⁪^ | - | 1.7 | - | - |  |
| II 0399 | Hypothetical Protein | 1.9^⁪^ | - | - | - | -1.5 | -2.0^⁪^ |  |
| II 0412 | Hypothetical Protein | - | -2.2 | - | - | - | -2.4^⁪^ |  |
| II 0480 | Hypothetical Protein | -1.5^⁪^ | -1.8 | - | -1.7^⁪^ | -1.6 | - |  |
| II 0503 | Hypothetical Protein | - | 1.6^⁪^ | - | -1.6 | -1.5^⁪^ | - |  |
| II 0516 | Hypothetical Protein, Predicted Membrane Protein | - | - | - | -2.8 | -2.4 | - |  |
| II 0525 | Hypothetical Protein | - | -1.9^⁪^ | - | 1.9 | - | -3.2^⁪^ |  |
| II 0534 | Hypothetical Protein | - | - | - | -1.5^⁪^ | -1.7 | - |  |
| II 0595 | Hypothetical Protein | - | - | -1.6^⁪^ | 1.7 | - | - |  |
| II 0615 | Hypothetical Protein | - | - | - | 1.6 | - | - |  |
| II 0647 | Hypothetical Protein | -1.6^⁪^ | -2.8 | 1.7^⁪^ | -1.5^⁪^ | - | -3.2^⁪^ |  |
| II 0693 | Hypothetical Cytosolic Protein | -1.5^⁪^ | -2.3 | - | - | - | -2.9^⁪^ |  |
| II 0726 | Hypothetical Protein | -2.3 | - | - | - | - | - |  |
| II 0732 | Hypothetical Protein | - | - | - | - | -1.6 | - |  |
| II 0788 | Hypothetical Protein | - | - | -2.9^⁪^ | 1.6 | - | - |  |
| II 0833 | Hypothetical Protein | - | -1.9 | -2.0^⁪^ | - | - | -3.4^⁪^ |  |
| II 0841 | Hypothetical Protein | - | -2.2 | - | - | - | -3.1^⁪^ |  |
| II 0842 | Hypothetical protein | - | - | - | -1.7 | - | - |  |
| II 0877 | Hypothetical Protein | -2.1^⁪^ | -2.6 | - | - | -1.5^⁪^ | 2.9^⁪^ |  |
| II 0913 | Predicted Membrane Protein | -1.7^⁪^ | -3.4 | - | - | -2.2 | 1.6^⁪^ |  |
| II 0918 | Hypothetical Protein | - | - | - | 1.6 | - | -1.6^⁪^ |  |
| II 0919 | Hypothetical Protein | - | - | - | 1.7 | - | - |  |
| II 1138 | Hypothetical Protein | - | -1.8 | -3.9^⁪^ | -1.5^⁪^ | -2.2 | -4.6^⁪^ |  |

A (–) indicates genes excluded for technical reasons or had a fold change of less than 1.5,

^⁪^genes that did not pass the statistical significance test but showed an average alteration of at least 1.5-fold. Fold change values are the averaged log_2_ ratio of normalized signal values from two independent statistical analyses. Abbreviations are as follows: STM, Signature Tagged Mutagenesis; AA, Amino Acid; OMP, Outer Membrane Protein; G3P, Glycerol 3 Phosphate; DME, Drug/Metabolite Exporter; ACP, Acyl Carrier Protein

**References**

1. Kohler S, Foulongne V, Ouahrani-Bettache S, Bourg G, Teyssier J, Ramuz M, Liautard JP: **The analysis of the intramacrophagic virulome of *Brucella* *suis* deciphers the environment encountered by the pathogen inside the macrophage host cell**. *Proc Natl Acad Sci U S A* 2002, **99**(24):15711-15716.

2. Foulongne V, Bourg G, Cazevieille C, Michaux-Charachon S, O'Callaghan D: **Identification of *Brucella* *suis* genes affecting intracellular survival in an in vitro human macrophage infection model by signature-tagged transposon mutagenesis**. *Infect Immun* 2000, **68**(3):1297-1303.

3. Delrue RM, Lestrate P, Tibor A, Letesson JJ, De Bolle X: ***Brucella* pathogenesis, genes identified from random large-scale screens**. *FEMS Microbiol Lett* 2004, **231**(1):1-12.

4. Lestrate P, Dricot A, Delrue RM, Lambert C, Martinelli V, De Bolle X, Letesson JJ, Tibor A: **Attenuated signature-tagged mutagenesis mutants of *Brucella* *melitensis* identified during the acute phase of infection in mice**. *Infect Immun* 2003, **71**(12):7053-7060.

5. Wu Q, Pei J, Turse C, Ficht TA: **Mariner mutagenesis of *Brucella melitensis* reveals genes with previously uncharacterized roles in virulence and survival**. *BMC Microbiol* 2006, **6**:102.

6. Allen CA, Adams LG, Ficht TA: **Transposon-derived *Brucella abortus* rough mutants are attenuated and exhibit reduced intracellular survival**. *Infect Immun* 1998, **66**(3):1008-1016.

7. Lestrate P, Delrue RM, Danese I, Didembourg C, Taminiau B, Mertens P, De Bolle X, Tibor A, Tang CM, Letesson JJ: **Identification and characterization of *in* *vivo* attenuated mutants of *Brucella* *melitensis***. *Mol Microbiol* 2000, **38**(3):543-551.

8. Zygmunt MS, Hagius SD, Walker JV, Elzer PH: **Identification of *Brucella* *melitensis* 16M genes required for bacterial survival in the caprine host**. *Microbes Infect* 2006, **8**(14-15):2849-2854.

9. Eskra L, Canavessi A, Carey M, Splitter G: ***Brucella* *abortus* genes identified following constitutive growth and macrophage infection**. *Infect Immun* 2001, **69**(12):7736-7742.

10. Kim S, Watarai M, Kondo Y, Erdenebaatar J, Makino S, Shirahata T: **Isolation and characterization of mini-Tn*5*Km2 insertion mutants of *Brucella* *abortus* deficient in internalization and intracellular growth in HeLa cells**. *Infect Immun* 2003, **71**(6):3020-3027.
