# Supplementary material for: Brucella melitensis VjbR and C12-HSL regulons: contributions of the N-dodecanoyl homoserine lactone signaling molecule and LuxR homologue VjbR to gene expression
Source: BMC Microbiol. 2010 Jun 8;10:167. doi: 10.1186/1471-2180-10-167 (PMC2898763; doi:10.1186/1471-2180-10-167)
Supplement: Additional file 4 — Table S4: Promoter(s) sequences and potential operons of downstream genes found to be altered by the deletion of vjbR and/or treatment of C12-HSL. Operons that are both found to be downstream of the predicted VjbR promoter sequence and altered by comparison of wild type and ΔvjbR, both with and without the addition of C12-HSL at exponential or stationary growth phases. [file 1471-2180-10-167-S4.DOCX]

TABLE S4. Promoter(s) sequences and potential operons of downstream genes found to be altered by the deletion of *vjbR* and/or treatment of C_12_-HSL.

| Promoter(s) Sequence [1] | Down-stream Loci | Gene Function | Exponential Growth Phase  Change (fold) | | | Stationary Growth Phase  Change (fold) | | |
| --- | --- | --- | --- | --- | --- | --- | --- | --- |
|  |  |  | ∆*vjbR*  / wt | wt+AHL / wt | ∆*vjbR*  / ∆*vjbR*+AHL | ∆*vjbR*  / wt | wt+AHL / wt | ∆*vjbR*  / ∆*vjbR*+AHL |
| **AACCGCCGTCTTCCTGAT** | I 0076 | Inorganic Pyrophosphatase | 1.5^⁪^ | - | - | - | - | - |
| GTCTTCCAGATAAAGGTT | I 0187 | DME Family Transporter | - | - | - | -1.8 | -2.2 | -4.0^⁪⁪^ |
| **ATGCGCCATGCCGAACAT** | I 0222 | Carbonic Anhydrase | - | -1.5^⁪^ | - | - | -1.5^⁪⁪^ | - |
| GTTCGCTTATTGCCGCAT  GATTTCCTCCTTCAGCAT | I 0243 | Hypothetical Protein | 1.6^⁪^ | 1.8^⁪^ | - | - | - | - |
| GGTATCCATTTTCGGGAT | I 0252 | ATP Synthase ε Subunit | 1.9^⁪^ | 2.7^⁪^ | 1.8^⁪^ | - | - | - |
| ATCCGCAGGAAAGCGCAT | I 0322 | 50S Ribosomal Protein L31 | - | 1.5^⁪^ | - | 1.8 | - | - |
| ATGCCCGAATTCGATCAT | I 0326 | Myo-Inositol-1(OR 4)-Monophosphatase | - | - | -2.3^⁪^ | - | 1.7^⁪^ | 4.1 |
|  | I 0327 | Protein Translation Elongation Factor P (EF-P) | -2.0^⁪^ | -2.6 | - | - | - | 1.6^⁪⁪^ |
| ATGCCCGAATTCGATCAT | I 0330 | OpgC Protein | - | -2.0 | -1.9^⁪⁪^ | - | - | - |
|  | I 0332 | Endodeoxyribonuclease RuvC | 1.5^⁪^ | - | - | - | - | 2.3 |
|  | I 0333 | Holiday Junction DNA Helicase, RuvA | - | -1.7 | -1.7^⁪⁪^ | - | - | 1.8^⁪⁪^ |
|  | I 0334 | Holliday Junction DNA Helicase, RuvB | - | -1.7 | - | - | - | 2.3^⁪⁪^ |
|  | I 0336 | TolQ Protein | 1.5^⁪^ | - | - | - | - | - |
|  | I 0337 | Biopolymer Transport Protein ExbD/TolR | 1.5^⁪^ | - | -2.5^⁪⁪^ | -1.5^⁪^ | - | - |
|  | I 0338 | TolA Protein | 1.5^⁪^ | - | - | - | - | - |
|  | I 0340 | Peptidoglycan-Associated Lipoprotein | - | - | -1.8^⁪⁪^ | -1.5^⁪^ | - | - |
| AACCGCTCTTTTGGGAAT | I 0379 | Phosphinothricin N-Acetyltransferase | - | 1.5^⁪^ | - | - | - | 2.4^⁪⁪^ |
| ATGATATTCATTAATCAT | I 0499 | Soluble Lytic Murein Transglycosylase | - | 2.4^⁪^ | - | 2.0 | 1.9 | -3.0^⁪⁪^ |
|  | I 0502 | Hypothetical Membrane Spanning Protein | - | -1.6^⁪^ | -2.3^⁪⁪^ | - | - | 1.7^⁪⁪^ |
| ATGATTAATGAATATCAT | I 0503 | Acyl-CoA Hydrolase | - | 1.6^⁪^ | - | - | - | - |
| ATGATGAAGTCCGATGCC | I 0534 | Hypothetical Protein | - | -1.6 | -2.1^⁪⁪^ | - | -2.2 | -9.8^⁪⁪^ |
| AACAGGAAATCGGCTACT | I 0535 | Hypothetical Protein | 1.9 | - | -4.3^⁪⁪^ | -1.5^⁪^ | - | - |
| ATTCGATGGATGGAGCAT | I 0587 | Lipoprotein, ComL | - | 1.8^⁪^ | 2.2^⁪⁪^ | - | - | - |
|  | I 0588 | DNA repair protein, RecN | - | 1.7^⁪^ | 2.2^⁪⁪^ | - | - | - |
|  | I 0589 | DNA Ligase | - | 1.8^⁪^ | 1.6^⁪⁪^ | - | - | - |
| ATTTGCCCATGCGAGCAT | I 0723 | Hypothetical Protein | - | -2.6 | -2.7^⁪⁪^ | - | - | - |
|  | I 0724 | Hypothetical Protein | - | - | -1.5^⁪⁪^ | -1.5^⁪^ | - | - |
|  | I 0726 | Protein GlpX | - | - | - | -1.6^⁪^ | - | - |
|  | I 0727 | D-Alanine--D-Alanine Ligase A | - | -1.8^⁪^ | -1.6^⁪⁪^ | 1.5^⁪^ | - | - |
|  | I 0728 | Single-Stranded-DNA-DNA-Specific Exonuclease, RecJ | - | - | -2.1^⁪⁪^ | -1.7 | - | - |
| ATCAGGAAAATAGCGATT | I 0808 | MerR Family Transcriptional Regulator | -2.1^⁪^ | -1.6^⁪^ | - | - | - | - |
| ATCCGTCATATCCATGAT | I 0826 | Ribosome Releasing Factor | 1.6^⁪^ | 2.3^⁪^ | - | - | - | - |
|  | I 0827 | Undecaprenyl Pyrophosphate Synthetase | 1.6^⁪^ | 2.7^⁪^ | - | -1.5 | - | 1.6^⁪⁪^ |
|  | I 0830 | Outer Membrane Protein, Omp89 | - | - | - | -1.6 | - | - |
|  | I 0831 | UDP-3-O-[3-hydroxymyristoyl] Glucosamine N-Acyltransferase | 1.6^⁪^ | 2.3 | 1.5^⁪⁪^ | - | - | - |
|  | I 0832 | (3R)-Hydroxymyristoyl ACP Dehydratase | - | - | - | -2.2^⁪^ | -1.5^⁪^ | - |
|  | I 0833 | UDP-N-Acetylglucosamine Acyltransferase | - | - | - | -1.5^⁪^ | -1.5^⁪^ | 1.7^⁪⁪^ |
|  | I 0834 | Hypothetical Protein | - | 1.7^⁪^ | - | - | - | - |
|  | I 0835 | Lipid-A-Disaccharide Synthase | - | -1.8 | - | - | - | 1.9^⁪⁪^ |
| ATCCCCTGACTGGCTCAT | I 0840 | LexA Repressor | - | 1.7^⁪^ | - | - | - | - |
|  | I 0842 | Molybdenum Cofactor Biosynthesis Protein C | - | 1.5^⁪^ | - | 1.7 | 1.6 | - |
|  | I 0843 | Indole-3-Glycerol Phosphate Synthase | 1.8 | 1.8 | - | - | - | - |
|  | I 0844 | Anthranilate Phosphoribosyltransferase | - | 1.6 | - | - | - | - |
| ATGAGCCAGTCAGGGGAT | I 0848 | Probable Carnitine Operon Oxidoreductase CaiA | - | 1.8^⁪^ | - | - | - | - |
| TTCAGCCCCATCGGGGAT | I 0852 | Methyltransferase | - | -2.1 | - | - | - | - |
|  | I 0853 | Septum Formation Initiator | 1.5^⁪^ | - | - | - | - | - |
|  | I 0856 | Dihydrolipoamide Acetyltransferase | -1.7^⁪^ | -1.8^⁪^ | - | - | - | - |
|  | I 0859 | Lipoyl Synthase | -1.6^⁪^ | -1.7^⁪^ | -1.9^⁪⁪^ | - | - | - |
| ATGCCCGATAGCGATGCG | I 0874 | Clp Protease Subunit | 1.6^⁪^ | - | -1.5^⁪⁪^ | - | - | - |
|  | I 0876 | ATP-Dependent Protease La | - | - | - | 1.5^⁪^ | - | - |
|  | I 0877 | DNA-Binding Protein HU-α | - | - | - | 1.5^⁪^ | - | - |
| ATGACCAAGAGCGCGGAT | I 0899 | Phage-Related DNA Binding Protein | -1.8 | -1.5^⁪^ | -1.9^⁪⁪^ | 1.6 | - | -2.4^⁪⁪^ |
| ATGCGGGAAATATATGAT | I 0945 | 6-Aminohexanoate-Dimer Hydrolase | -2.4 | -2.2 | - | - | - | - |
| **ATCATATATTTCCCGCAT** | I 0948 | Hypothetical Protein, VceC | 1.1^⁪^ | 1.4^⁪^ | - | 1.6^⁪^ | 1.3^⁪^ | - |
| AACCTCTCGATAACGCTT | I 0969 | NAD(+) Synthase | - | - | -1.7^⁪⁪^ | -1.7^⁪^ | - | - |
| ATCAGCGCGACCACGCAT | I 0971 | Phospho-2-Dehydro-3-Deoxyheptonate Aldolase | - | 2.5^⁪^ | - | - | - | - |
|  | I 0972 | Glutathione Reductase | - | 1.9^⁪^ | 2.2^⁪⁪^ | - | - | - |
| **ATGATCTATCTCGCAACC** | I 1020 | Molybdopterin-Guanine Dinucleotide Biosynthesis Protein A | - | - | - | -1.5^⁪^ | - | - |
|  | I 1021 | Molybdopterin-Guanine Dinucleotide Biosynthesis Protein B | 1.7^⁪^ | 2.5^⁪^ | - | -1.5 | - | - |
| ATGCTCTAAATAGAAAAT | I 1023 | Glutathione S-Transferase III | - | 1.8^⁪^ | 3.7^⁪⁪^ | - | - | - |
|  | I 1024 | 3-Hydroxyisobutyrate Dehydrogenase | -1.5^⁪^ | - | 1.7^⁪⁪^ | -1.5^⁪^ | -1.5^⁪^ | - |
| ATTAGAGGGTTTCGGCTT | I 1062 | Acetyl-CoA Carboxylase | 1.6^⁪^ | 1.5^⁪^ | - | - | - | - |
| AAGTTCAATTAAGCAGTT | I 1103 | NifR3-Like Protein | - | -1.8 | - | - | - | - |
| ATCATCGATCTGTCGGTC | I 1132 | ATPase of the PP Superfamily | - | -1.7^⁪^ | -2.5^⁪⁪^ | - | - | - |
|  | I 1133 | 30S Ribosomal Protein S4 | - | - | - | - | -1.5^⁪^ | - |
| AAGTGCTTATTGACTGAT | I 1167 | Putative Aromatic Compound Catabolism Protein | 1.8^⁪^ | 2.6 | 1.6^⁪⁪^ | - | - | - |
|  | I 1168 | 50S Ribosomal Protein L13 | - | 1.7^⁪^ | 1.8^⁪⁪^ | - | - | 1.7^⁪⁪^ |
|  | I 1169 | 30S Ribosomal Protein S9 | - | 2.2^⁪^ | 2.4^⁪⁪^ | -1.5^⁪^ | - | 1.6^⁪⁪^ |
|  | I 1170 | Agmatinase | - | 2.3^⁪^ | - | -1.5^⁪^ | - | 1.6^⁪⁪^ |
|  | I 1171 | N-Acetyl-γ-Glutamyl-Phosphate Reductase | 1.8^⁪^ | 2.4 | - | - | - | 1.7^⁪⁪^ |
| ATGGCCGATATCTCTGTA | I 1293 | Coproporphyrinogen III Oxidase | -1.6^⁪^ | -2.1 | -1.9^⁪⁪^ | - | - | - |
|  | I 1295 | Orotate Phosphoribosyltransferase | 1.5^⁪^ | 1.5^⁪^ | - | - | - | - |
| ATCTGGTTTAACGTGCAT | I 1314 | Hypothetical Protein | 1.6^⁪^ | 2.0 | - | - | - | - |
| **ATGAGGCAAATCTCGCCT** | I 1351 | Penicillin-Binding Protein 1A | 1.8^⁪^ | - | -3.7^⁪⁪^ | - | -1.5^⁪^ | -1.9^⁪⁪^ |
| CTGAGCCATATGGCGGTT  GGTTGCGAGCTTCAGGAT | I 1360 | Glutamyl-tRNA(GLN) Amidotransferase Subunit A, Amidase | - | 2.6^⁪^ | - | -2.3 | -1.8^⁪^ | 1.6^⁪⁪^ |
|  | I 1361 | Hypothetical Cytosolic Protein | -1.8 | - | 1.6^⁪⁪^ | -1.7^⁪^ | -1.7^⁪^ | - |
| ATCCTGCATGCCGCTAAC | I 1470 | YicC Protein | -1.8 | - | - | - | - | 1.6^⁪⁪^ |
|  | I 1474 | Hypothetical Protein | -1.7^⁪^ | -1.6^⁪^ | - | - | - | - |
| ATGCTCTAAATGGAAAAT | I 1609 | Hypothetical Protein | - | 1.5^⁪^ | -2.2^⁪⁪^ | - | - | - |
| ATTTTCCATTTAGAGCAT | I 1611 | Dihydroorotate Dehydrogenase | - | 1.8^⁪^ | - | -1.6 | - | 3.1^⁪⁪^ |
| TTCAGCGATACATCAGAT | I 1613 | Hypothetical Protein | - | 1.6^⁪^ | - | - |  | - |
| GTTTTCTTTATGCCTGTT | I 1614 | Hypothetical Protein | -1.6^⁪^ | -1.8^⁪^ | - | - | - | - |
| AACAGGCATAAAGAAAAC | I 1618 | Protein ApaG | -1.9^⁪^ | -1.6^⁪^ | - | - | - | - |
| ATCTTCGTTATCCGGAAT | I 1623 | Hypothetical Protein | - | 1.6^⁪^ | - | - | - | - |
| AGCTTTCATAATGCTGAT | I 1658 | Hypothetical Protein | - | - | - | 1.5 | - | - |
| ATGTGCGGGATGACTCTT | I 1747 | Aldehyde Dehydrogenase | - | 2.1^⁪^ | - | - | - | - |
| ACGAGTCATAAAACTGAT | I 1824 | ATP-Dependent RNA Helicase, DeaD | -1.8^⁪^ | - | - | - | - | 1.7^⁪⁪^ |
|  | I 1826 | Hypothetical Protein | -1.8 | -1.5^⁪^ | - | -1.5^⁪^ | - | 2.0^⁪⁪^ |
| ATCAGTTTTATGACTCGT | I 1833 | Fructokinase | - | - | - | -1.5^⁪^ | - | - |
|  | I 1834 | Ubiquinone/Menaquinone Biosynthesis Methyltransferase, UbiE | 1.7^⁪^ | - | -2.0^⁪⁪^ | -1.9 | -1.5^⁪^ | 2.3^⁪⁪^ |
| **ATCCGTGATCTCGCAGCT** | I 1836 | Nicotinate Phosphoribosyltransferase | - | - | - | - | -1.5^⁪^ | - |
|  | I 1837 | Cellobiose phosphorylase | 1.5^⁪^ | - | - | - | - | 2.1^⁪⁪^ |
| ATCTGCGCATTGGCAGAT | I 1844 | Hypothetical Protein | - | 1.5^⁪^ | 3.5 | - | - | - |
| AGTTGCGATTTAGCTCTT | I 1920 | Hypothetical Protein | -1.7 | - | - | - | - | - |
|  | I 1928 | Enoyl-CoA Hydratase | - | -1.5^⁪^ | - | - | - | - |
| AACAGCCCGTTCAATCTT | I 1954 | ABC-Type Transport System Metal Ion Substrate Binding Protein | -2.0 | -1.6 | - | 2.0 | 2.1 | - |
|  | I 1955 | Iron-Responsive Regulator Irr | - | -1.8^⁪^ | - | - | - | - |
| AAGATTGAACGGGCTGTT  AACCGCGAAGACGAGGCT | I 1956 | 3-Hydroxydecanoyl-ACP Dehydratase | - | 1.6^⁪^ | - | 1.5^⁪^ | - | - |
| GATCGCCATTTGCCGGAT | I 1980 | DNA-Binding Ferritin-Like Protein | 1.9^⁪^ | - | -1.8^⁪^ | - | - | - |
| ATGCTCTAAAAAGATACC | I 2039 | Pantothenate Kinase | -2.1 | -1.9 | - | - | - | 1.9^⁪^ |
| **ATGACCGATATCGCTGAT** | II 0025 | Attachment Mediating Protein VirB1 | -2.2 | -1.9 | - | -2.6 | -2.2 | - |
| **ATGCTCCAGATCGCAGAT** | II 0026 | Attachment Mediating Protein VirB2 | - | -2.1 | - | -4.3 | -3.6 | - |
|  | II 0027 | Channel Protein VirB3 | - | - | - | -3.9 | -3.2 | - |
|  | II 0029 | Attachment Mediating Protein VirB5 | -2.0 | - | 1.7^⁪^ | -5.7 | -4.5 | - |
|  | II 0030 | Channel Protein VirB6 | - | - | -1.6^⁪^ | -2.8 | -2.3 | - |
|  | II 0032 | Channel Protein VirB8 | -1.6^⁪^ | - | - | -3.3 | -2.6 | - |
|  | II 0033 | Channel Protein VirB9 | - | - | - | -1.8 | -1.9 | - |
|  | II 0034 | Channel Protein VirB10 | - | -1.5 | - | -2.0 | -1.9 | - |
|  | II 0035 | ATPase Protein VirB11 | - | -1.6^⁪^ | - | -1.6^⁪^ | -1.7^⁪^ | - |
|  | II 0036 | OMP, OprF*,* VirB12 | - | - | - | -1.7 | -1.7 | - |
| AAGACCTATAGAGCGGTT | II 0037 | Hypothetical Cytosolic Protein | - | 1.8^⁪^ | - | - | - | - |
|  | II 0038 | D-Serine, D-Alanine, Glycine Transporter | - | -1.5^⁪^ | - | -1.6^⁪^ | -1.8 | - |
| AGGTTCGCACTGCCGGAT | II 0051 | LuxR-like, Nodulation Protein W | -1.9 | -2.8 | - | - | - | - |
| AGCCGCCTTTTTCATCAT  AGTCTATATTTGAAGCAT | II 0120 | High-Affinity Branched-Chain AA Transport System, LivM/LivG | 1.9^⁪^ | - | -3.1^⁪^ | - | - | - |
|  | II 0124 | Aldehyde Dehydrogenase | - | 1.8^⁪^ | - | -1.5^⁪^ | -1.5^⁪^ | - |
|  | II 0125 | Aldehyde Dehydrogenase | - | 2.1^⁪^ | - | - | - | - |
|  | II 0126 | Amino Acid Permease | - | 2.3^⁪^ | 2.3^⁪^ | - | - | 1.5^⁪^ |
| ATGCTTCAAATATAGACT | II 0127 | IclR Family Acetate Operon Repressor | - | 1.6^⁪^ | - | -1.8 | - | 1.6^⁪^ |
|  | II 0128 | Hypothetical Transcriptional Regulator | - | 2.4^⁪^ | - | - | -1.5^⁪^ | - |
|  | II 0129 | Hydrolase | - | - | - | -1.6^⁪^ | - | 1.8^⁪^ |
|  | II 0130 | Adenosylmethionine-8-Amino-7-Oxononanoate Aminotransferase | -1.8^⁪^ | - | 1.8^⁪^ | - | - | 2.5^⁪^ |
|  | II 0131 | 4-Aminobutyrate Aminotransferase | - | 1.7^⁪^ | - | - |  | 2.0^⁪^ |
| **ATGCTTAAGGTGGAAATT** | II 0151 | Flagellar M-Ring Protein, FliF | -2.2 | -2.1 | - | 2.1^⁪^ | - | - |
| ATCATTTAGCCCGCTATT | II 0152 | Flagellar M-Ring Protein, FliF | - | 1.7^⁪^ |  | - | - | - |
|  | II 0153 | Hypothetical Protein | -1.6^⁪^ | -1.7 |  | -1.6 | - | - |
|  | II 0154 | Flagellar Motor Protein | -1.5^⁪^ | - | 1.5^⁪^ | - | - | - |
|  | II 0155 | Chemotaxis MotC Protein Precursor | -1.7 | - | - | - | - | - |
|  | II 0157 | Soluble Lytic Murein Transglycosylase | -1.9 | -2.1 | - | -1.5^⁪^ | -2.1 | - |
|  | II 0159 | Flagellar Hook Protein, FlgE | -1.5^⁪^ | - | - | - | - | - |
|  | II 0160 | Flagellar Hook-Associated Protein, FlgK | -1.6^⁪^ | -2.0 | -1.7^⁪^ | - | - | - |
|  | II 0161 | Flagellar Hook-Associated Protein 3 | -1.8^⁪^ | -2.7 | - | - | - | - |
|  | II 0162 | FlaF Protein | -2.1 | -2.0^⁪^ | - | - | - | -1.6^⁪^ |
|  | II 0164 | Flagellar Basal-Body Rod Modification Protein, FlgD | - | - | -1.7^⁪^ | -1.5^⁪^ | - | - |
|  | II 0165 | Flagellar Biosynthesis Protein | -1.9^⁪^ | -2.8 | - | - | - | - |
|  | II 0166 | Flagellar Biosynthesis Protein, FlhA | - | - | -2.6^⁪^ | -1.6^⁪^ | - | - |
|  | II 0167 | Flagellar Biosynthesis Protein, FlhA | -1.6^⁪^ | -2.3 | - | -1.5^⁪^ | -1.9^⁪^ | -5.5^⁪^ |
|  | II 0174 | Hypothetical Protein | - | -1.7^⁪^ | - | - | - | - |
| **ATCAGCCATATCGATAAC** | II 0187 | Hypothetical Cytosolic Protein | -2.0 | - |  | -1.8^⁪^ | -1.9^⁪^ | - |
|  | II 0188 | Hypothetical Cytosolic Protein | - | - |  | 1.6^⁪^ | - | - |
| AACCGTGAAAAAGAGAAT | II 0218 | Dihydrolipoamide Acetyltransferase | - | -2.0 | -1.5^⁪^ | -1.5^⁪^ | - | - |
|  | II 0219 | IclR Family Transcriptional Regulator | -3.2 | -5.8 | -3.1^⁪^ | -1.5^⁪^ | - | - |
| ATTCTCTTTTTCACGGTT | II 0221 | Dipeptide Transport System, DppC | - | -1.7^⁪^ | -2.8^⁪^ | -1.5^⁪^ | - | - |
|  | II 0222 | Oligopeptide Transport System, OppB | - | -1.5^⁪^ | - | - | - | - |
|  | II 0223 | Oligopeptide Transport System, OppF | -1.7^⁪^ | - | - | - | - | - |
|  | II 0224 | Formyl-Coenzyme A Transferase | - | 1.8^⁪^ | - | -2.0^⁪^ | - | - |
|  | II 0225 | 6-Oxohexanoate Dehydrogenase | -3.8^⁪^ | -2.1^⁪^ | - | - | -1.6^⁪^ | - |
| AACCCCCATACAGAAGCC | II 0245 | Universal Stress Protein Family, UspA | -1.8 | -1.7 | -2.0^⁪^ | -2.5 | -2.5 | - |
| ATTTGCCATTTGAACCAT | II 0314 | Anthranilate Synthase Component II | - | -1.5^⁪^ | -1.8^⁪^ | - | - | - |
| ATGGTTCAAATGGCAAAT | II 0316 | 2-Deoxy-D-Gluconate 3-Dehydrogenase | - | -1.5^⁪^ | - | - | - | - |
|  | II 0318 | 6-Aminohexanoate-Dimer Hydrolase | 1.5^⁪^ | 1.5^⁪^ | -1.8^⁪^ | - | - | -2.8^⁪^ |
|  | II 0319 | 6-Aminohexanoate-Dimer Hydrolase | - | 2.0 | 2.1^⁪^ | - | - | -2.9^⁪^ |
|  | II 0321 | Hypothetical Protein | - | 1.5^⁪^ | - | - | - | - |
|  | II 0322 | Cytochrome C Oxidase, Subunit IV | - | - | - | -1.5^⁪^ | -1.6^⁪^ | - |
|  | II 0323 | NAD(P) Transhydrogenase α Subunit | - | 2.4^⁪⁪^ | 1.5^⁪^ | - | - | - |
|  | II 0324 | NAD(P) Transhydrogenase α Subunit | 1.6^⁪⁪^ | - | - | - | - | 2.3^⁪^ |
| AAGCGAGAAAACGCTCAT | II 0369 | Atrazine Chlorohydrolase | - | 1.9 | - | - | - | - |
|  | II 0370 | GntR Family, Histidine Utilization Repressor | -1.8 | - | - | - | - | - |
| AAGAGCGCGGGCGCTGAT | II 0373 | D-Amino Acid Dehydrogenase Small Subunit | - | 1.9^⁪^ | - | - | - | 2.1^⁪^ |
|  | II 0374 | Alanine Racemase, Catabolic | -2.2 | - | 3.8^⁪^ | - | - | - |
| ATCAGCGCCCGCGCTCTT | II 0375 | AsnC Family Transcriptional Regulator | - | - | - | 1.5^⁪^ | 1.5^⁪^ | - |
| AAAAGCGTTTTGGCTGAT | II 0387 | Formyltetrahydrofolate Deformylase | -1.6^⁪^ | - | - | - | - | - |
| **ATCAGCGGTTTTGCGGTT** | II 0390 | LysR Family Transcriptional Regulator | -2.0^⁪^ | - | - | - | - | - |
| AGTTTCGCGCTGCCTCTT | II 0420 | Thymidylate Synthase | -1.8 | - | - | -1.5^⁪^ | - | - |
|  | II 0421 | Hypothetical Protein | 1.8^⁪^ | - | - | 2.0^⁪^ | - | - |
|  | II 0422 | Fructose-1,6-Bisphosphatase | -2.4 | - | 1.8^⁪^ | - | - | - |
|  | II 0423 | Fructose-Bisphosphate Aldolase | - | - | - | 1.8^⁪^ | - | - |
| TTGCGCGATGTAGCTGTT | II 0492 | Acyl-CoA Dehydrogenase | - | 2.0^⁪^ | - | - | - | -1.7^⁪^ |
| ATGCTGCAACTGGCAGAC | II 0512 | 6-Phosphogluconolactonase | - | 1.7^⁪^ | 2.0^⁪^ | -1.6^⁪^ | - | - |
| ATGTGTGCATTTGCGGTT | II 0513 | Glucose-6-Phosphate 1-Dehydrogenase | - | 1.7^⁪^ | 2.8^⁪^ | -1.6^⁪^ | - | - |
| ATGCGTTAAAGCGCGCCT | II 0559 | Aminomethyltransferase | -1.5^⁪^ | - | - | - | - | - |
|  | II 0560 | Glycine Cleavage System H Protein | - | 1.7^⁪^ | - | - | - | 1.5^⁪^ |
| ATTTGCCTTTTGCGGGTT | II 0621 | SN-Glycerol-3-Phosphate Transport Protein UgpC | - | 2.0^⁪^ | - | - | - | - |
|  | II 0622 | SN-Glycerol-3-Phosphate Transport Protein UgpE | - | - | - | - | -1.6^⁪^ | - |
|  | II 0624 | SN-Glycerol-3-Phosphate Transport Protein UgpA | - | - | - | - | -1.5^⁪^ | 1.7^⁪^ |
| AACCCGCAAAAGGCAAAT | II 0627 | Probable Adenine Deaminase | -1.9 | - | - | 1.6 | - | - |
| AGGGCCGCTATAGCCGAT | II 0655 | Alkaline Phosphatase | - | 2.1 | -15.8^⁪^ | -3.2 | - | - |
| GACGCCGATAAAGTTGAA | II 0675 | Glutamyl-tRNA Amidotransferase Subunit A | - | 1.8^⁪^ | - | 1.5 | - | - |
| ATTCGCTGCCTGACGGTT | II 0695 | Phosphatidylcholine Synthase | 1.5^⁪^ | - | - | - | - | - |
|  | II 0696 | Quinone Reductase | - | - | - | 1.5^⁪^ | - | - |
|  | II 0699 | Galactoside Transport System Permease Protein, MglC | 1.6^⁪^ | - | -2.3^⁪^ | -1.8^⁪^ | - | 2.6^⁪^ |
|  | II 0700 | Galactoside Transport System Permease Protein, MglC | 1.6^⁪^ | - | -1.8^⁪^ | -2.1 | - | 5.6^⁪^ |
|  | II 0701 | Ribose Transport System, RbsC | 2.4^⁪^ | 2.2 | - | - | - | 2.6^⁪^ |
|  | II 0702 | ABC-Type Transport System Surface Lipoprotein, Simple Sugar Transport System Periplasmic Binding Protein | 1.5^⁪^ | - | -3.6^⁪^ | - | -2.8 | -5.1^⁪^ |
| AACATTGAAACATATGAT | II 0759 | Two Component Response Regulator DivK | -2.1^⁪^ | -1.5^⁪^ | 1.8^⁪^ | - | - | - |
|  | II 0760 | Two Component Response Regulator | - | 1.6^⁪^ | - | - | - | - |
|  | II 0762 | MFS Superfamily Transporter | - | 2.8^⁪^ | - | - | - | - |
|  | II 0770 | Potassium Efflux System Protein, PhaA, PhaB | -2.0^⁪^ | -2.1 | -1.6^⁪^ | - | - | - |
| AGCTTCTGGATCGAGGTT | II 0810 | ArsR Family Transcriptional Regulator | - | 2.0 | - | 1.8 | 1.6^⁪^ | -2.6^⁪^ |
| AACCTCGATCCAGAAGCT | II 0811 | Hypothetical Protein | -1.6^⁪^ | - | - | - | - | - |
| TTCTTCTGTTTGAAGCAT | II 0812 | Peptide Deformylase | -1.6^⁪^ | - | - | 1.8 | 1.5^⁪^ | - |
| ATGCTTCAAATAGAAGAG | II 0880 | Acetate Kinase | 1.5 | - | - | - | - | - |
| GATTTCGCCATCGAGGTT | II 0934 | Nickel Resistance Protein | -1.7^⁪^ | -1.5^⁪^ | - | - | - | - |
| ATCTGATTTTTACATGTT | II 0938 | Myo-Inositol 2-Dehydrogenase | 1.6^⁪^ | 1.5^⁪^ | - | - | - | 1.5^⁪^ |
|  | II 0939 | ThuA Protein | - | -2.0 | -2.8^⁪^ | - | - | - |
|  | II 0940 | Maltose/Maltodextrin ABC-Type Transport System Protein, MalK | 1.7^⁪^ | - | -4.2^⁪^ | - | - | - |
|  | II 0941 | Maltose/Maltodextrin ABC-Type Transport System Protein, MalK | - | - | - | -2.0 | - | 2.0^⁪^ |
|  | II 0943 | Maltose/Maltodextrin ABC-Type Transport System Protein, MalF | - | -1.7^⁪^ | 1.6^⁪^ | - | - | - |
|  | II 0945 | Maltose/Maltodextrin ABC-Type Transport System Protein, MalE | -1.5^⁪^ | 1.5^⁪^ | 2.9 | - | - | - |
| AAGATCAATATCTCGAAT | II 0947 | FtrB Transcriptional Regulator | - | 1.6^⁪^ | - | - | - | - |
| AGGAGTAAAACCGAAGAT | II 0980 | Ribitol 2-dehydrogenase | - | -2.1 | - | - | - | 1.6^⁪^ |
| TGCAGCCATATCTTGTTT | II 1028 | Tetraacyldisaccharide 4'-Kinase | - | -1.5^⁪^ | -1.6^⁪^ | - | -1.5^⁪^ | - |
|  | II 1030 | Putative Lipoprotein | - | 2.0^⁪^ | 1.8^⁪^ | - | - | 1.8^⁪^ |
|  | II 1033 | Protein PmbA | - | 1.5^⁪^ | - | - | - | - |
| ATCCGGCATATCTCTCAC | II 1069 | Adhesin, AidA | -1.5^⁪^ | - | - | -1.5 | - | - |
| TTTAGCAATATCGAGGAT | II 1074 | Citrate Lyase β Subunit | 1.6^⁪^ | - | - | - | - | - |
|  | II 1075 | Hypothetical Cytosolic Protein | 1.7^⁪^ | - | -2.3^⁪^ | -1.6^⁪^ | -1.6^⁪^ | 1.5^⁪^ |
| **ATCAGCTTTATCAACGGAT** | II 1116 | LuxR Family Transcriptional Regulator, VjbR | -2.3 | - | - | -1.9 | - | - |
|  | II 1117 | TetR Family Transcriptional Regulator | -1.9^⁪^ | -1.5^⁪^ | - | - | - | - |
| ATGCGCGTTATCGTTGAA | II 1120 | ABC-Type Fe^3+^ Transport System | - | 1.7^⁪^ | - | - | - | - |
|  | II 1121 | ABC-Type Fe^3+^ Transport System Permease Protein, SfuB | - | - | - | -1.8^⁪^ | -1.9 | - |
| AACCGCCAGATCGAGGAC | II 1133 | Ornithine Decarboxylase | - | - | - | -2.4 | -1.8 | - |
| GTCCTCGATCTGGCGGTT | II 1134 | Amidase | - | - | - | -1.5^⁪^ | - | - |
|  | II 1136 | Hypothetical Protein | -1.5^⁪^ | -1.8^⁪^ | -2.1^⁪^ | - | - | - |
|  | II 1137 | Hypothetical Protein | - | 1.6^⁪^ | - | - | - | - |
|  | II 1138 | Hypothetical Protein | - | -1.5^⁪^ | -4.6^⁪^ | -1.5^⁪^ | -2.3^⁪^ | -3.9^⁪^ |

Promoter sequences listed in bold were confirmed to be activated by VjbR, and the downstream genes in potential operons are separated by a dashed line and alternating grey shading [1]. A (-) indicates genes excluded for technical reasons or had a fold change of less than 1.5; ^⁪^ genes that did not pass the statistical significance test but showed an average alteration of at least 1.5-fold. Fold change values are the averaged log_2_ ratio of the normalized signal values from two independent statistical analyses. Abbreviations as follows: DME, Drug/Metabolite Exporter; ACP, Acyl-Carrier Protein.

**Reference**

1. de Jong MF, Sun YH, den Hartigh AB, van Dijl JM, Tsolis RM: **Identification of VceA and VceC, two members of the VjbR regulon that are translocated into macrophages by the *Brucella* type IV secretion system**. *Mol Microbiol* 2008, **70**(6):1378-1396.
