# Supplementary material for: Brucella melitensis VjbR and C12-HSL regulons: contributions of the N-dodecanoyl homoserine lactone signaling molecule and LuxR homologue VjbR to gene expression
Source: BMC Microbiol. 2010 Jun 8;10:167. doi: 10.1186/1471-2180-10-167 (PMC2898763; doi:10.1186/1471-2180-10-167)
Supplement: Additional file 5 — Table S5: Genetic loci identified with significant alterations in transcript levels between B. melitensis 16MΔvjbR and 16MΔvjbR with the addition of C12-HSL. Altered gene transcripts uniquely identified by the treatment of C12-HSL to the B. melitensis 16MΔvjbR background. [file 1471-2180-10-167-S5.DOCX]

TABLE S5. Genetic loci identified with significant alterations in transcript levels between *B. melitensis* 16MΔ*vjbR* and 16MΔ*vjbR* with the addition of C_12_-HSL.

| BME Loci | Gene Function | ∆*vjbR*+AHL / ∆*vjbR*  Change (fold) | | Δ*babR* vs. wt Change Identified | STM |
| --- | --- | --- | --- | --- | --- |
|  |  | Exp | Stat |  |  |
| **Amino Acid Transport and Metabolism** | | | | | |
| I 0110 | Agmatinase | 2.8 | - |  |  |
| I 0231 | NAD-Specific Glutamate Dehydrogenase | - | 4.0 | [1] |  |
| I 0414 | ABC-Type Spermidine/Putrescine Transport System Permease Protein PotI | - | 2.3 |  |  |
| I 0706 | CobC Protein | 2.2^⁪^ | 2.8 |  |  |
| I 1309 | Histidinol-Phosphate Aminotransferase | 4.4 | - |  |  |
| I 1638 | Glutamate Synthase (NADPH) Small Chain | - | 2.2 | [1] |  |
| I 1848 | Dihydroxy-Acid Dehydratase | 2.6 | 1.9^⁪^ |  | [2] |
| I 1869 | Homoserine Lactone Efflux Protein LysE | -3.1^⁪^ | 2.1^⁪^ | [3] |  |
| II 0506 | ABC-Type Oligopeptide Transport System Permease Protein OppC | -1.8 | 2.7^⁪^ |  |  |
| II 0554 | Glutamine Synthetase | -2.9 | - |  |  |
| **Carbohydrate Transport and Metabolism** | | | | | |
| I 0326 | Myo-Inositol-1(Or 4)-Monophosphatase | -2.3^⁪^ | 4.1 |  |  |
| II 0512 | 6-Phosphogluconolactonase | 2.6 | - |  |  |
| II 0544 | ABC-Type Sn-Glycerol-3-Phosphate Transport ATP-Binding Protein UgpC | - | 3.7 |  |  |
| II 0753 | ABC-Type Sorbitol/Mannitol Transport Inner Membrane Protein | 2.5 | 1.5^⁪^ |  |  |
| II 0755 | ABC-Type Sugar-Binding Transport System Protein | 2.4 | - |  |  |
| II 0942 | ABC-Type Maltose Transport System Permease Protein MalG | -1.6^⁪^ | 2.1 |  |  |
| II 0945 | ABC-Type Maltose-Binding Periplasmic Protein Transport System | 2.9 | - |  |  |
| **Cell Motility** | | | | | |
| II 0154 | Flagellar Motor Protein MotB | -1.5^⁪^ | - | [3] | [4] |
| II 0156 | Chemotaxis Protein MotD | -1.5^⁪^ | - | [3] |  |
| II 0170 | Flagellar Protein FlgJ | -1.4^⁪^ | - | [3] |  |
| II 1114 | Flagellar Biosynthetic Protein FlhB | - | 3.0^⁪^ | [3] |  |
| **Cell Wall, Membrane and Envelope Biogenesis** | | | | | |
| I 0500 | Soluble Lytic Murein Transglycosylase | 1.2^⁪^ | - | [3] |  |
| I 1831 | Penicillin-Binding Protein 1A | - | 3.0 |  |  |
| I 1878 | Soluble Lytic Murein Transglycosylase | - | 3.3 |  |  |
| II 0384 | Glucosamine-6-Phosphate Isomerase | - | 2.8 |  |  |
| II 0685 | Glucosamine Fuctose 6-Phosphate Aminotransferase | -1.8^⁪^ | - | [3] |  |
| II 0832 | UDP Glucose-4-Epimerase | -1.6^⁪^ | - | [3] |  |
| II 0847 | Glycosyl Transferase | - | -1.1^⁪^ | [3] |  |
| II 0848 | GDP Mannose 4,6-Dehydratase | 2.3^⁪^ | - | [3] |  |
| II 0849 | GDP 4-Dehydro-D-Rhamnosereductase | 1.2^⁪^ | - | [3] |  |
| **Coenzyme Transport and Metabolism** | | | | | |
| I 0001 | Uroporphyrinogen Decarboxylase | - | 1.6 |  |  |
| I 2029 | S-Adenosyl-L-Homocysteine Hydrolase | -1.2^⁪^ | 1.1^⁪^ | [3] |  |
| II 0096 | Coproporphyrinogen III Oxidase | -2.1^⁪^ | - | [3] |  |
| II 0130 | Adenosylmethionine-8-Amino-7-Oxononanoate Aminotransferase | 1.8^⁪^ | 2.9 |  |  |
| II 0678 | Lipoate-Protein Ligase B | 2.3 | - |  |  |
| **Defense Mechanisms** | | | | | |
| I 0323 | ABC-Type Multidrug Transport ATP-Binding Protein MsbA | - | 3.3 |  |  |
| **Energy Production and Conversion** | | | | | |
| I 0137 | Malate Dehydrogenase | - | 1.8 |  |  |
| I 0380 | Malate Synthase G | - | 2.6 |  |  |
| II 0564 | Proline Dehydrogenase / Delta-1-Pyrroline-5-Carboxylate Dehydrogenase | 1.8^⁪^ | - | [3] |  |
| **General Function Prediction Only** | | | | | |
| I 0006 | Predicted GTPase | 5.9 | - |  |  |
| I 0196 | Protein ErfK/SrkK | 2.6 | - |  |  |
| I 0319 | BioY Protein | - | 2.4 |  |  |
| I 0614 | NAD(FAD)-Utilizing Dehydrogenases | - | 1.8 |  |  |
| I 0631 | Predicted Flavin-Nucleotide-Binding Protein | -1.9 | - |  |  |
| I 0694 | CobW Protein | - | 2.5 |  |  |
| I 1110 | Secretion Activator Protein | -1.9 | - |  |  |
| I 1458 | Homoserine Kinase | -1.3^⁪^ | 1.2^⁪^ | [3] |  |
| I 1637 | CoxG Protein | -2.9 | - |  |  |
| I 1894 | Gramicidin S Biosynthesis GrsT Protein | - | 3.3 |  | [5] |
| I 1951 | Putative Hydrolase | 2.6 | - |  |  |
| I 1969 | SAM-Dependent Methyltransferase | -1.7^⁪^ | 2.3 |  |  |
| II 0462 | ATP-Dependent Helicase | -3.8 | - |  |  |
| II 0771 | Hydroxyacylglutathione Hydrolase | -1.6^⁪^ | 1.9 |  |  |
| II 0828 | Possible S-Adenosylmethionine-Dependent Methyltransferase | -2.3 | - |  |  |
| II 0997 | NorQ Protein | -1.6^⁪^ | 3.0 |  | Ficht, u.p. |
| **Inorganic Ion Transport and Metabolism** | | | | | |
| II 0490 | ABC-Type Nickel Transport ATP-Binding Protein NikD | - | 2.7 |  |  |
| **Intracellular Trafficking, Secretion and Vesicular Transport** | | | | | |
| I 0131 | Signal Recognition Particle Receptor FtsY | - | 2.1 |  |  |
| **Lipid Transport and Metabolism** | | | | | |
| I 0026 | Oxoacyl Acyl Carrier Protein Reductase | - | 1.3^⁪^ | [3] |  |
| I 1553 | Bacteroid Development Protein BacA | - | 1.6^⁪^ | [3] | [6] |
| **Nucleotide Transport and Metabolism** | | | | | |
| I 0332 | Endodeoxyribonuclease RuvC | - | 2.3 |  |  |
| I 1558 | Ada Regulatory Protein / O-6-Methylguanine-Dna-Alkyltransferase | 3.8 | - |  |  |
| I 1801 | DNA Mismatch Repair Protein MutS | 1.6^⁪^ | 1.6 |  |  |
| **Posttranslational Modification, Protein Turnover and Chaperones** | | | | | |
| I 0047 | Molecular Chaperone, DNAJ Family | -3.1 | -1.6^⁪^ |  |  |
| II 0409 | Osmotically Inducible Protein C | -2.4^⁪^ | - | [3] |  |
| **Replication, Recombination and Repair** | | | | | |
| I 0246 | Primosomal Protein N | 2.9 | 1.8^⁪^ |  |  |
| I 1411 | Transposase | 2.6 | 1.7^⁪^ |  |  |
| **Secondary Metabolites Biosynthesis, Transport and Catabolism** | | | | | |
| I 0965 | ABC-Type Toluene Tolerance Protein Ttg2B | -3.6 | -2.4^⁪^ |  |  |
| **Signal Transduction Mechanisms** | | | | | |
| I 0374 | Sensory Transduction Histidine Kinase | -2.5^⁪^ | 1.8^⁪^ | [3] |  |
| I 1975 | PhoH Protein | - | 1.8 |  |  |
| II 1015 | Two-Component System Sensor | -1.5^⁪^ | - | [3] |  |
| **Transcription** | | | | | |
| I 0280 | RNA Polymerase Sigma-32 Factor | - | 2.0 |  |  |
| I 0744 | Transcription Antitermination Protein NusG | -2.4 | - |  |  |
| I 1573 | Transcriptional Regulator, LysR Family | -2.1^⁪^ | - | [3] | [7] |
| I 1750 | Glycerol-3-Phosphate Regulon Repressor | 3.1 | - |  |  |
| I 1913 | Transcriptional Regulator, LysR Family | -2.0^⁪^ | - | [3] | [7] |
| II 0104 | Transcriptional Regulator, AraC Family | -1.6^⁪^ | -1.9^⁪^ | [3] |  |
| II 0204 | Transcriptional Regulator, GntR Family | - | -2.1^⁪^ | [3] |  |
| II 0392 | Transcription Accessory Protein (S1 RNA Binding Domain) | - | 2.2 |  |  |
| II 0545 | Transcriptional Regulator, RpiR Family | - | 1.2^⁪^ | [3] |  |
| II 0688 | Transcription-Repair Coupling Factor | - | 2.0 |  | Ficht, u.p. |
| II 0814 | Transcriptional Regulator, AraC Family | -1.3^⁪^ |  | [3] |  |
| II 0878 | Transcriptional Regulator, GntR Family | - | 2.2^⁪^ | [3] |  |
| **Translation, Ribosomal Structure and Biogenesis** | | | | | |
| I 0132 | Fe-S Oxidoreductase | - | 1.8 |  |  |
| I 0377 | Ribosomal Large Subunit Pseudouridine Synthase D | - | 3.0 |  |  |
| I 0774 | Ssu Ribosomal Protein S5P | - | 2.2 |  |  |
| I 1529 | Glycyl-tRNA Synthetase β Chain | - | 2.5 |  |  |
| **Unknown and Other** | | | | | |
| I 0142 | Hypothetical Membrane Spanning Protein | -2.3 | - |  |  |
| I 0367 | Hypothetical Protein | - | 2.0 |  |  |
| I 0376 | Hypothetical Protein | -1.8^⁪^ | 4.0 |  |  |
| I 0484 | Hypothetical Protein | -1.5^⁪^ | 1.8 |  |  |
| I 0710 | Hypothetical Protein | - | 2.3 |  |  |
| I 0809 | Predicted Membrane Protein | -1.6^⁪^ | - |  |  |
| I 0813 | Hypothetical Protein | - | 2.8 |  |  |
| I 0912 | Hypothetical Protein | -1.7^⁪^ | - | [3] |  |
| I 1425 | Hypothetical Protein | -1.8^⁪^ | - |  |  |
| I 1539 | Hypothetical Protein | - | 2.1 |  | Ficht, u.p. |
| I 1783 | Hypothetical Membrane Spanning Protein | - | 2.9 |  |  |
| I 1844 | Hypothetical Protein | 3.5 | - |  | [8] |
| II 0464 | Hypothetical Membrane Associated Protein | 1.8^⁪^ | 1.9 |  |  |
| II 0809 | Hypothetical Membrane Spanning Protein | - | 2.0 |  |  |
| II 1025 | Hypothetical Protein | -1.7^⁪^ | 2.3 |  |  |
| II 1050 | Hypothetical Protein | 2.6 | - |  |  |

A (-) indicates genes excluded for technical reasons or had a fold change of less than 1.5; ^⁪⁪^ genes that did not pass the statistical significance test but showed an average alteration of at least 1.5-fold. Fold change values are the averaged log_2_ ratio of normalized signal values from two independent statistical analyses. Abbreviations are as follows: Exp, Exponential Growth Phase; Stat, Stationary Growth Phase; STM, Signature Tagged Mutagenesis.

**References**

1. Uzureau S, Lemaire J, Delaive E, Dieu M, Gaigneaux A, Raes M, De Bolle X, Letesson JJ: **Global analysis of Quorum Sensing targets in the intracellular pathogen *Brucella melitensis* 16M**. *J Proteome Res* 2010, In press.

2. Kohler S, Foulongne V, Ouahrani-Bettache S, Bourg G, Teyssier J, Ramuz M, Liautard JP: **The analysis of the intramacrophagic virulome of *Brucella* *suis* deciphers the environment encountered by the pathogen inside the macrophage host cell**. *Proc Nat Acad Sci U S A* 2002, **99**(24):15711-15716.

3. Rambow-Larsen AA, Rajashekara G, Petersen E, Splitter G: **Putative quorum-sensing regulator BlxR of *Brucella* *melitensis* regulates virulence factors including the type IV secretion system and flagella**. *J Bacteriol* 2008, **190**(9):3274-3282.

4. Delrue RM, Lestrate P, Tibor A, Letesson JJ, De Bolle X: ***Brucella* pathogenesis, genes identified from random large-scale screens**. *FEMS Microbiol Lett* 2004, **231**(1):1-12.

5. Wu Q, Pei J, Turse C, Ficht TA: **Mariner mutagenesis of *Brucella melitensis* reveals genes with previously uncharacterized roles in virulence and survival**. *BMC Microbiol* 2006, **6**:102.

6. LeVier K, Phillips RW, Grippe VK, Roop RM, 2nd, Walker GC: **Similar requirements of a plant symbiont and a mammalian pathogen for prolonged intracellular survival**. *Science* 2000, **287**(5462):2492-2493.

7. Haine V, Sinon A, Van Steen F, Rousseau S, Dozot M, Lestrate P, Lambert C, Letesson JJ, De Bolle X: **Systematic targeted mutagenesis of *Brucella melitensis* 16M reveals a major role for GntR regulators in the control of virulence**. *Infect Immun* 2005, **73**(9):5578-5586.

8. Lestrate P, Dricot A, Delrue RM, Lambert C, Martinelli V, De Bolle X, Letesson JJ, Tibor A: **Attenuated signature-tagged mutagenesis mutants of *Brucella* *melitensis* identified during the acute phase of infection in mice**. *Infect Immun* 2003, **71**(12):7053-7060.
